# Consensus clustering of gene expression profiles in peripheral blood of acute ischemic stroke patients

**DOI:** 10.3389/fneur.2022.937501

**Published:** 2022-08-05

**Authors:** Zhiyong Yang, Guanghui Wang, Nan Luo, Chi Kwan Tsang, Li'an Huang

**Affiliations:** ^1^Clinical Neuroscience Institute, The First Affiliated Hospital of Jinan University, Guangzhou, China; ^2^Department of Neurology, The First Clinical Medical School of Jinan University, Guangzhou, China

**Keywords:** acute ischemic stroke, molecular subgroups, peripheral immunity, restricted cubic spline functions, immunosuppression, consensus clustering

## Abstract

Acute ischemic stroke (AIS) is a primary cause of mortality and morbidity worldwide. Currently, no clinically approved immune intervention is available for AIS treatment, partly due to the lack of relevant patient classification based on the peripheral immunity status of patients with AIS. In this study, we adopted the consensus clustering approach to classify patients with AIS into molecular subgroups based on the transcriptomic profiles of peripheral blood, and we identified three distinct AIS molecular subgroups and 8 modules in each subgroup by the weighted gene co-expression network analysis. Remarkably, the pre-ranked gene set enrichment analysis revealed that the co-expression modules with subgroup I-specific signature genes significantly overlapped with the differentially expressed genes in AIS patients with hemorrhagic transformation (HT). With respect to subgroup II, exclusively male patients with decreased proteasome activity were identified. Intriguingly, the majority of subgroup III was composed of female patients who showed a comparatively lower level of AIS-induced immunosuppression (AIIS). In addition, we discovered a non-linear relationship between female age and subgroup-specific gene expression, suggesting a gender- and age-dependent alteration of peripheral immunity. Taken together, our novel AIS classification approach could facilitate immunomodulatory therapies, including the administration of gender-specific therapeutics, and attenuation of the risk of HT and AIIS after ischemic stroke.

## Introduction

Acute ischemic stroke (AIS) is one of the leading causes of death and disability worldwide, affecting approximately 10 million people each year and resulting in an enormous economic burden for AIS treatment and post-stroke care (1, 2). Recent studies have demonstrated that the immunomodulatory therapy targeting peripheral immunity is promising for AIS treatment, including attenuation of hemorrhagic transformation (HT) or AIS-induced immunosuppression (AIIS) (3–5). However, there is a complex relationship between peripheral immunity states and stroke pathology. Without careful consideration of AIS patient's peripheral immunity state, broad suppression or modulation of peripheral immunity may impair the normal physiological immune functions, such as the clearance of damaged tissues, subsequent repair responses, or protection against systemic infections, which would result in worsening of stroke prognosis (4, 5). For example, the Enlimomab Acute Stroke Trial, a randomized controlled trial of a murine monoclonal antibody to ICAM-1 for blocking peripheral immunity responses, has shown that the Enlimomab-treated patients exhibited significantly worse outcomes with a higher incidence of fever, infections, and death (6). By far, there are no clinically approved immunotherapeutic drug available (7, 8), partly due to the heterogeneous nature of peripheral immunity states in patients with AIS (9, 10). These results highlight the importance of classification of patients with AIS based on their peripheral immunity states to optimize the efficacy of immunotherapies.

Accumulating evidence has shown that subgrouping patients based on gene expression patterns of peripheral blood plays a key role in patient selection for successful clinical translation of immune interventions in multiple diseases including cancers, systemic lupus erythematous, and sepsis (11–13). In addition, peripheral blood can be conveniently obtained from patients with a quick and easy venous blood collection. As a core component of peripheral immunity, peripheral blood cells such as lymphocytes, monocytes, and granulocytes are intimately involved in many immune responses after AIS (14, 15). Moreover, gene expression profiling of peripheral blood could provide crucial information for the peripheral immunity state after stroke (16, 17). Therefore, we hypothesized that transcriptomic profiling of peripheral blood could be used to establish novel AIS classification for providing important insights into the immune interventions for stroke treatment. In this study, we employed a consensus-clustering approach to classify patients with AIS into major molecular subgroups based on their gene expression profiles in peripheral blood, and we characterized these subgroups by various integrative analyses. To further investigate the role of age and gender in the peripheral immunity states of patients with AIS, we employed a restricted cubic spline analysis to elucidate their relationships with subgroup-specific gene expression. Finally, we evaluated the implication of AIS classification on correlation with hemorrhagic transformation (HT) in patients with AIS, aiming to understand the role of peripheral immunity in HT and to provide new insights into the immune interventions for stroke treatment.

## Materials and methods

### Data collection

Two datasets (GSE16561 and GSE37587) were extracted from the Gene Expression Omnibus (GEO) by R/Bioconductor package GEOquery, including the gene expression matrix, clinical characteristics, and probe sets. The dataset of samples drawn from non-stroke neurologically healthy participants was used as the control. GSE199435 was extracted from the gene expression dataset of blood samples collected from AIS patients with HT or non-HT (*n* = 3 for each group). The fastq RNA-sequencing data were downloaded from the SRA database (SRP365953). FeatureCounts was used to count the reads mapped to individual genes by processing the sorted bam files with accepted read quality for subsequent analysis.

### Removal of batch effect

Since GSE16561 and GSE37587 datasets used the same platform (GPL6883 platform Illumina HumanRef-8 v3.0 expression beadchip), gene expression data were merged for analysis in our study. The expression values merged from both datasets were log_2_ transformed, and then ComBat normalization in the SVA package was used to remove batch effects.

### Consensus clustering

Consensus clustering was performed by “*ConsensusClusterPlus*” R package as an unsupervised resampling analysis for the detection of high consensus optimal molecular subgroups based on intrinsic molecular characteristics of gene expression features, through robust clustering across multiple runs of a clustering algorithm without external information to mitigate the limitations of individual clustering algorithms by averaging over various clustering methods with random or subjectivity (70). The clustering was performed by a K-means algorithm with the Spearman distance. The maximum cluster number was set to 10. The final cluster number was determined by the consensus matrix and the cluster consensus score (>0.8), as recommended in the literature (70).

### Comparing the clinical characteristics of subgroups

The proportion of male patients and different sampling times (within 24 h and 24–48 h after AIS) in the subgroups were compared using the pairwise proportion test. Given that male and female patients may have different patterns of correlation with age, gender-stratified analyses were performed (18). Pairwise Wilcoxon's rank sum test was used to test whether there was a difference in age among the male and female patients in the subgroups. Since the age of female subjects in subgroups showed no significant difference in Wilcoxon's rank sum test, we used logistic regression models with restricted cubic spline functions to test the potential non-linearity between female age and subgroups as described previously (71).

### Identification of subgroup-specific upregulated and downregulated genes

Subgroup-specific upregulated and downregulated genes were identified by comparing the samples in a specified subgroup with samples in the other subgroups using the Wilcoxon rank-sum test (19, 20). To avoid obscuring gene expression features in pathway enrichment analyses and identify the most valuable differentially expressed genes, we used the cutoff criteria defined by Benjamini-Hochberg and adjusted it to *p* < 0.05 and the absolute difference of means > 0.2 (19, 20). Upregulated genes were defined as the subgroup-specific genes with a difference of means > 0.2, while downregulated genes were subgroup-specific genes with a difference of means < −0.2. For any given gene, the difference in mean was calculated by subtracting the mean of expression in the other group from that in the samples of the specified subgroup.

### Weighted gene co-expression network analysis

Weighted gene co-expression analysis (WGCNA) was performed under subgroup-specific signatures to identify potential modules with characterized biological functions in each subgroup (21). *FlashClust* function (in “*WGCNA*” package) was used to carry out cluster analysis of samples with appropriate threshold to detect and remove the outliers. The soft thresholding power β-value was screened during module construction by the pick Soft Threshold function in the “*WGCNA*” package. The power of β = 9 (scale-free *R*^2^ > 0.8) was selected as the soft-thresholding parameter. The topological overlap matrix similarity was used to evaluate the distance between each gene pair. Hierarchical clustering analysis with the average and dynamic methods was used to build the cluster tree and classify the genes into modules. After merging the original modules based on their similarity, we finally identified eight modules. In the “*WGCNA*” package, subgroups and gender were converted to numerical values followed by a regression analysis with module eigengene values to visualize the expression tendency of modules. For gender-stratified analysis, Spearman's correlation coefficients and the corresponding *p*-values between age and modules eigengene values were calculated by the “*WGCNA*” package. An alluvial diagram was used to visualize the main distribution and associations between subgroup-specific upregulated and downregulated genes and genes in the eight functional modules using the R software package “*ggalluvial*” and “*ggplot2*.”

### Pre-ranked gene set enrichment analysis

Gene set enrichment analysis (GSEA) was performed in GSEA Desktop v4.1.0 with GSEAPrerank mode and a false discovery rate (FDR) < 0.05. The gene set databases were generated from the subgroup-specific signature genes. The gene list for each subgroup was ranked by negative log_10_-transformed adjusted *p*-values for Wilcoxon rank-sum test, which was calculated as each subgroup vs. normal control. We employed a restricted cubic spline analysis to determine the non-linear relationship between co-expression modules and log-transformed age in different gender for pre-ranked GSEA analysis as described (22, 23). To cover the most valuable differentially expressed genes (DEGs) of GSE199435 in the pre-ranked GSEA, we adjusted the cut-off value to *p* < 0.05 and logFC > 0.2 in comparison between AIS patients with HT and patients without HT (non-HT), according to DESeq2. Based on the ranking metric of logFC, the concordance in transcriptional profile between subgroup-specific genes of each WGCNA module and the transcripts was performed by the pre-ranked GSEA (24, 25).

### Functional module enrichment analysis

Kyoto Encyclopedia of Genes and Gene Ontology (KEGG) and Reactome pathway enrichment analyses for the subgroup-specific upregulated and downregulated genes were performed for genes in the modules using “*clusterprofiler”* and “*ReactomePA”* R package (26, 27). The pathways with *P* < 0.05 were considered statistically significant for functional modules.

## Results

**Figure 1:**
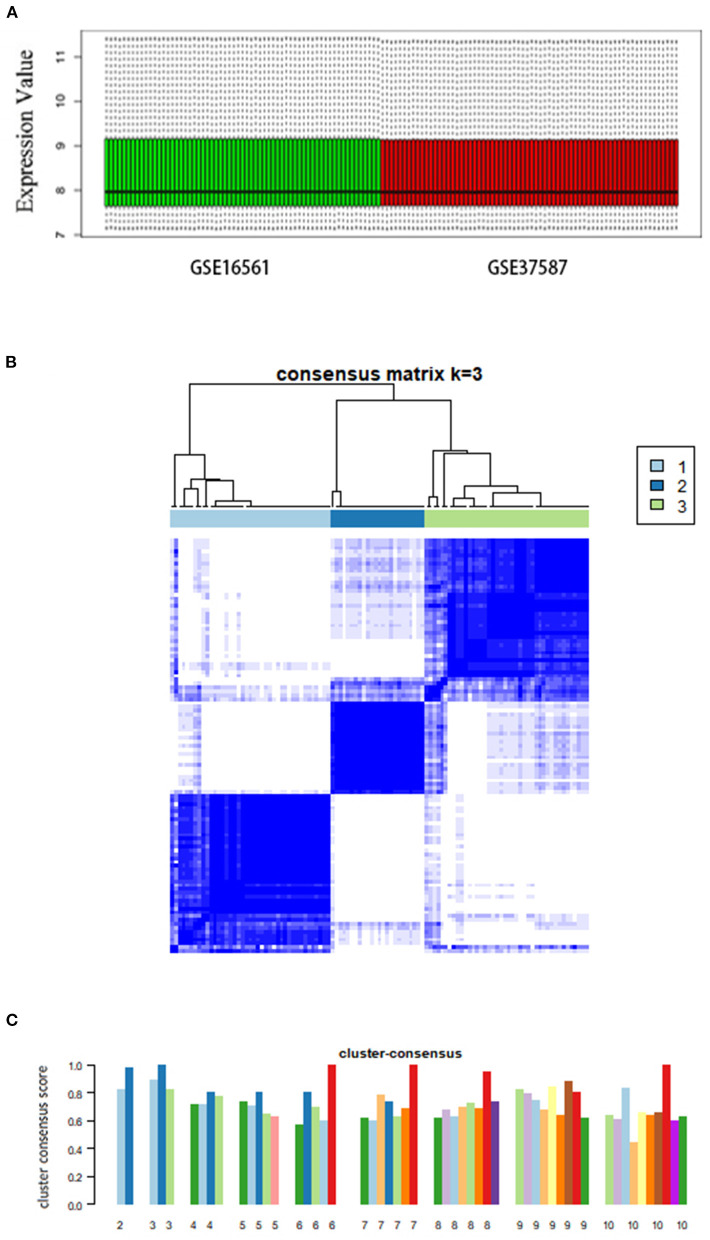
Consensus clustering analysis of gene expression profiles of peripheral blood samples in patients with AIS. **(A)** Box plots showing the normalized relative expression (y-axis) of data from genesets GSE16561 and GSE37587. **(B)** The heatmap representing the consensus matrix with a cluster count of 3, which was determined by the minimal consensus scores of >0.8. **(C)** Bar-plots showing the consensus scores (y-axis) and the corresponding numbers of the subgroup. The maximum number of subgroups was set to 10.

We analyzed a total of 131 peripheral blood samples collected from 107 patients with AIS and 24 healthy individuals from two independent datasets (GSE16561 and GSE37587). We used the ComBat method to process 18,599 genes and eliminated the batch effect by normalization (Figure 1A).

### Consensus clustering of AIS cases

Based on the unsupervised consensus clustering method, we classified the 107 patients with AIS into three subgroups based on their gene expression profiles. Subgroups I, II, and III consisted of 41, 24, and 42 patients with AIS, respectively. We showed that the gene expression pattern of each subgroup is highly specific by consensus matrix analysis (Figure 1B). The minimal consensus score analysis for subgrouping indicated that three groups would provide robust classification based on the criteria of cluster count with a consensus score > 0.8 (Figure 1C) (70).

**Figure 2:**
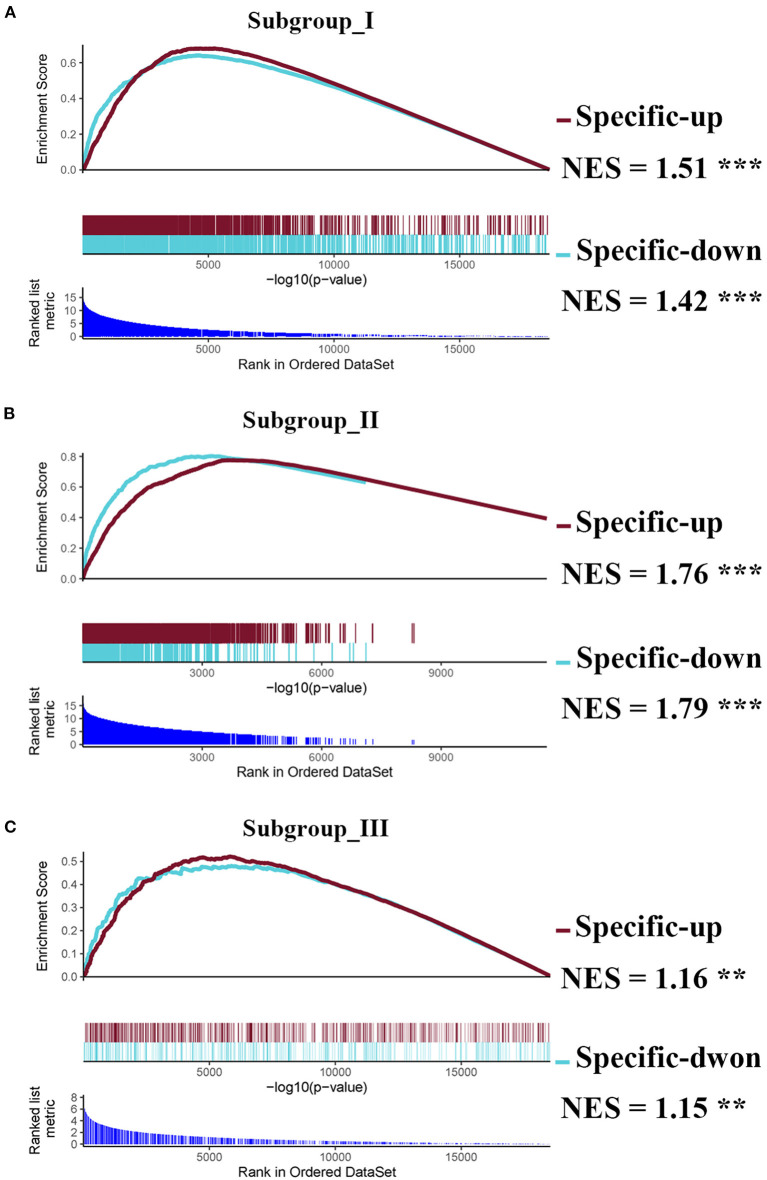
Identification of subgroup-specific differentially expressed genes and comparison with control. **(A–C)** GSEA enrichment plots (red lines) showing the subgroup-specific upregulated gene (Specific-up) and subgroup-specific downregulated gene (Specific-down) in subgroup I **(A)**, subgroup II **(B)**, and subgroup III **(C)**. Green lines represent the differentially expressed genes between the corresponding subgroup and the normal controls. NES denotes the normalized enrichment score with FDR (** <0.01, and *** <0.001).

Next, we performed the pair-wise differential expression analysis of each subgroup with the other two subgroups by GSEA. As shown in Figure 2A, we detected 1,372, 1,012, and 712 genes showing subgroup-specific upregulation, as well as 1,363, 609, and 337 genes showing downregulation in subgroups I, II, and III, respectively (Benjamini-Hochberg adjusted *p* < 0.05 and the absolute difference of means > 0.2, Supplementary Figure S1). To investigate the specificity of these differentially expressed genes in each subgroup, we performed a GSEA analysis to compare their gene expression profiles with that of the normal control. The normalized enrichment score revealed that the subgroup-specific genes were also significantly and differentially expressed when compared with the control group (Figures 2A–C, FDR <0.05).

### Characterization of subgroups

**Figure 3:**
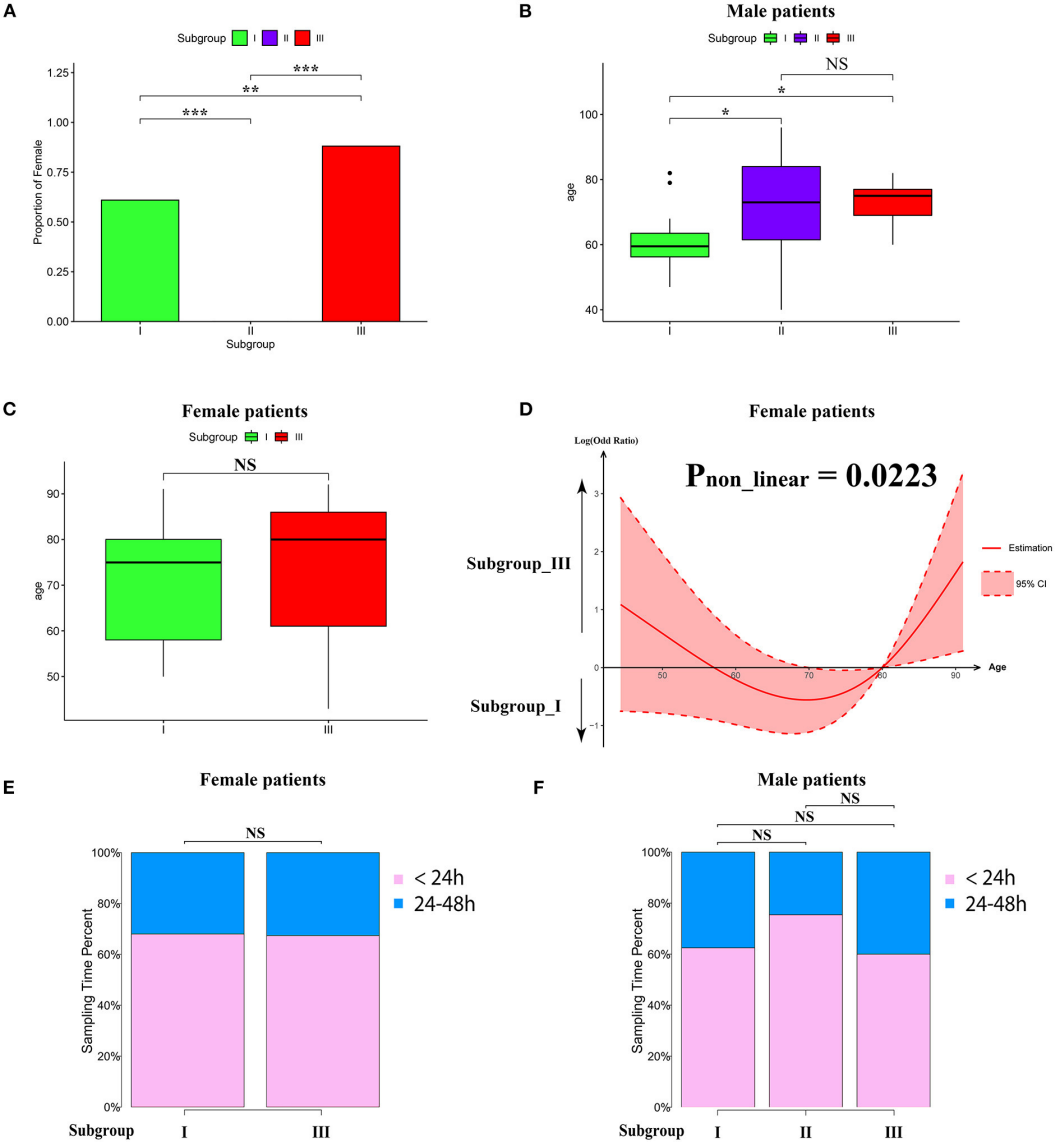
Clinical characteristics within subgroups. **(A)** Barplot showing the proportion of male patients in each subgroup. **(B,C)** Barplot showing the age distribution in each subgroup in male **(B)** and female patients **(C)**. Values are means ± SD. NS indicates no significant difference between groups (*p* > 0.05). *P < 0.05, **P < 0.01, and ***P < 0.001. **(D)** Non-linear relationship between subgroup and the age of female patients analyzed by restricted cubic spline model. The black line represents the pooled odds ratio (OR), and the red lines indicate a 95% confidence interval (CI). **(E,F)** Box-plots showing the proportion of sampling time (within 24 h and between 24 and 8 h) after AIS in each subgroup of female **(E)** and male **(F)** patients. NS indicates no significant difference between groups (*p* > 0.05).

To characterize the clinical features of the three subgroups, we analyzed the distribution of gender, age, and sampling time after AIS in each subgroup. Intriguingly, the gender distribution among the subgroups was very different. As shown in Figure 3A, the proportion of women in subgroup III was significantly higher than that in subgroup I. Intriguingly, subgroup II consisted of men only (Figure 3A). Therefore, we separated male and female patients for age analysis. We found that the average age of male subjects in subgroup I was significantly younger than subgroups II and III (Figure 3B). In contrast, there was no significant difference between the average age of female subjects in subgroups I and III, as determined by Wilcoxon's rank sum test (Figure 3C). To explore whether there was a non-linear relationship, we performed a logistic regression model analysis with restricted cubic spline (RCS) functions, which revealed a U-shaped relationship between female age and subgroups, without significant linearity (P_non−linear_ = 0.0223; Figure 3D). To determine whether the sampling time would affect the pattern of AIS subgrouping, we separated patients into 2 groups (sampling time during 24–48 h and within 24 h), and there was no significant difference among the subgroups in female or male cases (Figures 3E,F).

**Figure 4:**
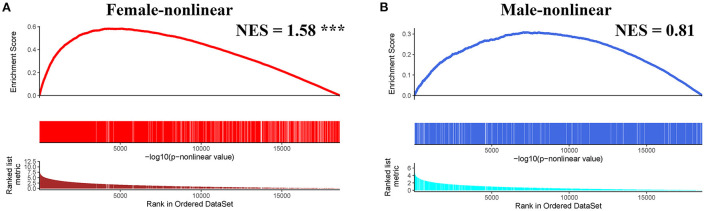
GSEA analysis of subgroup-specific differentially expressed genes. **(A)** The enrichment plot illustrates that most subgroup-specific genes had a significantly non-linear relationship with female age. The normalized enrichment score (NES) and FDR (*** <0.001) in the female group. **(B)** The enrichment plot illustrates that most subgroup-specific genes had a significantly non-linear relationship with male age. The normalized enrichment score (NES) is shown without significant difference **(**FDR > 0.05).

Next, we conducted pre-ranked GSEA and restricted cubic splines analysis to determine the correlation between age and subgroup-specific gene expression across female and male patients. As shown in Figure 4, the subgroup-specific genes were concentrated in the gene sets with a non-linear correlation with the corresponding age in female patients with AIS (NES = 1.58, FDR < 0.001), but not in male patients (NES = 0.81, no significant difference). These results demonstrate that gender and age are determining factors for gene expression in the peripheral blood of patients with AIS.

### Identification of gene co-expression modules

**Figure 5:**
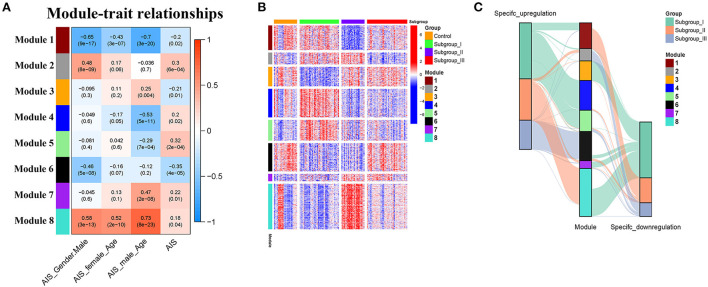
Identification and characterization of 8 modules by weighted gene co-expression analysis (WGCNA). **(A)** Module-trait relationship of gender and age in male and female patients. The positive and negative correlation coefficients of WGCNA modules and clinical characteristics are colored from red to blue. Each row corresponds to a module eigengene, each column corresponds to a trait. Each cell depicts the corresponding Pearson correlation r-values and p values in the bracket. **(B)** The scaled expression values of genes in each of the 8 WGCNA modules are displayed in the heatmap. **(C)** Alluvial diagram showing the inherent relationship between modules and subgroups.

**Figure 6:**
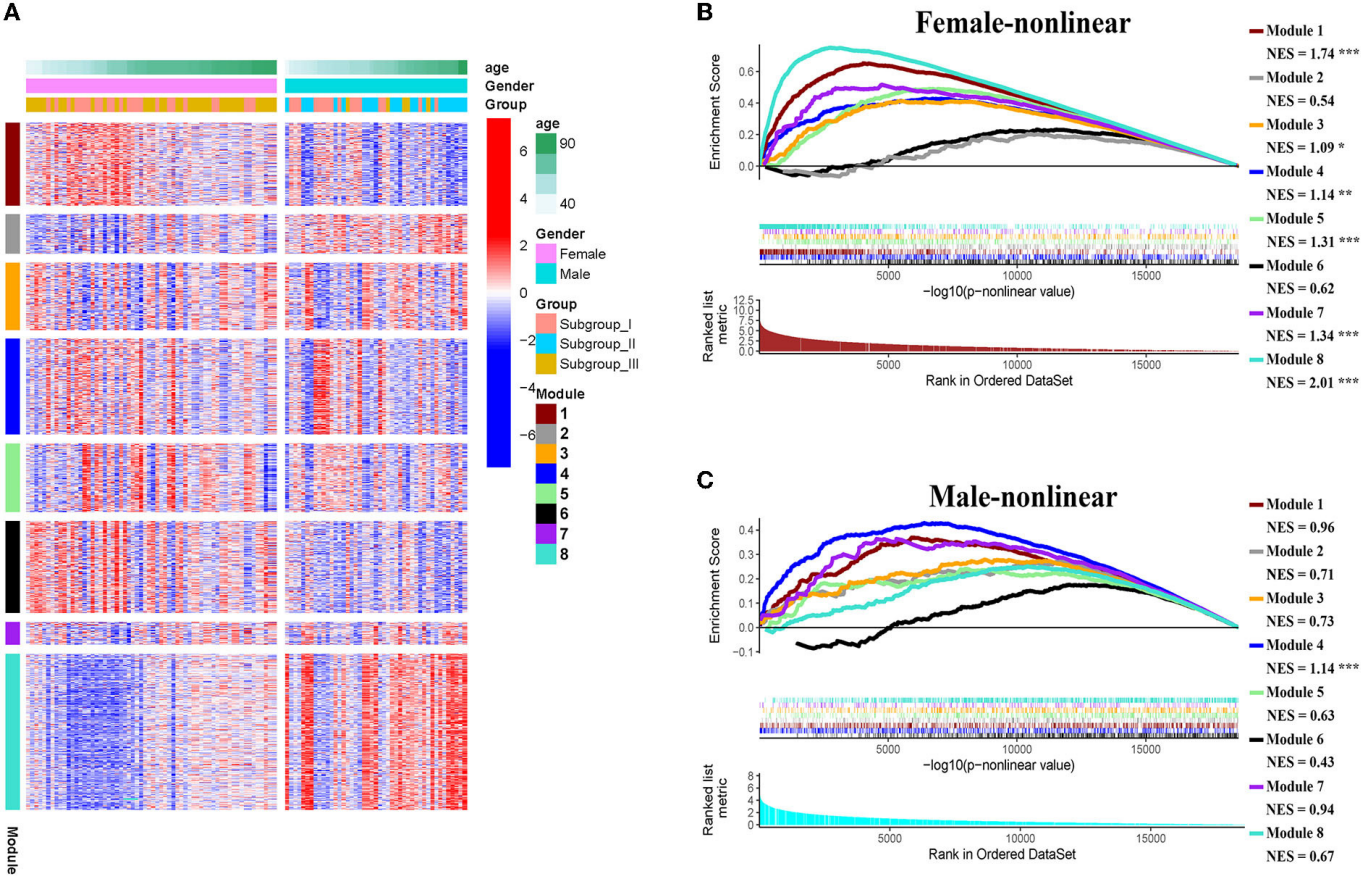
**(A)** Heatmap showing association of gender-specific distribution of age with the expression of subgroup-specific genes in each module. Red and blue indicate upregulation and downregulation, respectively. Samples were ordered by increasing age in each gender. **(B,C)** The enrichment plots of the restricted cubic spline-GSEA showing the significance of the non-linear relationship between all normalized expression of genes in each module and log-transfer age in female patients **(B)** and male patients **(C)**.

The weighted gene co-expression network analysis (WGCNA) is a powerful tool to identify modules with a coordinated expression of genes with similar patterns (36). In an attempt to discover unique and specific subgroup signatures, we performed WGCNA of all subgroup-specific genes and control. The specific expression tendency revealed 8 WGCNA modules (Figures 5A,B; Supplementary Table S1). In addition, the cluster analysis by *flashClust* revealed no outlier samples (Supplementary Figure S2A). The power of β was set at 9 to ensure a scale-free network based on the scale-free fit index and the mean connectivity (Supplementary Figure S2B). The topological overlap measure (TOM) for each gene pair was then calculated. Consistently, the hierarchical clustering analysis based on the TOM dissimilarity measure (1-TOM) also revealed eight modules (Supplementary Figure S2C). Then, we used an alluvial diagram to visualize the main distribution and associations between subgroup-specific upregulated and downregulated genes and genes from eight modules (Figure 5C). Based on the main distribution (color bars) and association (lines) of WGCNA modules with the subgroup-specific genes, we identified the upregulated and downregulated associations between WGCNA modules and the corresponding primary subgroups (Table 1). To investigate the relationship between clinical features and gene expression of WGCNA modules, we performed the Pearson correlation analysis, which verified that all modules were correlated with AIS, and this served as the positive control. Importantly, module 6 exhibited the highest degree of negative correlation with AIS (r-value = −0.35), while modules 2, 4, 5, and 7 showed comparable levels of positive correlation with AIS with r-values ≥ 0.2 (Figure 5A). With respect to subgroup I, the specific genes from modules 3, 4, 5, and 7 appeared to be independent of gender in patients with AIS (Figure 5A *p* > 0.05 in AIS_Gender_Male column). Notably, we observed a linear relationship between co-expression modules and age in male patients with AIS (except modules 2 and 6) (Figure 6A). In contrast, modules 1 and 8 showed significant linear correlation as shown in the corresponding age bracket in AIS female patients (Figure 6A). Apart from modules 2 and 6, we ascertained the non-linear relationship between most co-expression modules and log-transformed age in female patients with AIS through the incorporation of the restricted cubic spline analysis with pre-ranked GSEA (Figure 6B). Likewise, the accumulation of gene sets pertaining to a non-linear correlation in the corresponding age in male patients with AIS was not observed except in module 4 (Figure 6C). The linear and non-linear relationships between age and relevant modules in patients with AIS are visualized in the heatmap presentation as shown in Figure 6A. Together with the observation that modules 2 and 6 were primarily distributed in subgroup III-specific genes (Figure 5C), these results highlight the correlation of gender, rather than age, with gene co-expression in modules.

**Table 1:** The number of differentially expressed genes by case-control and case-case comparisons and co-expression modules in each subgroup.

**Subgroup**	**Subgroup-** **control** **comparison**	**Subgroup-specific** **upregulation** **genes**	**Subgroup-specific** **downregulation** **genes**	**Modules**	
				**Up**	**Down**
Subgroup_I	4,092	1,372	1,363	4 and 5	3, 7, and 8
Subgroup_II	2,945	1,012	609	8	1
Subgroup_III	898	712	337	6	2

### Functional enrichment analysis of genes in the co-expression modules

**Figure 7:**
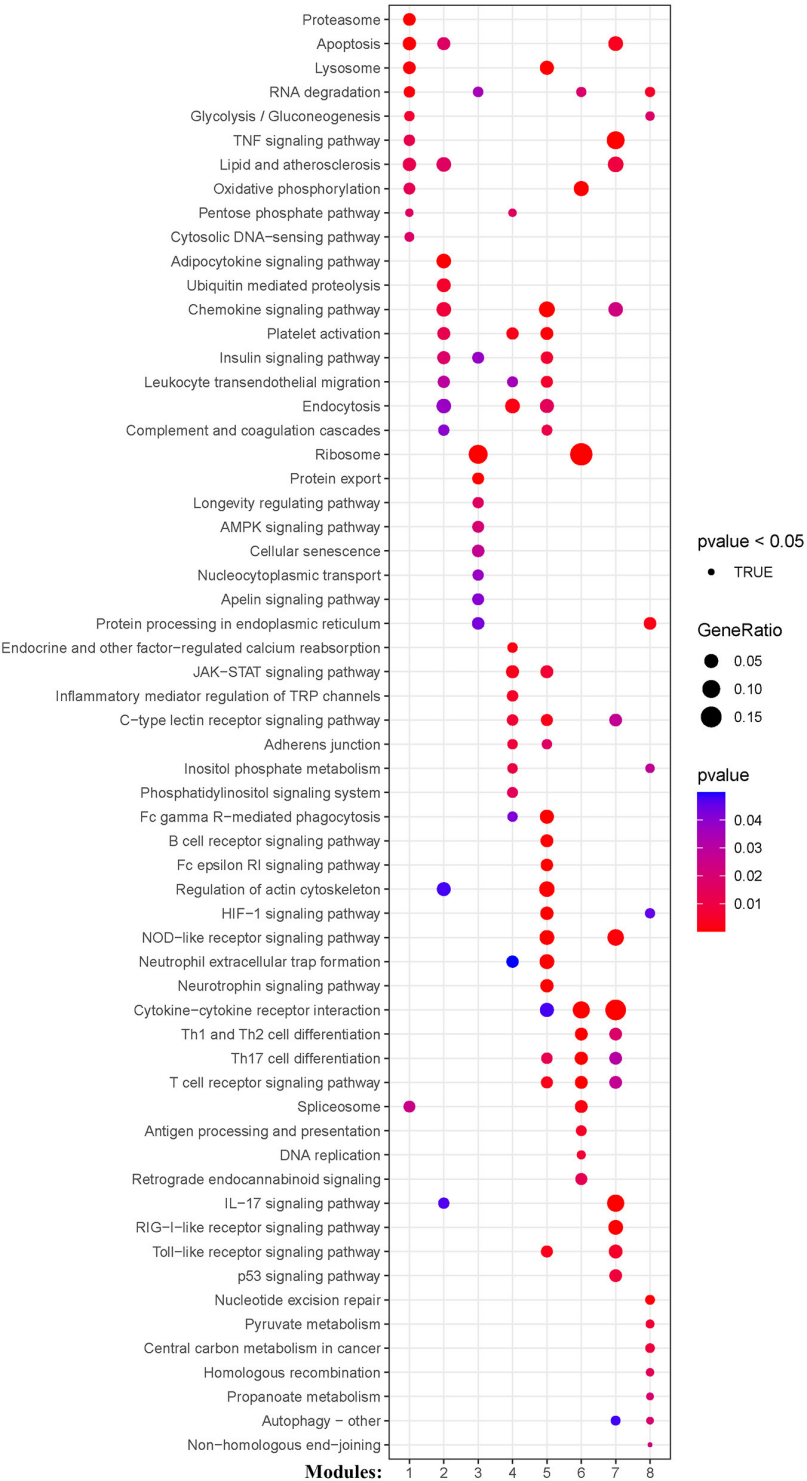
Module-based pathway analysis. Visualization of pathways identified by the Kyoto Encyclopedia of Genes and Genomes (KEGG) analysis.

**Figure 8:**
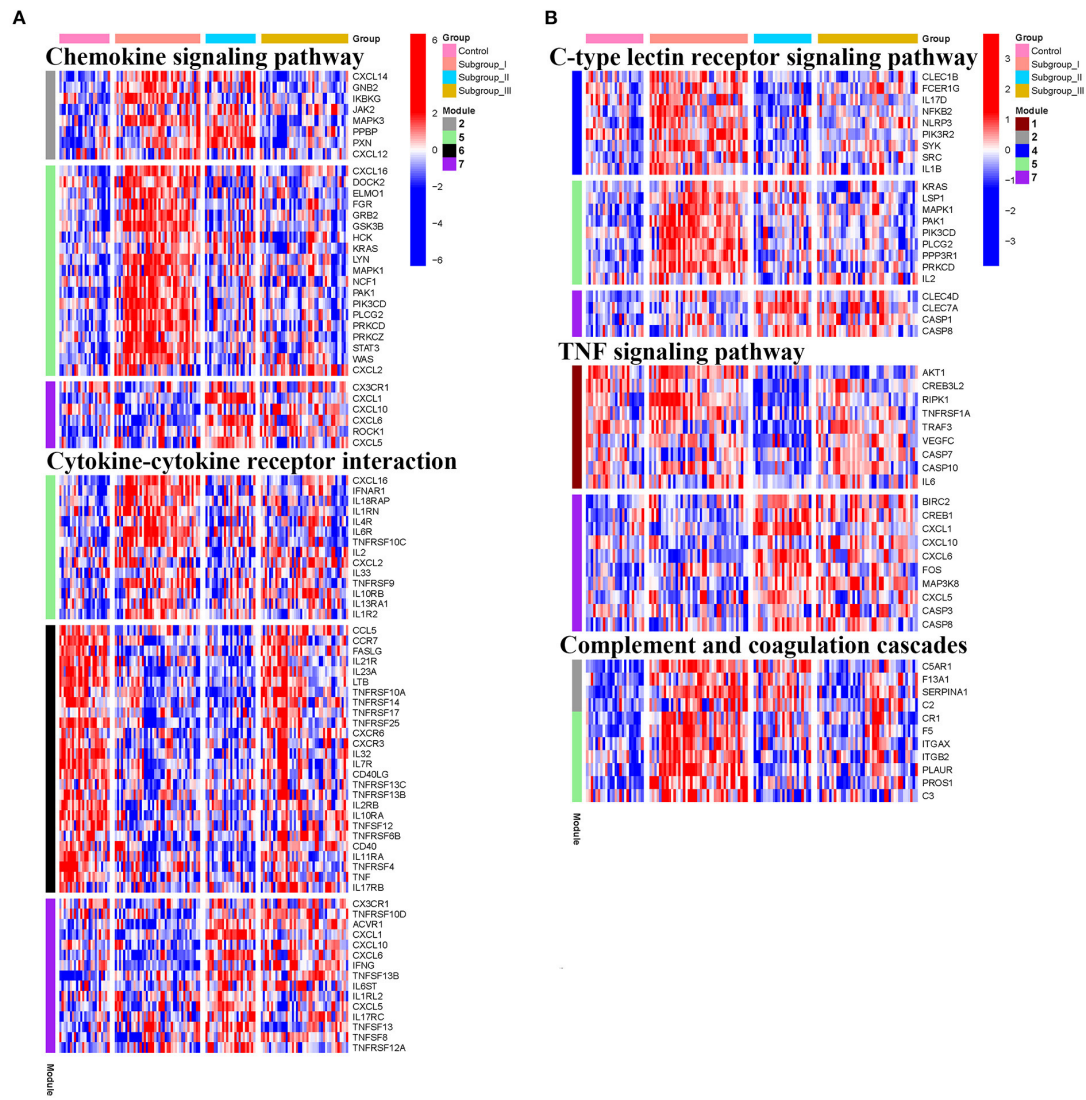
Heatmap showing the scaled expression values of genes in representative subgroup-specific KEGG pathways related to inflammation and corresponding signature of each co-expression module among subgroups. **(A)** Chemokine signaling pathway and cytokine-cytokine receptor interation. **(B)** C-type lectin receptor signaling pathway, TNF signaling pathway, and complement and coagulation cascades. Heatmap colors correspond to the level of mRNA expression as indicated in the color rang.

**Figure 9:**
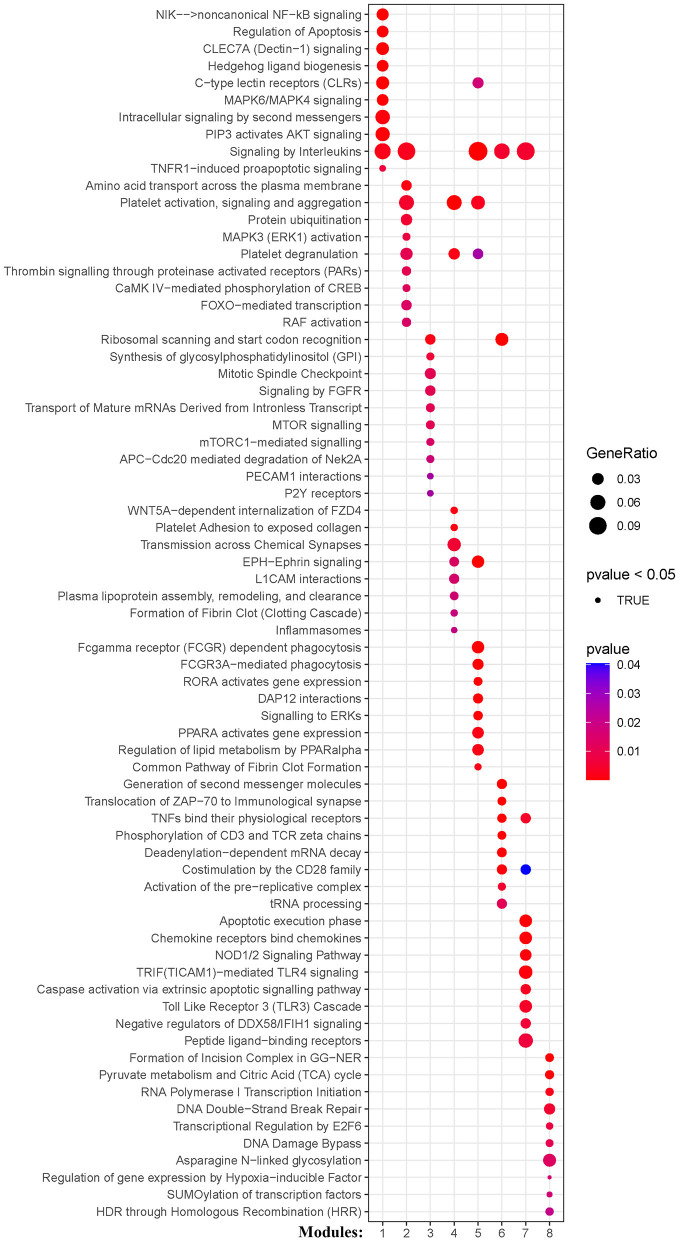
Module-based pathway analysis. Visualization of pathways identified by the Reactome.

**Figure 10:**
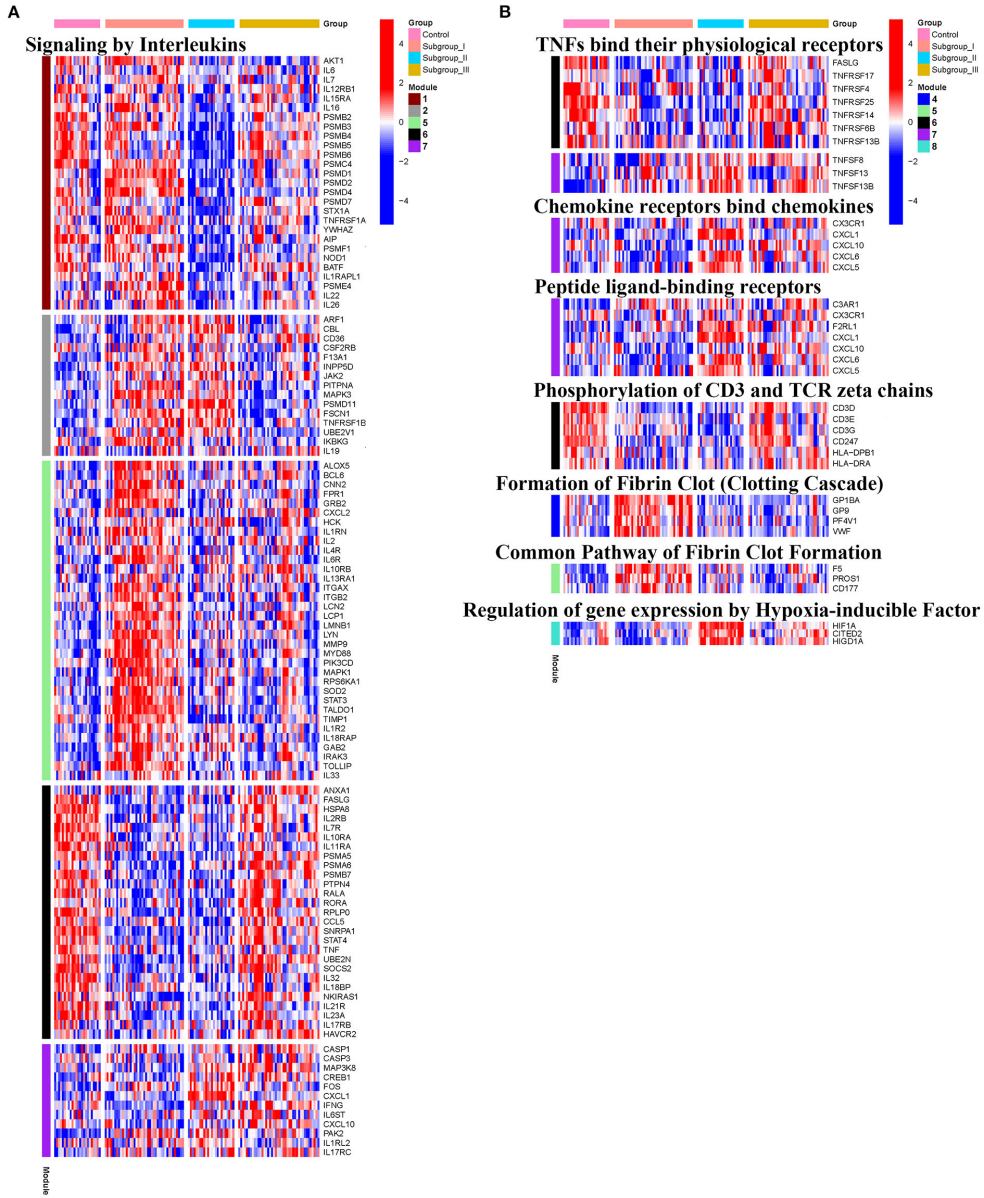
Heatmap showing the expression level of genes in representative subgroup-specific Reactome pathways related to inflammation as indicated in Signaling by Interleukins **(A)** and TNFs bind their physiological receptors, Chemokine receptors bind chemokines, Peptide ligand-binding receptors, Phosphorylation of CD3 and TCR zeta chains, Formation of Fibrin Clot Formation, and Relation of gene expression by Hypoxia-inducible Factor **(B)** and corresponding signature of each co-expression module among subgroups, heatmap colors correspond to the level of mRNA expression as indicated in the color rang.

KEGG and Reactome pathway analyses were used to identify enriched pathways in the co-expression modules. Interestingly, we found that many of them were related to inflammatory factors, complement synthesis, coagulation factor production, and the expression of C-type lectin receptors (Table 1; Figures 7–10). Notably, modules 4 and 5 primarily constituted of the subgroup I-specific upregulated genes. They were substantially enriched in pathways involved in neutrophil extracellular trap formation, inflammasomes, and metabolism of lipid and lipoprotein (Supplementary Figures S3,S4). With respect to module 4, we found the integration of NLRP3 with the inflammasome pathway (Supplementary Figure S4A). In modules 4 and 5, enriched pathways included platelet activation, thrombus formation, platelet adhesion to exposed collagen, platelet activation, formation of fibrin clot (clotting cascade), and common pathway of fibrin clot formation (Supplementary Figures S3,S4). Analysis of downregulated genes in subgroup I in modules 3, 7, and 8 revealed pathways related to the regulation of the immunocoagulation tangles (Supplementary Figures S3,S4). We also identified the regulation of gene expression by hypoxia-inducible factors in module 8, which suggest improved perfusion and enhanced arterial remodeling in this module (Figures 9, 10B) (28). Additionally, these pathway analyses revealed functions such as nucleotide excision repair, DNA damage bypass, HDR through Homologous Recombination (HRR), and DNA double-strand break repair in tandem with the increased activity of hypoxia-inducible factor and SUMOylation in module 8 (Figure 9), suggesting the anti-hypoxic injury by regulation of gene expression as shown in module 8 (Figures 7–10). Interestingly, module 1 showed the downregulation of genes enriched in proteasome activity in subgroup II (Figure 7, Supplementary Figure S3A). Furthermore, module 6 was related to phosphorylation of CD3 and TCR zeta chains in subgroup III (Figure 9). Genes in this pathway included CD3D, CD3E, CD3F, and CD247 (CD3ζ) which are also the representative markers of T-cells (Figure 10B). We also identified the enhanced normal T-cell function and comparatively lower levels of AIIS in subgroup III (Figure 10) (3). From module 2, specific-downregulated genes in subgroup III encompassed the enrichment of calmodulin-dependent kinase-related genes including CAMK2A and CAMKK2 (29, 30). Consistently, they are known as the fundamental regulators of immune function in conjunction with the enrichment of platelet activation and coagulation-related pathways (Supplementary Figure S4A) (31–33). The identification of these pathways in subgroup III-specific modules 2 and 6 demonstrates the specific peripheral immunological activity in female patients with AIS (Figures 3A, 5C). Another interesting observation was the enrichment of disparate metabolism pathways. For instance, we identified ADH4, DLD, PDK1, ABAT, and SDHD enriched in module 8 that were related to propanoate metabolism, pyruvate metabolism, and tricarboxylic acid (TCA) cycle, whereas ALDOC, PFKI, ALDOA, TKT, RPIA, and FBPI were enriched in the pentose phosphate pathway in modules 1 and 4. These results suggest the diverse regulation of metabolism in periphery immune response in patients with AIS (Supplementary Figures S3,S4). Furthermore, we identified that modules 1 and 6 showed distinct co-expression patterns in relation to oxidative phosphorylation Supplementary Figure S3B), whereas modules 3 and 6 exhibited different co-expression of genes involved in ribosome biogenesis (Supplementary Figure S3C). Finally, we identified the enrichment pathways involved in protein processing in the endoplasmic reticulum (Figure 7, Supplementary Figure S4B), protein export, and ribosome activity in module 3 (Figures 5C, 7; Table 1; Supplementary Figures S3B,C). These results suggest that the ribosome-related pathways influence protein synthesis and secretion disorders in patients with AIS of subgroup I.

### Correlation of transcriptomic pattern in subgroup I with HT

**Figure 11:**
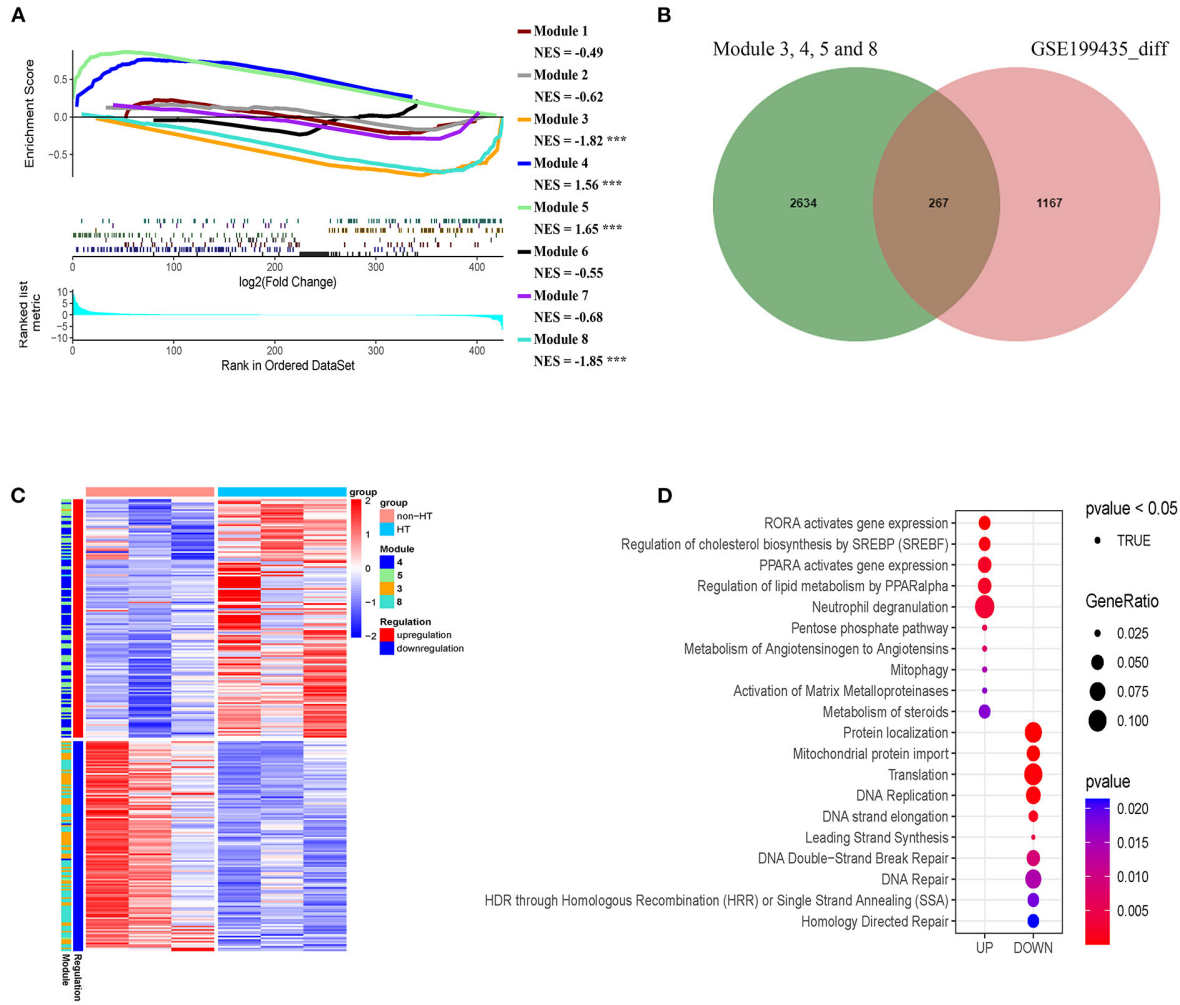
Genes in modules in subgroup I overlaps with hemorrhage transformation (HT)-specific differentially expressed genes (DEG). **(A)** Pre-ranked gene set enrichment analysis showing the enrichment of DEG related to HT in the co-expression modules with subgroup I-specific signature genes. **(B)** Venn diagram showing the number of overlapped genes (267) between HT-specific DEG and subgroup I-specific genes. **(C)** Heatmap representing the overlap genes between DEGs related to HT and subgroup I-specific genes. **(D)** Diagram showing pathways identified by Reactome with the negative log_10_ *p*-values of the overlapped genes shown in B and C.

Given that our identified modules in patients with AIS are closely associated with HT, we asked whether our classification could reveal the risk of HT in patients with AIS. To this end, we analyzed an independent dataset (GSE199435) which separated patients with AIS into HT and non-HT groups. We first identified the differentially expressed genes (DEG) in non-HT patients with AIS compared with those of HT. Then, we performed a pre-ranked gene set enrichment analysis to determine the enrichment of HT-related DEG to the co-expression modules with subgroup-specific signature genes. We found that a significant number of genes in modules 4 and 5 in subgroup I-specific upregulated genes were positively enriched in the HT-related upregulated genes (Figure 11A, NES > 1.5, FDR < 0.001). A similar number of genes in modules 3 and 8, mainly from subgroup I that were specific-downregulated genes, was negatively enriched in the HT-related downregulated genes (Figure 11A NES < −1.8, FDR < 0.001). In contrast, a smaller number of genes was observed when analyzing the non-subgroup I modules 1, 2, and 6 (Figure 11A). Therefore, we compared the DEG in modules 3, 4, 5, and 8 with those in HT-specific DEG. As shown in the Venn diagram in Figure 11B, we identified 267 genes in common (FDR < 0.05). As shown in the heatmap in Figure 11C, their differential expression pattern was highly similar and coordinated in these two sets of genes. Finally, we performed Reactome analysis for the identification of enriched pathways in these 261 genes. Results identified pathways including the activation of matrix metalloproteinases (MMP2 and MMP25), pentose phosphate pathway, DNA double-strand break repair, DNA repair, and translation (Figure 11D, Supplementary Figure S5). Taken together, these results suggest that subgroup I and AIS patients with HT share a similar transcriptional profile.

## Discussion

Acute ischemic stroke is a heterogeneous disorder with more than 100 pathologies implicated in its pathogenesis (34, 35). Therefore, it is important to develop various subgroup classification approaches for developing a personalized therapeutic strategy for effective stroke treatment. The advent of the subgroup classification such as the Trial of Org 10172 in Acute Stroke Treatment (TOAST), Causative Classification System (CCS), and Chinese Ischemic Stroke Subclassification (CISS) system during the past decade has facilitated the clinical application of antiplatelet and anticoagulation therapies (36, 37). In addition, the classification of AIS cases improves the clinical feasibility of time-dependent and population-specific revascularization therapies (38, 39). However, current AIS subgroup classification methods, mainly based on clinical evaluation and neuroimaging assessment, fail to address immune interventions that are promising but require personalized medications.

In this study, we developed a novel classification of AIS by consensus clustering of transcriptomic profiles in peripheral blood. Most of the subgroup-specific genes overlap with AIS signature genes as revealed by GSEA (Figure 2) and WGCNA (Figure 5A). These results highlight the importance of subgroup classification for a more precise correlation of peripheral immunity with AIS. Moreover, this study indicates that our subgroup classification is independent of sample collection time for the first 24 h or from 24 to 48 h, making our classification approach more flexible in terms of administration of gender-specific therapeutics for the attenuation of the risk of HT and AIIS, considering that the therapeutic time window of immune interventions is typically 6–48 h after AIS in clinical studies (40, 41).

Our transcriptome-based classification revealed subgroup-specific functional modules or pathways based on the tendency of co-expression patterns by WGCNA. As an integral component of the peripheral immunity response to AIS, our analysis confirmed that multiple inflammatory factors including chemokines, interleukins, and tumor necrosis factors are secreted from peripheral blood cells to trigger pro- or anti-inflammatory responses as reported previously (31, 42, 43). We also demonstrate that complement synthesis, coagulation factor production, and C-type lectin receptor expression in peripheral blood cells apparently play an instrumental role in peripheral immunity in response to AIS (44–46). Our findings also support the notion that AIS could regulate periphery immune response through the alteration of immunometabolism, including the tricarboxylic acid (TCA) cycle, the pentose phosphate pathway, propanoate metabolism, oxidative phosphorylation, and pyruvate metabolism (47–49). Our co-expression analysis further suggested a correlation between the immunometabolism and maintenance of DNA stability, consistent with recent studies showing that DNA double-strand break repair, DNA damage bypass, and homologous recombination were allegedly regulated by pyruvate metabolism and TCA cycle (50). In fact, the intricate roles of transcriptional reprogramming and alteration of immunometabolism in peripheral immune cells after AIS remain largely unknown. Our identification of various co-expression patterns within different subgroups could provide a comprehensive insight for future studies on immunotherapy for stroke.

It is worth noting that our pathway analysis in each module reveals the closely intertwined action of peripheral immunity-mediated inflammation, thrombosis-driven friability of cerebral vasculature, and coagulation disequilibrium. In agreement with these findings, recent studies have shown that the ischemic cerebral infarct progression is accompanied by microvascular thrombosis and subsequent HT (51, 52). In fact, these peripheral immunity-mediated inflammatory mechanisms have led to the concept that AIS is a systemic inflammatory and a thrombo-inflammatory disease (31–33). Interestingly, our GSEA analysis demonstrates that the coordinated expression pattern in the subgroup I-specific DEG is significantly analogous to that in the HT-specific modules (53). We further identified the activation of matrix metalloproteinases (including MMP2 and MMP25) that were enriched in the overlapping genes between these two groups, supporting their possible role in HT as reported previously (54–56). Moreover, we identified the pathways related to ribosome biogenesis and protein translation in both subgroups I and the HT-specific module. Currently, the role of protein translation in HT remains unknown, and obviously future studies will be required to provide the underlying mechanisms. Interestingly, our results suggest a potential mechanism of coagulation by peripheral immunity. We showed that modules 4 and 5, which are mainly from subgroup I specific upregulated genes, are all enriched in a pathway related to neutrophil extracellular trap (NET) formation, which is known to play a key role in hemorrhagic transformation and a thrombus resistant to lytic therapy (57, 58). In addition, our finding is in agreement with previous reports showing that the formation of NET may be induced by IL-1B, CAMP(LL37), and C3 in peripheral blood cells (59–62).

It is not uncommon in clinical settings that AIS induces AIIS, which is characterized by decreased CD3^+^T lymphocyte (3). Our pathway enrichment analysis shows that module 6, which is specifically upregulated in subgroup III (comprised mostly female patients) is plausibly correlated with a lesser degree of AIIS compared to subgroups I and II. Consistently, it has been reported that the female patients showed a reduced level of AIIS compared with male patients, suggesting a gender-specific response in post-AIS peripheral immunity (63–65). In addition, recent studies have shown a diminished level of T cell activity following the release of coagulation factor V(F5), arginase 1 (ARG1), LL33 (CAMP), and IL33 from the periphery immune cells, resulting in peripheral immunosuppression (66–69). We discover the lack of upregulation of ARG1, IL 33, and F5 in subgroup III, which denotes a contrary expression tendency in module 6 (see Supplementary Table S1). In contrast, module 2 consists of calmodulin-dependent kinase-related genes CAMK2A and CAMKK2, which are crucial regulators of peripheral blood cell functions (29, 30). It has been reported that peripheral blood cells are capable of producing complements and factors in coagulation cascades including C2, C5AR1, F13A1 (coagulation factor XIII, A1 Polypeptide), and SERPINA1 (a form of antiplasmin protein) (44). Interestingly, these genes are enriched in module 2 in our study, further supporting a correlation between thromboinflammation and AIIS. Finally, we found that the proteasome activity is decreased specifically in subgroup II. We speculate that peripheral blood cells in this AIS subgroup patients may reduce proteasome activity for alleviating ischemic injury. Since subgroup II is mainly composed of older male patients and subgroup III is mostly of female patients, this gender-dependent characteristic of classification by molecular subgrouping may guide personalized immunotherapeutic treatment for stroke.

Our study has several limitations. Firstly, we only examined 107 AIS cases, and a larger sample size in the future would be necessary to verify the robustness of our classification. Secondly, we could not directly evaluate the prognostic value using this classification method due to the lack of relevant clinical details in the datasets. For example, we were not able to relate the subgroups to clinical information such as comorbidities, demographic characteristics, thrombolysis or types of endovascular treatment, type of hemorrhage transformation (spontaneous HT, thrombolysis-induced HT, etc.), and TOAST classification. Therefore, we could not determine the predictive value of this classification on the prognosis of patients with AIS. Finally, samples were mainly collected from patients in the United States and China. Future prospective validation work is required by using samples collected from other countries.

In conclusion, the peripheral blood transcriptomic profiling for AIS classification reveals distinct and specific expression patterns. Considering the heterogeneity of the peripheral immunity in stroke patients, this molecular classification approach would have a prognostic impact by improving the efficacy of immunotherapies, prediction of the risk of hemorrhagic transformation, and therapeutic time window of immune interventions. Thus, this classification could potentially improve the prognosis of patients with AIS by providing a more informative and personalized medication strategy by the identification of patients with AIS who are vulnerable to stroke-induced immunodeficiency, resistant to gender-specific immunotherapy, and at risk of hemorrhagic transformation and other comorbidities.

## Data availability statement

The original contributions presented in the study are included in the article/Supplementary material, further inquiries can be directed to the corresponding authors.

## Ethics statement

Ethical review and approval was not required for this study in accordance with the local legislation and institutional requirements. Written informed consent from the patients/participants or patients/participants' legal guardian/next of kin was not required for this study in accordance with the national legislation and the institutional requirements.

## Author contributions

ZY: collection and analysis of data, and interpretation of data. ZY and CT: manuscript writing and revision. GW: project development. NL: technical assistance. CT and LH: direct the overall project. All authors contributed to the article and approved the submitted version.

## Funding

This research was funded by grants from the National Nature Science Foundation of China (Grant Number 81971120); the Science and Technology Plan Projects of Guangdong Province (Grant Number 2022A1515012563); the Basic and Applied Basic Research on the Project Jointly Funded by Guangzhou city and Jinan University (Grant Number 202201020062).

## Conflict of interest

The authors declare that the research was conducted in the absence of any commercial or financial relationships that could be construed as a potential conflict of interest.

## Publisher's note

All claims expressed in this article are solely those of the authors and do not necessarily represent those of their affiliated organizations, or those of the publisher, the editors and the reviewers. Any product that may be evaluated in this article, or claim that may be made by its manufacturer, is not guaranteed or endorsed by the publisher.
