# Supplementary material for: Consensus clustering of gene expression profiles in peripheral blood of acute ischemic stroke patients
Source: Front Neurol. 2022 Aug 5;13:937501. doi: 10.3389/fneur.2022.937501 (PMC9388856; doi:10.3389/fneur.2022.937501)
Supplement: Supplementary file 1 [file Table_1.DOC]

Supplemental Table S1. The details of subgroup‐specific genes with corresponding subgroups.

| Genes | specific-up | specific-down |
| --- | --- | --- |
| ENPP4 |  | Subgroup_I |
| RPS21 |  | Subgroup_I |
| IFI44 |  | Subgroup_I |
| CYBRD1 |  | Subgroup_I |
| MAFB |  | Subgroup_I |
| KIAA0323 | Subgroup_I |  |
| PLCD1 | Subgroup_I |  |
| GPA33 | Subgroup_I |  |
| ADORA2A | Subgroup_I |  |
| ST3GAL1 | Subgroup_I |  |
| CASP2 | Subgroup_I | Subgroup_II |
| FLJ11795 | Subgroup_I | Subgroup_II |
| DGCR8 | Subgroup_I |  |
| FLJ20551 | Subgroup_I | Subgroup_II |
| TPM3 | Subgroup_I |  |
| TTC15 | Subgroup_I |  |
| RIPK1 | Subgroup_I |  |
| RHBDD1 | Subgroup_I | Subgroup_II |
| ITPKB | Subgroup_I |  |
| UBL4A | Subgroup_I | Subgroup_II |
| ISCU | Subgroup_I | Subgroup_II |
| VIPR1 | Subgroup_I | Subgroup_II |
| KIAA2026 | Subgroup_I |  |
| APBA3 | Subgroup_I | Subgroup_II |
| RHBDD2 | Subgroup_I |  |
| PHGDH | Subgroup_I |  |
| CASP10 | Subgroup_I | Subgroup_II |
| IL1RAPL1 | Subgroup_I | Subgroup_II |
| PGAM1 | Subgroup_I |  |
| AIF1 | Subgroup_I | Subgroup_II |
| ZNF342 | Subgroup_I |  |
| MAN2B1 | Subgroup_I |  |
| SULT1A4 | Subgroup_I |  |
| RAC2 | Subgroup_I |  |
| GLT25D1 | Subgroup_I | Subgroup_II |
| COPE | Subgroup_I |  |
| ARPC1B | Subgroup_I |  |
| C3orf54 | Subgroup_I | Subgroup_II |
| PSME4 | Subgroup_I |  |
| ARPC4 | Subgroup_I | Subgroup_II |
| LOC643668 | Subgroup_I |  |
| TSPO | Subgroup_I | Subgroup_II |
| IL7 | Subgroup_I | Subgroup_III |
| RNH1 | Subgroup_I |  |
| PYCARD | Subgroup_I | Subgroup_II |
| MAEA | Subgroup_I |  |
| SETDB1 | Subgroup_I |  |
| C9orf89 | Subgroup_I |  |
| SUPT4H1 | Subgroup_I |  |
| PARP4 | Subgroup_I |  |
| TNFRSF1A | Subgroup_I |  |
| SCAND1 | Subgroup_I | Subgroup_II |
| PTK9L | Subgroup_I |  |
| ERCC1 | Subgroup_I |  |
| RP11-529I10.4 | Subgroup_I |  |
| CXX1 | Subgroup_I |  |
| CLN3 | Subgroup_I |  |
| FLJ90757 | Subgroup_I |  |
| PQLC1 | Subgroup_I | Subgroup_II |
| GPBAR1 | Subgroup_I |  |
| RABAC1 | Subgroup_I |  |
| C17orf62 | Subgroup_I |  |
| PDXK | Subgroup_I |  |
| ADAMTS16 | Subgroup_I | Subgroup_II |
| RRAS | Subgroup_I |  |
| CALM3 | Subgroup_I |  |
| D15Wsu75e | Subgroup_I |  |
| C1orf183 | Subgroup_I |  |
| HIST2H4A | Subgroup_I |  |
| MRPL28 | Subgroup_I |  |
| GUSB | Subgroup_I | Subgroup_II |
| AP3B1 | Subgroup_I |  |
| PIGS | Subgroup_I |  |
| BAP1 | Subgroup_I |  |
| SLC39A3 | Subgroup_I | Subgroup_II |
| MT1A | Subgroup_I | Subgroup_II |
| TBCD | Subgroup_I |  |
| CASP9 | Subgroup_I |  |
| SLC35A4 | Subgroup_I |  |
| NTE | Subgroup_I |  |
| GTF2F1 | Subgroup_I |  |
| IDH3G | Subgroup_I |  |
| KIAA0922 | Subgroup_I |  |
| ENO1 | Subgroup_I | Subgroup_II |
| PSMD1 | Subgroup_I |  |
| ELF4 | Subgroup_I |  |
| FASN | Subgroup_I |  |
| GNG8 | Subgroup_I |  |
| ELA3B | Subgroup_I | Subgroup_II |
| RAB8A | Subgroup_I |  |
| SND1 | Subgroup_I | Subgroup_II |
| TRIM26 | Subgroup_I |  |
| FAM89B | Subgroup_I |  |
| CSNK2B | Subgroup_I | Subgroup_II |
| YWHAZ | Subgroup_I |  |
| CPNE1 | Subgroup_I |  |
| IL15RA | Subgroup_I | Subgroup_II |
| ARHGAP30 | Subgroup_I |  |
| ATP6V1E1 | Subgroup_I | Subgroup_II |
| IL22 | Subgroup_I | Subgroup_II |
| MTMR12 | Subgroup_I |  |
| LOC389541 | Subgroup_I |  |
| CXXC1 | Subgroup_I |  |
| RELB | Subgroup_I |  |
| DPF2 | Subgroup_I |  |
| OAT | Subgroup_I |  |
| ACP6 | Subgroup_I |  |
| NT5DC3 | Subgroup_I |  |
| CPNE5 | Subgroup_I |  |
| ANXA5 | Subgroup_I |  |
| MT2A | Subgroup_I | Subgroup_II |
| ARCN1 | Subgroup_I |  |
| GPI | Subgroup_I | Subgroup_II |
| IL12RB1 | Subgroup_I | Subgroup_II |
| NPLOC4 | Subgroup_I | Subgroup_II |
| SIN3A | Subgroup_I |  |
| PSMC4 | Subgroup_I |  |
| PSMD4 | Subgroup_I |  |
| KRTAP10-10 | Subgroup_I |  |
| AKT1 | Subgroup_I |  |
| NUCB1 | Subgroup_I | Subgroup_II |
| RAB5B | Subgroup_I |  |
| CSK | Subgroup_I |  |
| STIP1 | Subgroup_I |  |
| KCTD2 | Subgroup_I |  |
| JUND | Subgroup_I |  |
| LOC606724 | Subgroup_I |  |
| EFTUD2 | Subgroup_I |  |
| FLJ22318 | Subgroup_I |  |
| MTMR14 | Subgroup_I |  |
| DYNLRB1 | Subgroup_I | Subgroup_II |
| DDX41 | Subgroup_I | Subgroup_II |
| PSMD2 | Subgroup_I |  |
| RALY | Subgroup_I |  |
| CENTA1 | Subgroup_I |  |
| C6orf159 | Subgroup_I |  |
| IL6 | Subgroup_I | Subgroup_III |
| ARMC7 | Subgroup_I |  |
| ZGPAT | Subgroup_I |  |
| IBSP | Subgroup_I |  |
| ARID1A | Subgroup_I |  |
| TGOLN2 | Subgroup_I |  |
| UQCRC1 | Subgroup_I |  |
| FLJ14466 | Subgroup_I |  |
| ARHGEF1 | Subgroup_I |  |
| SCRN1 | Subgroup_I |  |
| FLJ21865 | Subgroup_I | Subgroup_II |
| PSMF1 | Subgroup_I |  |
| ERBB4 | Subgroup_I |  |
| SIAHBP1 | Subgroup_I |  |
| UBE2L3 | Subgroup_I | Subgroup_II |
| IL1F6 | Subgroup_I | Subgroup_II |
| ABCF1 | Subgroup_I |  |
| SF3A2 | Subgroup_I |  |
| CDC42EP4 | Subgroup_I | Subgroup_II |
| KLF13 | Subgroup_I |  |
| ACAA1 | Subgroup_I |  |
| DAXX | Subgroup_I |  |
| MCM5 | Subgroup_I |  |
| TIAF1 | Subgroup_I |  |
| PPP1R11 | Subgroup_I | Subgroup_II |
| DDX42 | Subgroup_I |  |
| FEZ1 | Subgroup_I |  |
| PTOV1 | Subgroup_I |  |
| SLC16A13 | Subgroup_I |  |
| RASSF5 | Subgroup_I |  |
| DHPS | Subgroup_I | Subgroup_II |
| C3orf39 | Subgroup_I |  |
| IL26 | Subgroup_I | Subgroup_II |
| ZNF473 | Subgroup_I |  |
| CYFIP2 | Subgroup_I |  |
| HYPB | Subgroup_I |  |
| SF3B3 | Subgroup_I |  |
| PFKL | Subgroup_I |  |
| ALDOC | Subgroup_I |  |
| CD14 | Subgroup_I | Subgroup_II |
| TOB1 | Subgroup_I |  |
| SPTAN1 | Subgroup_I |  |
| DYNLL2 | Subgroup_I | Subgroup_II |
| BTG2 | Subgroup_I |  |
| IL16 | Subgroup_I | Subgroup_II |
| TTC1 | Subgroup_I | Subgroup_II |
| FXC1 | Subgroup_I | Subgroup_II |
| C14orf173 | Subgroup_I | Subgroup_II |
| VPS39 | Subgroup_I |  |
| DNASE1 | Subgroup_III | Subgroup_II |
| GSTK1 | Subgroup_III | Subgroup_II |
| CASP7 | Subgroup_III | Subgroup_II |
| MMP23B | Subgroup_III | Subgroup_II |
| PSMB4 | Subgroup_III | Subgroup_II |
| TNFRSF21 | Subgroup_III | Subgroup_II |
| CPSF4 | Subgroup_III | Subgroup_II |
| EIF2S3 | Subgroup_III | Subgroup_II |
| GTF2E2 | Subgroup_III | Subgroup_II |
| C1orf35 |  | Subgroup_II |
| PLEKHB1 |  | Subgroup_II |
| SLC25A25 |  | Subgroup_II |
| TEX10 |  | Subgroup_II |
| NAP1L4 |  | Subgroup_II |
| EIF1 |  | Subgroup_II |
| VKORC1 |  | Subgroup_II |
| NGRN |  | Subgroup_II |
| MAP3K4 |  | Subgroup_II |
| CYB5D1 |  | Subgroup_II |
| RGC32 |  | Subgroup_II |
| C11orf2 |  | Subgroup_II |
| BARD1 |  | Subgroup_II |
| NSUN5B |  | Subgroup_II |
| PSMB5 |  | Subgroup_II |
| AIP |  | Subgroup_II |
| HDAC1 |  | Subgroup_II |
| JTB |  | Subgroup_II |
| NHP2L1 |  | Subgroup_II |
| PRSS15 |  | Subgroup_II |
| SLIT1 |  | Subgroup_II |
| PPAP2A |  | Subgroup_II |
| SURF1 |  | Subgroup_II |
| KNTC1 |  | Subgroup_II |
| USP30 |  | Subgroup_II |
| STK25 |  | Subgroup_II |
| S100A4 |  | Subgroup_II |
| UBADC1 |  | Subgroup_II |
| WDR71 |  | Subgroup_II |
| P2RY8 |  | Subgroup_II |
| DGUOK |  | Subgroup_II |
| TRG20 |  | Subgroup_II |
| SEMA4F |  | Subgroup_II |
| WBSCR22 |  | Subgroup_II |
| FLJ25067 |  | Subgroup_II |
| C1orf33 |  | Subgroup_II |
| C9orf111 |  | Subgroup_II |
| ASH2L |  | Subgroup_II |
| GAMT |  | Subgroup_II |
| LOC401431 |  | Subgroup_II |
| RAD9A |  | Subgroup_II |
| ABCF2 |  | Subgroup_II |
| LOC283871 |  | Subgroup_II |
| CLDN15 |  | Subgroup_II |
| WBSCR16 |  | Subgroup_II |
| C6orf64 |  | Subgroup_II |
| PIGG |  | Subgroup_II |
| TBC1D10C |  | Subgroup_II |
| TPM2 |  | Subgroup_II |
| CSNK2A2 |  | Subgroup_II |
| LOC90580 |  | Subgroup_II |
| ZNF275 |  | Subgroup_II |
| ATXN3 |  | Subgroup_II |
| NPM3 |  | Subgroup_II |
| STX1A |  | Subgroup_II |
| C19orf25 |  | Subgroup_II |
| LACRT |  | Subgroup_II |
| TMEM63A |  | Subgroup_II |
| NUDT16L1 |  | Subgroup_II |
| PHF15 |  | Subgroup_II |
| L3MBTL2 |  | Subgroup_II |
| COG2 |  | Subgroup_II |
| ZNF672 |  | Subgroup_II |
| C13orf8 |  | Subgroup_II |
| C3orf37 |  | Subgroup_II |
| RASA3 |  | Subgroup_II |
| NOLA1 |  | Subgroup_II |
| ZNF133 |  | Subgroup_II |
| CNOT10 |  | Subgroup_II |
| EIF2B5 |  | Subgroup_II |
| MYO9A |  | Subgroup_II |
| MRFAP1 |  | Subgroup_II |
| PABPC4 |  | Subgroup_II |
| ADA |  | Subgroup_II |
| WDR46 |  | Subgroup_II |
| SAMM50 |  | Subgroup_II |
| C9orf90 |  | Subgroup_II |
| FLJ13149 |  | Subgroup_II |
| NPAL3 |  | Subgroup_II |
| LRPPRC |  | Subgroup_II |
| SUPV3L1 |  | Subgroup_II |
| ANKRD39 |  | Subgroup_II |
| C20orf4 |  | Subgroup_II |
| ARIH2 |  | Subgroup_II |
| C1orf50 |  | Subgroup_II |
| ARHGEF19 |  | Subgroup_II |
| DDX56 |  | Subgroup_II |
| ARHGEF6 |  | Subgroup_II |
| MGC52498 |  | Subgroup_II |
| PTDSS1 |  | Subgroup_II |
| PMPCA |  | Subgroup_II |
| SPAG7 |  | Subgroup_II |
| TMEM55B |  | Subgroup_II |
| WDR57 |  | Subgroup_II |
| CREB3L2 |  | Subgroup_II |
| ZNF671 |  | Subgroup_II |
| NUDCD3 |  | Subgroup_II |
| EWSR1 |  | Subgroup_II |
| ENO2 |  | Subgroup_II |
| DDX23 |  | Subgroup_II |
| WDR59 |  | Subgroup_II |
| KIAA1191 |  | Subgroup_II |
| GALT |  | Subgroup_II |
| EIF3S7 |  | Subgroup_II |
| PBX3 |  | Subgroup_II |
| IMPDH2 |  | Subgroup_II |
| MED19 |  | Subgroup_II |
| ZNF589 |  | Subgroup_II |
| WDR73 |  | Subgroup_II |
| THYN1 |  | Subgroup_II |
| C2orf29 |  | Subgroup_II |
| WDR58 |  | Subgroup_II |
| ERCC3 |  | Subgroup_II |
| POLDIP3 |  | Subgroup_II |
| RPUSD3 |  | Subgroup_II |
| COX5B |  | Subgroup_II |
| NEU1 |  | Subgroup_II |
| POLE |  | Subgroup_II |
| CYBA |  | Subgroup_II |
| C9orf23 |  | Subgroup_II |
| ACOT11 |  | Subgroup_II |
| ATOX1 |  | Subgroup_II |
| DNASE2 |  | Subgroup_II |
| C1orf66 |  | Subgroup_II |
| C19orf24 |  | Subgroup_II |
| CLCN6 |  | Subgroup_II |
| MLC1SA |  | Subgroup_II |
| PIGL |  | Subgroup_II |
| DAZAP2 |  | Subgroup_II |
| C15orf17 |  | Subgroup_II |
| ATP6V1F |  | Subgroup_II |
| OGG1 |  | Subgroup_II |
| C20orf116 |  | Subgroup_II |
| UCK2 |  | Subgroup_II |
| PSMB3 |  | Subgroup_II |
| AHDC1 |  | Subgroup_II |
| ADAMTS9 |  | Subgroup_II |
| HOXB4 |  | Subgroup_II |
| DBF4 |  | Subgroup_II |
| PRELID1 |  | Subgroup_II |
| HEXB |  | Subgroup_II |
| PSMB2 |  | Subgroup_II |
| PLEKHJ1 |  | Subgroup_II |
| SMARCAL1 |  | Subgroup_II |
| COMT |  | Subgroup_II |
| GNPDA1 |  | Subgroup_II |
| SNX17 |  | Subgroup_II |
| FLJ20625 |  | Subgroup_II |
| COX8A |  | Subgroup_II |
| MT1X |  | Subgroup_II |
| ZNF524 |  | Subgroup_II |
| LINGO4 |  | Subgroup_II |
| AP4E1 |  | Subgroup_II |
| NICN1 |  | Subgroup_II |
| CD33 |  | Subgroup_II |
| NEDD8 |  | Subgroup_II |
| OXA1L |  | Subgroup_II |
| ARSD |  | Subgroup_II |
| TINF2 |  | Subgroup_II |
| CMTM3 |  | Subgroup_II |
| C20orf141 |  | Subgroup_II |
| ACTG1 |  | Subgroup_II |
| BATF |  | Subgroup_II |
| LOXL4 |  | Subgroup_II |
| CTF8 |  | Subgroup_II |
| ZDHHC7 |  | Subgroup_II |
| ITGB4BP |  | Subgroup_II |
| MICA |  | Subgroup_II |
| TEGT |  | Subgroup_II |
| RDM1 |  | Subgroup_II |
| NSMCE1 |  | Subgroup_II |
| MAD1L1 |  | Subgroup_II |
| HINT2 |  | Subgroup_II |
| ECGF1 |  | Subgroup_II |
| ACY1 |  | Subgroup_II |
| DCTN2 |  | Subgroup_II |
| CAPN3 |  | Subgroup_II |
| C1orf77 |  | Subgroup_II |
| ZNF576 |  | Subgroup_II |
| NOC2L |  | Subgroup_II |
| DYM |  | Subgroup_II |
| CYHR1 |  | Subgroup_II |
| C10orf26 |  | Subgroup_II |
| FAM108A2 |  | Subgroup_II |
| CXorf12 |  | Subgroup_II |
| ST3GAL3 |  | Subgroup_II |
| MRPS34 |  | Subgroup_II |
| C14orf124 |  | Subgroup_II |
| LOC441150 |  | Subgroup_II |
| RUNX1 |  | Subgroup_II |
| CECR1 |  | Subgroup_II |
| EFHD2 |  | Subgroup_II |
| CDC37 |  | Subgroup_II |
| NAPB |  | Subgroup_II |
| AMZ2 |  | Subgroup_II |
| POP7 |  | Subgroup_II |
| MPV17 |  | Subgroup_II |
| APOA1BP |  | Subgroup_II |
| ASCC1 |  | Subgroup_II |
| GTF2H4 |  | Subgroup_II |
| GLI3 |  | Subgroup_II |
| FBXL15 |  | Subgroup_II |
| CMTM7 |  | Subgroup_II |
| CHCHD5 |  | Subgroup_II |
| C1orf188 |  | Subgroup_II |
| ATAD3B |  | Subgroup_II |
| CAMK1 |  | Subgroup_II |
| C12orf52 |  | Subgroup_II |
| EMP3 |  | Subgroup_II |
| ZNF169 |  | Subgroup_II |
| RNF121 |  | Subgroup_II |
| C15orf24 |  | Subgroup_II |
| SETD4 |  | Subgroup_II |
| FKBP9 |  | Subgroup_II |
| BCDIN3 |  | Subgroup_II |
| NIPA2 |  | Subgroup_II |
| VPS25 |  | Subgroup_II |
| PTPN1 |  | Subgroup_II |
| AMICA1 |  | Subgroup_II |
| C1orf177 |  | Subgroup_II |
| PLOD3 |  | Subgroup_II |
| CRYL1 |  | Subgroup_II |
| PRDX6 |  | Subgroup_II |
| CCDC12 |  | Subgroup_II |
| USP21 |  | Subgroup_II |
| CSTB |  | Subgroup_II |
| CST3 |  | Subgroup_II |
| FAM109A |  | Subgroup_II |
| APRT |  | Subgroup_II |
| CD274 |  | Subgroup_II |
| EIF2B1 |  | Subgroup_II |
| ADAMTSL5 |  | Subgroup_II |
| PSMB6 |  | Subgroup_II |
| TSPAN17 |  | Subgroup_II |
| GNA15 |  | Subgroup_II |
| HDHD1A |  | Subgroup_II |
| ABHD12 |  | Subgroup_II |
| NISCH |  | Subgroup_II |
| PWP1 |  | Subgroup_II |
| ADAM15 |  | Subgroup_II |
| GSS |  | Subgroup_II |
| FLJ10241 |  | Subgroup_II |
| ASB13 |  | Subgroup_II |
| TUSC1 |  | Subgroup_II |
| REPS1 |  | Subgroup_II |
| POLR2F |  | Subgroup_II |
| LOC51255 |  | Subgroup_II |
| POLR3C |  | Subgroup_II |
| BAX |  | Subgroup_II |
| APBB3 |  | Subgroup_II |
| PEMT |  | Subgroup_II |
| ATP5D |  | Subgroup_II |
| GLYCTK |  | Subgroup_II |
| CORO1B |  | Subgroup_II |
| NCR3 |  | Subgroup_II |
| SLC35D1 |  | Subgroup_II |
| C9orf142 |  | Subgroup_II |
| EXOSC5 |  | Subgroup_II |
| GMPPA |  | Subgroup_II |
| OR51I1 |  | Subgroup_II |
| CCDC6 |  | Subgroup_II |
| NDUFS7 |  | Subgroup_II |
| B3GNT6 |  | Subgroup_II |
| POGK |  | Subgroup_II |
| USP39 |  | Subgroup_II |
| C16orf78 |  | Subgroup_II |
| LLGL1 |  | Subgroup_II |
| CRR9 |  | Subgroup_II |
| EIF2B4 |  | Subgroup_II |
| ECH1 |  | Subgroup_II |
| GNA11 |  | Subgroup_II |
| CLPP |  | Subgroup_II |
| C1orf164 |  | Subgroup_II |
| C17orf49 |  | Subgroup_II |
| TTC7A |  | Subgroup_II |
| C12orf44 |  | Subgroup_II |
| NDUFV1 |  | Subgroup_II |
| SLC35B1 |  | Subgroup_II |
| C1orf144 |  | Subgroup_II |
| PRPF4 |  | Subgroup_II |
| IKBKE |  | Subgroup_II |
| MFSD5 |  | Subgroup_II |
| LMNB2 |  | Subgroup_II |
| TESK1 |  | Subgroup_II |
| ZNF668 |  | Subgroup_II |
| PROSC |  | Subgroup_II |
| MR1 |  | Subgroup_II |
| IFRD2 |  | Subgroup_II |
| SUCLG1 |  | Subgroup_II |
| NDUFA7 |  | Subgroup_II |
| CYBASC3 |  | Subgroup_II |
| PEX11B |  | Subgroup_II |
| POFUT2 |  | Subgroup_II |
| C20orf35 |  | Subgroup_II |
| COPS6 |  | Subgroup_II |
| ERGIC3 |  | Subgroup_II |
| IGFBP6 |  | Subgroup_II |
| MAP2K5 |  | Subgroup_II |
| TMC6 |  | Subgroup_II |
| CD2BP2 |  | Subgroup_II |
| ALKBH3 |  | Subgroup_II |
| LOC113386 |  | Subgroup_II |
| THADA |  | Subgroup_II |
| EXOC7 |  | Subgroup_II |
| NAGPA |  | Subgroup_II |
| ZCCHC17 |  | Subgroup_II |
| SGSH |  | Subgroup_II |
| D2HGDH |  | Subgroup_II |
| ALKBH7 |  | Subgroup_II |
| TOP3B |  | Subgroup_II |
| KCTD15 |  | Subgroup_II |
| ABHD14B |  | Subgroup_II |
| UNQ501 |  | Subgroup_II |
| ORAOV1 |  | Subgroup_II |
| CRSP6 |  | Subgroup_II |
| ZNF364 |  | Subgroup_II |
| U2AF1L2 |  | Subgroup_II |
| SMC1L1 |  | Subgroup_II |
| STUB1 |  | Subgroup_II |
| SPG7 |  | Subgroup_II |
| CACNB3 |  | Subgroup_II |
| CCDC28B |  | Subgroup_II |
| B4GALT7 |  | Subgroup_II |
| VEGFC |  | Subgroup_II |
| FAM20B |  | Subgroup_II |
| TSSC1 |  | Subgroup_II |
| ADCK2 |  | Subgroup_II |
| TBC1D13 |  | Subgroup_II |
| POLR2I |  | Subgroup_II |
| TRPV2 |  | Subgroup_II |
| ATP5G1 |  | Subgroup_II |
| CNFN |  | Subgroup_II |
| C20orf29 |  | Subgroup_II |
| COPS7A |  | Subgroup_II |
| CHI3L2 |  | Subgroup_II |
| ACSS1 |  | Subgroup_II |
| NASP |  | Subgroup_II |
| TBL3 |  | Subgroup_II |
| ZNF177 |  | Subgroup_II |
| GART |  | Subgroup_II |
| C10orf76 |  | Subgroup_II |
| C9orf114 |  | Subgroup_II |
| CHFR |  | Subgroup_II |
| RBMX |  | Subgroup_II |
| KRTCAP2 |  | Subgroup_II |
| TRMT1 |  | Subgroup_II |
| DOM3Z |  | Subgroup_II |
| M6PR |  | Subgroup_II |
| KARS |  | Subgroup_II |
| SLC7A6OS |  | Subgroup_II |
| CDK5RAP3 |  | Subgroup_II |
| C6orf188 |  | Subgroup_II |
| SPINT2 |  | Subgroup_II |
| CXCR4 |  | Subgroup_II |
| C16orf56 |  | Subgroup_II |
| HSPC117 |  | Subgroup_II |
| NUP188 |  | Subgroup_II |
| DAD1 |  | Subgroup_II |
| TMEM41A |  | Subgroup_II |
| RNMT |  | Subgroup_II |
| ST6GALNAC6 |  | Subgroup_II |
| ZNF324 |  | Subgroup_II |
| BANF1 |  | Subgroup_II |
| MLYCD |  | Subgroup_II |
| NUDT9 |  | Subgroup_II |
| C8orf58 |  | Subgroup_II |
| RARSL |  | Subgroup_II |
| DFFA |  | Subgroup_II |
| XAB1 |  | Subgroup_II |
| PRPSAP1 |  | Subgroup_II |
| C1orf86 |  | Subgroup_II |
| C9orf91 |  | Subgroup_II |
| RHOT2 |  | Subgroup_II |
| DVL2 |  | Subgroup_II |
| HSPC196 |  | Subgroup_II |
| FLJ35725 |  | Subgroup_II |
| CD226 |  | Subgroup_II |
| DCTN3 |  | Subgroup_II |
| NDUFA3 |  | Subgroup_II |
| B3GAT3 |  | Subgroup_II |
| ZNF615 |  | Subgroup_II |
| FMO4 |  | Subgroup_II |
| ZNF285A |  | Subgroup_II |
| GPS1 |  | Subgroup_II |
| HDDC3 |  | Subgroup_II |
| FLJ40142 |  | Subgroup_II |
| U2AF1L4 |  | Subgroup_II |
| EIF2B2 |  | Subgroup_II |
| CARKL |  | Subgroup_II |
| TCEA2 |  | Subgroup_II |
| C2orf34 |  | Subgroup_II |
| ZNF42 |  | Subgroup_II |
| XRCC6 |  | Subgroup_II |
| GNPTG |  | Subgroup_II |
| PKD1 |  | Subgroup_II |
| DRG1 |  | Subgroup_II |
| FLJ20232 |  | Subgroup_II |
| CYB561D2 |  | Subgroup_II |
| DSCR3 |  | Subgroup_II |
| TTC17 |  | Subgroup_II |
| ZNF581 |  | Subgroup_II |
| EPHB6 |  | Subgroup_II |
| TPST2 |  | Subgroup_II |
| DUS3L |  | Subgroup_II |
| NDUFA11 |  | Subgroup_II |
| IPO4 |  | Subgroup_II |
| MRPL37 |  | Subgroup_II |
| TCEAL3 |  | Subgroup_II |
| FAM105B |  | Subgroup_II |
| FAM3A |  | Subgroup_II |
| PSMD7 |  | Subgroup_II |
| SLC20A2 |  | Subgroup_II |
| ADIPOR2 |  | Subgroup_II |
| DDX24 |  | Subgroup_II |
| NOD1 |  | Subgroup_II |
| FAM98C |  | Subgroup_II |
| GTF3C5 |  | Subgroup_II |
| TRAF3 |  | Subgroup_II |
| SLC35D2 |  | Subgroup_II |
| HRBL |  | Subgroup_II |
| RPN1 |  | Subgroup_II |
| MIB2 |  | Subgroup_II |
| NIP30 |  | Subgroup_II |
| LEPRE1 |  | Subgroup_II |
| FLAD1 |  | Subgroup_II |
| KIAA1698 |  | Subgroup_II |
| C11orf48 |  | Subgroup_II |
| CDK9 |  | Subgroup_II |
| ICAM2 |  | Subgroup_II |
| SLC25A42 |  | Subgroup_II |
| TMEM15 |  | Subgroup_II |
| CNOT6L |  | Subgroup_II |
| SLC39A1 |  | Subgroup_II |
| TRIM44 |  | Subgroup_II |
| PPAN |  | Subgroup_II |
| GPR92 |  | Subgroup_II |
| BCL2L13 |  | Subgroup_II |
| ANAPC4 |  | Subgroup_II |
| RBM4B |  | Subgroup_II |
| BRF2 |  | Subgroup_II |
| KATNA1 |  | Subgroup_II |
| TUBGCP2 |  | Subgroup_II |
| MICAL2 | Subgroup_I | Subgroup_III |
| SLC1A5 | Subgroup_I | Subgroup_III |
| MOBKL2A | Subgroup_I | Subgroup_III |
| PILRA | Subgroup_I | Subgroup_III |
| CDA | Subgroup_I | Subgroup_III |
| MLLT7 | Subgroup_I | Subgroup_III |
| IQWD1 | Subgroup_I | Subgroup_III |
| PNPLA2 | Subgroup_I | Subgroup_III |
| PINK1 | Subgroup_I | Subgroup_III |
| SHKBP1 | Subgroup_I | Subgroup_III |
| PHOSPHO1 | Subgroup_I | Subgroup_III |
| IGF2BP2 | Subgroup_I | Subgroup_III |
| PADI1 | Subgroup_I | Subgroup_III |
| TLN1 | Subgroup_I | Subgroup_III |
| PRPF8 | Subgroup_I |  |
| IKBKG | Subgroup_I |  |
| CSDA | Subgroup_I | Subgroup_III |
| EIF4EBP2 | Subgroup_I | Subgroup_III |
| CXCL12 | Subgroup_I | Subgroup_III |
| CTSD | Subgroup_I | Subgroup_III |
| NCF1B | Subgroup_I | Subgroup_III |
| SLC25A23 | Subgroup_I |  |
| TMPRSS9 | Subgroup_I | Subgroup_III |
| SLC38A5 | Subgroup_I | Subgroup_III |
| SLC43A2 | Subgroup_I | Subgroup_III |
| GABARAP | Subgroup_II |  |
| FOSL2 | Subgroup_II |  |
| VTI1B | Subgroup_II |  |
| BRWD3 | Subgroup_II |  |
| IQGAP1 | Subgroup_II |  |
| ANXA3 | Subgroup_II |  |
| ACSL1 | Subgroup_II |  |
| FCGR2A | Subgroup_II | Subgroup_I |
| PYGL | Subgroup_II |  |
| TXNIP | Subgroup_II |  |
| CAPZB | Subgroup_II |  |
| Kua-UEV | Subgroup_II |  |
| FAM63A | Subgroup_II |  |
| PSG9 | Subgroup_II |  |
| PRIM2A | Subgroup_II |  |
| PXN | Subgroup_II | Subgroup_III |
| HIST1H2BE | Subgroup_II |  |
| PPBP | Subgroup_II |  |
| CYorf15B | Subgroup_II |  |
| BOK | Subgroup_II | Subgroup_III |
| NALP1 | Subgroup_II |  |
| C14orf92 | Subgroup_II |  |
| PIP3-E | Subgroup_II |  |
| RSAFD1 | Subgroup_II | Subgroup_III |
| WAC | Subgroup_II |  |
| KRT3 | Subgroup_II | Subgroup_III |
| ZFYVE16 | Subgroup_II |  |
| RGS2 | Subgroup_II |  |
| UBE2R2 | Subgroup_II |  |
| FLJ13946 | Subgroup_II |  |
| CCDC52 | Subgroup_II | Subgroup_III |
| INSM1 | Subgroup_II |  |
| IL17 | Subgroup_II | Subgroup_III |
| FLJ41423 | Subgroup_II |  |
| CAMK1G | Subgroup_II | Subgroup_III |
| ZBTB44 | Subgroup_II |  |
| TOP1 | Subgroup_II |  |
| CCDC47 | Subgroup_II |  |
| SNN | Subgroup_II | Subgroup_III |
| PRSS35 | Subgroup_II |  |
| GPR175 | Subgroup_II | Subgroup_III |
| HIST1H2BH | Subgroup_II |  |
| C2 | Subgroup_II | Subgroup_III |
| NHN1 | Subgroup_II | Subgroup_III |
| TSC22D1 | Subgroup_II |  |
| ERVWE1 | Subgroup_II |  |
| SERPINA13 | Subgroup_II | Subgroup_III |
| PSMD11 | Subgroup_II |  |
| PRR6 | Subgroup_II | Subgroup_III |
| THRAP2 | Subgroup_II |  |
| PHF20L1 | Subgroup_II |  |
| SPRYD3 | Subgroup_II | Subgroup_III |
| MID1IP1 | Subgroup_II |  |
| AIM1 | Subgroup_II | Subgroup_III |
| HIST1H2BC | Subgroup_II |  |
| FLJ45445 | Subgroup_II |  |
| RGMA | Subgroup_II | Subgroup_III |
| CBL | Subgroup_II |  |
| LRRK2 | Subgroup_II |  |
| DGAT2 | Subgroup_II | Subgroup_III |
| ETV6 | Subgroup_II |  |
| CLDN14 | Subgroup_II | Subgroup_III |
| IL19 | Subgroup_III | Subgroup_I |
| CD36 | Subgroup_III | Subgroup_I |
| TMBIM1 |  | Subgroup_III |
| RNF166 |  | Subgroup_III |
| SERPINA1 |  | Subgroup_III |
| SEMA6B |  | Subgroup_III |
| TNIP1 |  | Subgroup_III |
| TNFRSF10B |  | Subgroup_III |
| ZNF289 |  | Subgroup_III |
| CAPG |  | Subgroup_III |
| CORO1C |  | Subgroup_III |
| TP53INP2 |  | Subgroup_III |
| METTL9 |  | Subgroup_III |
| C6orf128 |  | Subgroup_III |
| HPSE |  | Subgroup_III |
| RTF1 |  | Subgroup_III |
| QSCN6 |  | Subgroup_III |
| ANKRD33 |  | Subgroup_III |
| HSPA1A |  | Subgroup_III |
| CHP |  | Subgroup_III |
| GSDM1 |  | Subgroup_III |
| SPI1 |  | Subgroup_III |
| AQP12A |  | Subgroup_III |
| STIM1 |  | Subgroup_III |
| CPSF2 |  | Subgroup_III |
| VASP |  | Subgroup_III |
| UBE4B |  | Subgroup_III |
| MBNL3 |  | Subgroup_III |
| NAPRT1 |  | Subgroup_III |
| CTNNAL1 |  | Subgroup_III |
| MYST2 |  | Subgroup_III |
| LOC652968 |  | Subgroup_III |
| FLJ32447 |  | Subgroup_III |
| FXR2 |  | Subgroup_III |
| TPP2 |  | Subgroup_III |
| PHC1 |  | Subgroup_III |
| ST6GALNAC2 |  | Subgroup_III |
| LOC399744 |  | Subgroup_III |
| HBE1 |  | Subgroup_III |
| C5AR1 |  | Subgroup_III |
| GNB2 |  | Subgroup_III |
| C8orf41 |  | Subgroup_III |
| SYMPK |  | Subgroup_III |
| FLJ25143 |  | Subgroup_III |
| CDC42EP2 |  | Subgroup_III |
| POLR2A |  | Subgroup_III |
| GBL |  | Subgroup_III |
| MAPK3 |  | Subgroup_III |
| MYADM |  | Subgroup_III |
| TRPA1 |  | Subgroup_III |
| SIGLEC7 |  | Subgroup_III |
| SLC9A4 |  | Subgroup_III |
| KIAA1446 |  | Subgroup_III |
| SLC25A11 |  | Subgroup_III |
| ELAVL3 |  | Subgroup_III |
| G6PC |  | Subgroup_III |
| JDP2 |  | Subgroup_III |
| ABTB1 |  | Subgroup_III |
| RNF20 |  | Subgroup_III |
| CHST2 |  | Subgroup_III |
| LIMS1 |  | Subgroup_III |
| SLC36A1 |  | Subgroup_III |
| CAMKK2 |  | Subgroup_III |
| ARTN |  | Subgroup_III |
| HCN1 |  | Subgroup_III |
| UBE2V1 |  | Subgroup_III |
| FAM100B |  | Subgroup_III |
| RTN3 |  | Subgroup_III |
| LOC441476 |  | Subgroup_III |
| POR |  | Subgroup_III |
| CAMK2A |  | Subgroup_III |
| ADIPOR1 |  | Subgroup_III |
| OR2AG1 |  | Subgroup_III |
| SH3GL1 |  | Subgroup_III |
| C20orf108 |  | Subgroup_III |
| CSF2RB |  | Subgroup_III |
| HIST1H2BG |  | Subgroup_III |
| CORO1A |  | Subgroup_III |
| ADM2 |  | Subgroup_III |
| NCF4 |  | Subgroup_III |
| EPB41L3 |  | Subgroup_III |
| CLEC14A |  | Subgroup_III |
| PRKCSH |  | Subgroup_III |
| FAM5B |  | Subgroup_III |
| TACC3 |  | Subgroup_III |
| LOC643165 |  | Subgroup_III |
| MKNK2 |  | Subgroup_III |
| AYP1 |  | Subgroup_III |
| OR51S1 |  | Subgroup_III |
| FLJ45337 |  | Subgroup_III |
| MGC18216 |  | Subgroup_III |
| C18orf10 |  | Subgroup_III |
| GALNT2 |  | Subgroup_III |
| HIST1H3F |  | Subgroup_III |
| ZDHHC18 |  | Subgroup_III |
| C10orf28 |  | Subgroup_III |
| RNF44 |  | Subgroup_III |
| HOXB6 |  | Subgroup_III |
| CLEC4E |  | Subgroup_III |
| FAM83F |  | Subgroup_III |
| ADRA2C |  | Subgroup_III |
| HOXC10 |  | Subgroup_III |
| INPP5D |  | Subgroup_III |
| TNFRSF1B |  | Subgroup_III |
| RALBP1 |  | Subgroup_III |
| CHODL |  | Subgroup_III |
| DPYSL5 |  | Subgroup_III |
| RAB5C |  | Subgroup_III |
| PBX2 |  | Subgroup_III |
| BASP1 |  | Subgroup_III |
| RPL29P2 |  | Subgroup_III |
| DEDD2 |  | Subgroup_III |
| TEF |  | Subgroup_III |
| PITPNA |  | Subgroup_III |
| EMR3 |  | Subgroup_III |
| CBS |  | Subgroup_III |
| CBX3 |  | Subgroup_III |
| NR1D1 |  | Subgroup_III |
| TCEB3 |  | Subgroup_III |
| SCYL1 |  | Subgroup_III |
| KIAA1539 |  | Subgroup_III |
| DHTKD1 |  | Subgroup_III |
| AP4S1 |  | Subgroup_III |
| ELL |  | Subgroup_III |
| IQGAP2 |  | Subgroup_III |
| LPIN2 |  | Subgroup_III |
| C21orf57 |  | Subgroup_III |
| LRP10 |  | Subgroup_III |
| C17orf27 |  | Subgroup_III |
| MAPK15 |  | Subgroup_III |
| ITPK1 |  | Subgroup_III |
| BMP2K |  | Subgroup_III |
| AFTPH |  | Subgroup_III |
| C9orf10OS |  | Subgroup_III |
| CCDC51 |  | Subgroup_III |
| EIF5 |  | Subgroup_III |
| SULF2 |  | Subgroup_III |
| NCF1C |  | Subgroup_III |
| G6PD |  | Subgroup_III |
| IGFBP1 |  | Subgroup_III |
| S100A8 |  | Subgroup_III |
| TBC1D10B |  | Subgroup_III |
| C17orf38 |  | Subgroup_III |
| CXCL14 |  | Subgroup_III |
| PANX2 |  | Subgroup_III |
| PHLDB1 |  | Subgroup_III |
| PTPRN2 |  | Subgroup_III |
| MGC4093 |  | Subgroup_III |
| MYLK |  | Subgroup_III |
| DMRTB1 |  | Subgroup_III |
| DEF6 |  | Subgroup_III |
| ARAF |  | Subgroup_III |
| NOL10 |  | Subgroup_III |
| BCL9 |  | Subgroup_III |
| MANSC1 |  | Subgroup_III |
| WWP2 |  | Subgroup_III |
| LOC440313 |  | Subgroup_III |
| MGC33692 |  | Subgroup_III |
| TRIP12 |  | Subgroup_III |
| ZFYVE1 |  | Subgroup_III |
| TTC7B |  | Subgroup_III |
| PRAM1 |  | Subgroup_III |
| SP1 |  | Subgroup_III |
| SS18 |  | Subgroup_III |
| ERF |  | Subgroup_III |
| DYSFIP1 |  | Subgroup_III |
| BECN1 |  | Subgroup_III |
| RAPGEFL1 |  | Subgroup_III |
| GNA14 |  | Subgroup_III |
| LOC81558 |  | Subgroup_III |
| SLA |  | Subgroup_III |
| TNFAIP6 |  | Subgroup_III |
| WDR26 |  | Subgroup_III |
| MYO5A |  | Subgroup_III |
| ADAMTS4 |  | Subgroup_III |
| UBE2H |  | Subgroup_III |
| ACO2 |  | Subgroup_III |
| CTDP1 |  | Subgroup_III |
| MAGED4 |  | Subgroup_III |
| ZHX2 |  | Subgroup_III |
| TFE3 |  | Subgroup_III |
| GSR |  | Subgroup_III |
| FAM49A |  | Subgroup_III |
| ARHGEF11 |  | Subgroup_III |
| RHCE |  | Subgroup_III |
| MYH4 |  | Subgroup_III |
| DNAJC13 |  | Subgroup_III |
| JAK2 |  | Subgroup_I |
| APOBEC3C |  | Subgroup_III |
| YPEL3 |  | Subgroup_III |
| C20orf3 |  | Subgroup_III |
| GDI1 |  | Subgroup_III |
| COTL1 |  | Subgroup_III |
| DNTTIP1 |  | Subgroup_III |
| PRKD2 |  | Subgroup_III |
| FSCN1 |  | Subgroup_III |
| ARF1 |  | Subgroup_III |
| ERGIC1 |  | Subgroup_III |
| RHPN1 |  | Subgroup_III |
| HIST1H1C |  | Subgroup_III |
| F13A1 |  | Subgroup_III |
| ABHD5 |  | Subgroup_III |
| CAPN1 |  | Subgroup_III |
| OSBPL2 |  | Subgroup_III |
| TMCC3 |  | Subgroup_III |
| PTTG2 |  | Subgroup_III |
| ARF3 |  | Subgroup_III |
| SALL3 |  | Subgroup_III |
| SLC23A1 |  | Subgroup_III |
| MGC26885 |  | Subgroup_III |
| TOB2 |  | Subgroup_III |
| KRTAP19-6 |  | Subgroup_III |
| ROPN1B |  | Subgroup_III |
| TRIM49 |  | Subgroup_III |
| AARSL | Subgroup_I | Subgroup_II |
| TGIF2 | Subgroup_I | Subgroup_II |
| PPP2R2B | Subgroup_II |  |
| PADI3 | Subgroup_II | Subgroup_I |
| IL11 | Subgroup_II | Subgroup_I |
| RPL9 | Subgroup_II | Subgroup_I |
| PRKAB2 | Subgroup_III |  |
| RPL35 | Subgroup_III | Subgroup_I |
| SEC61G | Subgroup_III | Subgroup_I |
| RPS3A |  | Subgroup_I |
| YES1 |  | Subgroup_I |
| TMEM66 |  | Subgroup_I |
| SNX16 |  | Subgroup_I |
| ZNF684 |  | Subgroup_I |
| SAR1B |  | Subgroup_I |
| TFB2M |  | Subgroup_I |
| ZBED5 |  | Subgroup_I |
| RPL31 |  | Subgroup_I |
| ZNF558 |  | Subgroup_I |
| UBE2E1 |  | Subgroup_I |
| RPL23A |  | Subgroup_I |
| TXNL2 |  | Subgroup_I |
| STX8 |  | Subgroup_I |
| P2RY10 |  | Subgroup_I |
| KLRD1 |  | Subgroup_I |
| TIMM9 |  | Subgroup_I |
| WDFY1 |  | Subgroup_I |
| SNAPC5 |  | Subgroup_I |
| RPS15A |  | Subgroup_I |
| ZNF232 |  | Subgroup_I |
| CLEC2D |  | Subgroup_I |
| SRP19 |  | Subgroup_I |
| THOC1 |  | Subgroup_I |
| RPL11 |  | Subgroup_I |
| EEF1G |  | Subgroup_I |
| SEC61B |  | Subgroup_I |
| ACBD3 |  | Subgroup_I |
| RPL13 |  | Subgroup_I |
| MGC12966 |  | Subgroup_I |
| ZWILCH |  | Subgroup_I |
| CRYZ |  | Subgroup_I |
| ARL5A |  | Subgroup_I |
| FLJ20534 |  | Subgroup_I |
| FLJ30046 |  | Subgroup_I |
| ANKRD12 |  | Subgroup_I |
| DPY19L4 |  | Subgroup_I |
| C7orf36 |  | Subgroup_I |
| AP1S2 |  | Subgroup_I |
| DCAL1 |  | Subgroup_I |
| ENOSF1 |  | Subgroup_I |
| SEC63 |  | Subgroup_I |
| LOC492311 |  | Subgroup_I |
| SLC30A9 |  | Subgroup_I |
| C10orf57 |  | Subgroup_I |
| PTPRCAP |  | Subgroup_I |
| AGL |  | Subgroup_I |
| CEBPZ |  | Subgroup_I |
| AFF3 |  | Subgroup_I |
| FLJ16231 |  | Subgroup_I |
| EAF2 |  | Subgroup_I |
| PRNP |  | Subgroup_I |
| SLC30A5 |  | Subgroup_I |
| MASTL |  | Subgroup_I |
| FLJ22555 |  | Subgroup_I |
| PPIL3 |  | Subgroup_I |
| C14orf156 |  | Subgroup_I |
| RRAS2 |  | Subgroup_I |
| LRIG1 |  | Subgroup_I |
| OXSM |  | Subgroup_I |
| MESDC1 |  | Subgroup_I |
| CCDC16 |  | Subgroup_I |
| ITGB3BP |  | Subgroup_I |
| RPS24 |  | Subgroup_I |
| ANKAR |  | Subgroup_I |
| REV1L |  | Subgroup_I |
| FAM36A |  | Subgroup_I |
| CCDC65 |  | Subgroup_I |
| KIAA0907 |  | Subgroup_I |
| BCNP1 |  | Subgroup_I |
| LSM3 |  | Subgroup_I |
| STRBP |  | Subgroup_I |
| MTSS1 |  | Subgroup_I |
| DCUN1D5 |  | Subgroup_I |
| VBP1 |  | Subgroup_I |
| DMTF1 |  | Subgroup_I |
| ENY2 |  | Subgroup_I |
| PIGA |  | Subgroup_I |
| C6orf190 |  | Subgroup_I |
| APPBP2 |  | Subgroup_I |
| ARL5 |  | Subgroup_I |
| BMPR2 |  | Subgroup_I |
| MGC61571 |  | Subgroup_I |
| C20orf155 |  | Subgroup_I |
| MGC2747 |  | Subgroup_I |
| RAI1 |  | Subgroup_I |
| PNRC2 |  | Subgroup_I |
| ABCB7 |  | Subgroup_I |
| NME7 |  | Subgroup_I |
| CTDSPL2 |  | Subgroup_I |
| RPL26 |  | Subgroup_I |
| NCBP2 |  | Subgroup_I |
| C2orf26 |  | Subgroup_I |
| LOC56902 |  | Subgroup_I |
| HNRPDL |  | Subgroup_I |
| C14orf2 |  | Subgroup_I |
| PAQR3 |  | Subgroup_I |
| MAP4K3 |  | Subgroup_I |
| C8orf38 |  | Subgroup_I |
| LOC391356 |  | Subgroup_I |
| FLJ20364 |  | Subgroup_I |
| FLJ16542 |  | Subgroup_I |
| LOC129531 |  | Subgroup_I |
| RAD51AP1 |  | Subgroup_I |
| FLJ21986 |  | Subgroup_I |
| ARV1 |  | Subgroup_I |
| MAN1A2 |  | Subgroup_I |
| C20orf44 |  | Subgroup_I |
| LIN9 |  | Subgroup_I |
| CRI2 |  | Subgroup_I |
| ZZZ3 |  | Subgroup_I |
| TXNDC10 |  | Subgroup_I |
| PAG1 |  | Subgroup_I |
| ZNHIT2 |  | Subgroup_I |
| C12orf47 |  | Subgroup_I |
| CBWD5 |  | Subgroup_I |
| CHCHD7 |  | Subgroup_I |
| ATP2C1 |  | Subgroup_I |
| RAFTLIN |  | Subgroup_I |
| NARG2 |  | Subgroup_I |
| GFM1 |  | Subgroup_I |
| SEPW1 |  | Subgroup_I |
| PVRL3 |  | Subgroup_I |
| ZNF518 |  | Subgroup_I |
| P2RY14 |  | Subgroup_I |
| MKI67IP |  | Subgroup_I |
| FLJ22624 |  | Subgroup_I |
| ABCB10 |  | Subgroup_I |
| DDX26B |  | Subgroup_I |
| RABGGTB |  | Subgroup_I |
| FLJ20272 |  | Subgroup_I |
| ZNF204 |  | Subgroup_I |
| METTL2A |  | Subgroup_I |
| CYP2U1 |  | Subgroup_I |
| C10orf86 |  | Subgroup_I |
| BOLA2 |  | Subgroup_I |
| DHX15 |  | Subgroup_I |
| MAF |  | Subgroup_I |
| PRMT6 |  | Subgroup_I |
| MRPL45 |  | Subgroup_I |
| CCDC53 |  | Subgroup_I |
| DUS4L |  | Subgroup_I |
| PMPCB |  | Subgroup_I |
| LZTFL1 |  | Subgroup_I |
| RAP2A |  | Subgroup_I |
| KIAA0528 |  | Subgroup_I |
| PDGFRA |  | Subgroup_I |
| NCBP1 |  | Subgroup_I |
| COPS4 |  | Subgroup_I |
| LOC286076 |  | Subgroup_I |
| CRBN |  | Subgroup_I |
| CKS2 |  | Subgroup_I |
| CRH |  | Subgroup_I |
| C6orf49 |  | Subgroup_I |
| APPBP1 |  | Subgroup_I |
| EID3 |  | Subgroup_I |
| ACADM |  | Subgroup_I |
| BET1 |  | Subgroup_I |
| HDDC2 |  | Subgroup_I |
| PDHX |  | Subgroup_I |
| RTN4R |  | Subgroup_I |
| KBTBD8 |  | Subgroup_I |
| IMPAD1 |  | Subgroup_I |
| ACTR6 |  | Subgroup_I |
| CHURC1 |  | Subgroup_I |
| FLJ10154 |  | Subgroup_I |
| FAM96A |  | Subgroup_I |
| TWSG1 |  | Subgroup_I |
| RFC3 |  | Subgroup_I |
| LRRC1 |  | Subgroup_I |
| TXNDC |  | Subgroup_I |
| SMARCE1 |  | Subgroup_I |
| MRPL50 |  | Subgroup_I |
| NR2C1 |  | Subgroup_I |
| HSPD1 |  | Subgroup_I |
| ITGB1 |  | Subgroup_I |
| FANCF |  | Subgroup_I |
| MTAC2D1 |  | Subgroup_I |
| NAT1 |  | Subgroup_I |
| SQLE |  | Subgroup_I |
| TMEM108 |  | Subgroup_I |
| FUBP3 |  | Subgroup_I |
| C17orf81 |  | Subgroup_I |
| RPS6KB1 |  | Subgroup_I |
| CIAPIN1 |  | Subgroup_I |
| FLJ32549 |  | Subgroup_I |
| RPL39 |  | Subgroup_I |
| GTF3C3 |  | Subgroup_I |
| C10orf22 |  | Subgroup_I |
| C10orf104 |  | Subgroup_I |
| REEP5 |  | Subgroup_I |
| SH2D1A |  | Subgroup_I |
| C15orf15 |  | Subgroup_I |
| SPRED1 |  | Subgroup_I |
| LRRC37B |  | Subgroup_I |
| RFP2 |  | Subgroup_I |
| CGRRF1 |  | Subgroup_I |
| C14orf142 |  | Subgroup_I |
| PIGY |  | Subgroup_I |
| ABCE1 |  | Subgroup_I |
| PHB2 |  | Subgroup_I |
| FAM76B |  | Subgroup_I |
| C9orf5 |  | Subgroup_I |
| LOC93081 |  | Subgroup_I |
| PANX1 |  | Subgroup_I |
| PQLC3 |  | Subgroup_I |
| IKBKAP |  | Subgroup_I |
| CHD9 |  | Subgroup_I |
| SCOC |  | Subgroup_I |
| TCEB1 |  | Subgroup_I |
| C6orf75 |  | Subgroup_I |
| LACTB2 |  | Subgroup_I |
| APOBEC3G |  | Subgroup_I |
| CBFB |  | Subgroup_I |
| C13orf7 |  | Subgroup_I |
| VPS36 |  | Subgroup_I |
| C9orf21 |  | Subgroup_I |
| IMPA1 |  | Subgroup_I |
| MTHFD2 |  | Subgroup_I |
| LANCL1 |  | Subgroup_I |
| NSUN2 |  | Subgroup_I |
| LOC643940 |  | Subgroup_I |
| MANEA |  | Subgroup_I |
| CCNC |  | Subgroup_I |
| COIL |  | Subgroup_I |
| MRPL32 |  | Subgroup_I |
| NOL11 |  | Subgroup_I |
| SNX4 |  | Subgroup_I |
| EDG1 |  | Subgroup_I |
| BUB3 |  | Subgroup_I |
| EML4 |  | Subgroup_I |
| PTS |  | Subgroup_I |
| C12orf29 |  | Subgroup_I |
| CCDC41 |  | Subgroup_I |
| PDIK1L |  | Subgroup_I |
| ATP6V1G1 |  | Subgroup_I |
| PTPN11 |  | Subgroup_I |
| YRDC |  | Subgroup_I |
| PRPS2 |  | Subgroup_I |
| HRAS |  | Subgroup_I |
| FLJ14213 |  | Subgroup_I |
| ADRB2 |  | Subgroup_I |
| KCTD18 |  | Subgroup_I |
| KIAA1024 |  | Subgroup_I |
| YTHDF2 |  | Subgroup_I |
| JAKMIP2 |  | Subgroup_I |
| TMEM170 |  | Subgroup_I |
| AKAP11 |  | Subgroup_I |
| METAP2 |  | Subgroup_I |
| MAT2B |  | Subgroup_I |
| METTL5 |  | Subgroup_I |
| EXOSC8 |  | Subgroup_I |
| MRPS18B |  | Subgroup_I |
| CG018 |  | Subgroup_I |
| MTX2 |  | Subgroup_I |
| PFDN5 |  | Subgroup_I |
| ZNF721 |  | Subgroup_I |
| TP53I13 |  | Subgroup_I |
| BRD2 |  | Subgroup_I |
| SUCLG2 |  | Subgroup_I |
| MCOLN2 |  | Subgroup_I |
| KLHL9 |  | Subgroup_I |
| DPM1 |  | Subgroup_I |
| ITGA4 |  | Subgroup_I |
| PSMA2 |  | Subgroup_I |
| MATR3 |  | Subgroup_I |
| SKAP1 |  | Subgroup_I |
| NFX1 |  | Subgroup_I |
| LTV1 |  | Subgroup_I |
| ARMCX5 |  | Subgroup_I |
| UBE2Q2 |  | Subgroup_I |
| PAPD4 |  | Subgroup_I |
| FBXO4 |  | Subgroup_I |
| ZNF680 |  | Subgroup_I |
| ZNF627 |  | Subgroup_I |
| HSA9761 |  | Subgroup_I |
| HSP90AA1 |  | Subgroup_I |
| LEPROTL1 |  | Subgroup_I |
| CCDC66 |  | Subgroup_I |
| USP47 |  | Subgroup_I |
| MAP3K7 |  | Subgroup_I |
| RPS20 |  | Subgroup_I |
| NDUFA4 |  | Subgroup_I |
| LOC139886 |  | Subgroup_I |
| KIAA1737 |  | Subgroup_I |
| LINS1 |  | Subgroup_I |
| C3orf64 |  | Subgroup_I |
| MRPL13 |  | Subgroup_I |
| COMMD6 |  | Subgroup_I |
| REV1 |  | Subgroup_I |
| MRS2L |  | Subgroup_I |
| TBC1D4 |  | Subgroup_I |
| HMGN4 |  | Subgroup_I |
| GTF2F2 |  | Subgroup_I |
| ATP5O |  | Subgroup_I |
| RAB10 |  | Subgroup_I |
| TMEM126B |  | Subgroup_I |
| TMED10 |  | Subgroup_I |
| SPIB |  | Subgroup_I |
| MGAT2 |  | Subgroup_I |
| C3orf58 |  | Subgroup_I |
| ZNF265 |  | Subgroup_I |
| COMMD2 |  | Subgroup_I |
| RPS19 |  | Subgroup_I |
| NGDN |  | Subgroup_I |
| DR1 |  | Subgroup_I |
| ZNF83 |  | Subgroup_I |
| MTA1 |  | Subgroup_I |
| LOC144363 |  | Subgroup_I |
| CGI-115 |  | Subgroup_I |
| CCDC4 |  | Subgroup_I |
| MRPL1 |  | Subgroup_I |
| ILDR1 |  | Subgroup_I |
| FAM111A |  | Subgroup_I |
| NOL5A |  | Subgroup_I |
| RY1 |  | Subgroup_I |
| XPO1 |  | Subgroup_I |
| PURA |  | Subgroup_I |
| NFATC2IP |  | Subgroup_I |
| WNT10B |  | Subgroup_I |
| ENTH |  | Subgroup_I |
| CEP152 |  | Subgroup_I |
| YEATS4 |  | Subgroup_I |
| C1orf121 |  | Subgroup_I |
| CD47 |  | Subgroup_I |
| KCTD6 |  | Subgroup_I |
| SUPT3H |  | Subgroup_I |
| ATP5F1 |  | Subgroup_I |
| C14orf104 |  | Subgroup_I |
| PHYH |  | Subgroup_I |
| CMKOR1 |  | Subgroup_I |
| GMFB |  | Subgroup_I |
| LOC344405 |  | Subgroup_I |
| RECK |  | Subgroup_I |
| C16orf63 |  | Subgroup_I |
| ADAM28 |  | Subgroup_I |
| RHEB |  | Subgroup_I |
| MTRR |  | Subgroup_I |
| IL8 |  | Subgroup_I |
| NOC3L |  | Subgroup_I |
| ANAPC10 |  | Subgroup_I |
| ANKRD49 |  | Subgroup_I |
| KDELC2 |  | Subgroup_I |
| AEBP2 |  | Subgroup_I |
| FLJ22104 |  | Subgroup_I |
| ZNF234 |  | Subgroup_I |
| FLJ14803 |  | Subgroup_I |
| CELSR3 |  | Subgroup_I |
| FDX1 |  | Subgroup_I |
| MAP4K5 |  | Subgroup_I |
| LOC91137 |  | Subgroup_I |
| CCDC90B |  | Subgroup_I |
| ZNF439 |  | Subgroup_I |
| PDCD1 |  | Subgroup_I |
| PLCB1 |  | Subgroup_I |
| ATP5L |  | Subgroup_I |
| FAM18B |  | Subgroup_I |
| CUL5 |  | Subgroup_I |
| C10orf97 |  | Subgroup_I |
| MBNL2 |  | Subgroup_I |
| C6orf139 |  | Subgroup_I |
| LYSMD2 |  | Subgroup_I |
| CHEK2 |  | Subgroup_I |
| MIF |  | Subgroup_I |
| ZF |  | Subgroup_I |
| SMEK2 |  | Subgroup_I |
| STT3B |  | Subgroup_I |
| ANKRA2 |  | Subgroup_I |
| C9orf82 |  | Subgroup_I |
| NMD3 |  | Subgroup_I |
| HNRPD |  | Subgroup_I |
| LYAR |  | Subgroup_I |
| TGFBR2 |  | Subgroup_I |
| VPS29 |  | Subgroup_I |
| EI24 |  | Subgroup_I |
| SNHG5 |  | Subgroup_I |
| ARL6IP5 |  | Subgroup_I |
| KIAA1333 |  | Subgroup_I |
| ZNF559 |  | Subgroup_I |
| MUTED |  | Subgroup_I |
| ZNF195 |  | Subgroup_I |
| GTPBP8 |  | Subgroup_I |
| PRKACB |  | Subgroup_I |
| DSCR1L2 |  | Subgroup_I |
| SUHW4 |  | Subgroup_I |
| PSME2 |  | Subgroup_I |
| RGS1 |  | Subgroup_I |
| BRP44L |  | Subgroup_I |
| PPP1CC |  | Subgroup_I |
| TRIM68 |  | Subgroup_I |
| OSGEPL1 |  | Subgroup_I |
| ZNF532 |  | Subgroup_I |
| DSCR2 |  | Subgroup_I |
| PIGH |  | Subgroup_I |
| CCDC59 |  | Subgroup_I |
| MRPS35 |  | Subgroup_I |
| ERO1LB |  | Subgroup_I |
| SC5DL |  | Subgroup_I |
| ANKRD10 |  | Subgroup_I |
| TTC16 |  | Subgroup_I |
| EEF1B2 |  | Subgroup_I |
| NUP54 |  | Subgroup_I |
| UBE1DC1 |  | Subgroup_I |
| GOLGA8B |  | Subgroup_I |
| VDP |  | Subgroup_I |
| LOC90826 |  | Subgroup_I |
| SLC39A10 |  | Subgroup_I |
| SRP72 |  | Subgroup_I |
| SRP9 |  | Subgroup_I |
| RIC3 |  | Subgroup_I |
| RAB11FIP2 |  | Subgroup_I |
| CCDC76 |  | Subgroup_I |
| NFE2L3 |  | Subgroup_I |
| EFHA1 |  | Subgroup_I |
| ERH |  | Subgroup_I |
| AHNAK |  | Subgroup_I |
| ITGAE |  | Subgroup_I |
| DNAJC10 |  | Subgroup_I |
| ZNF548 |  | Subgroup_I |
| LYPLAL1 |  | Subgroup_I |
| SNRPD1 |  | Subgroup_I |
| EXOSC3 |  | Subgroup_I |
| SEPHS1 |  | Subgroup_I |
| TDRD3 |  | Subgroup_I |
| RPL36AL |  | Subgroup_I |
| ZFAND1 |  | Subgroup_I |
| CCDC55 |  | Subgroup_I |
| RNF138 |  | Subgroup_I |
| ZNF383 |  | Subgroup_I |
| TADA1L |  | Subgroup_I |
| SSBP1 |  | Subgroup_I |
| CPSF3 |  | Subgroup_I |
| PIGK |  | Subgroup_I |
| MBIP |  | Subgroup_I |
| FLJ20628 |  | Subgroup_I |
| RNF170 |  | Subgroup_I |
| RTN4IP1 |  | Subgroup_I |
| ZBTB33 |  | Subgroup_I |
| TMED2 |  | Subgroup_I |
| GPAM |  | Subgroup_I |
| ZNF96 |  | Subgroup_I |
| TMED10P |  | Subgroup_I |
| HSPC152 |  | Subgroup_I |
| KIAA0961 |  | Subgroup_I |
| PCGF6 |  | Subgroup_I |
| MRPS28 |  | Subgroup_I |
| SUCLA2 |  | Subgroup_I |
| SUB1 |  | Subgroup_I |
| SLC4A7 |  | Subgroup_I |
| ZNF511 |  | Subgroup_I |
| USPL1 |  | Subgroup_I |
| TIMM8A |  | Subgroup_I |
| THUMPD1 |  | Subgroup_I |
| TM2D1 |  | Subgroup_I |
| ZCCHC9 |  | Subgroup_I |
| RPS6 |  | Subgroup_I |
| TMEM106C |  | Subgroup_I |
| ZNF248 |  | Subgroup_I |
| TCEAL8 |  | Subgroup_I |
| KIAA1411 |  | Subgroup_I |
| TRAF5 |  | Subgroup_I |
| DHX36 |  | Subgroup_I |
| RPLP1 |  | Subgroup_I |
| MRPL16 |  | Subgroup_I |
| RPL5 |  | Subgroup_I |
| GRPEL2 |  | Subgroup_I |
| ITGB1BP1 |  | Subgroup_I |
| MRPL18 |  | Subgroup_I |
| KIAA1287 |  | Subgroup_I |
| SIRT1 |  | Subgroup_I |
| WIPI2 | Subgroup_I |  |
| SLC25A37 | Subgroup_I |  |
| NP | Subgroup_I |  |
| SDCCAG1 | Subgroup_I |  |
| SERTAD1 | Subgroup_I |  |
| YY1AP1 | Subgroup_I |  |
| SRPR | Subgroup_I |  |
| TRAPPC5 | Subgroup_I |  |
| SEC14L1 | Subgroup_I |  |
| RIPK4 | Subgroup_I |  |
| ZNF692 | Subgroup_I |  |
| SF3B2 | Subgroup_I |  |
| TESC | Subgroup_I |  |
| HDC | Subgroup_I |  |
| TAGLN2 | Subgroup_I |  |
| VAT1 | Subgroup_I |  |
| TKT | Subgroup_I |  |
| TAX1BP3 | Subgroup_I |  |
| TM7SF2 | Subgroup_I |  |
| UBE1 | Subgroup_I |  |
| ST6GALNAC4 | Subgroup_I |  |
| TUBA1A | Subgroup_I |  |
| RHOG | Subgroup_I |  |
| NEIL2 | Subgroup_I |  |
| ST3GAL2 | Subgroup_I |  |
| CHD2 | Subgroup_I |  |
| WWC3 | Subgroup_I |  |
| SYNJ2 | Subgroup_I |  |
| TNFSF9 | Subgroup_I |  |
| STXBP2 | Subgroup_I |  |
| UBAP2L | Subgroup_I |  |
| SMOX | Subgroup_I |  |
| SNCA | Subgroup_I |  |
| C3orf60 | Subgroup_I |  |
| SNAPC2 | Subgroup_I |  |
| EIF3S9 | Subgroup_I |  |
| MAGEH1 | Subgroup_I |  |
| LOC283174 | Subgroup_I |  |
| WDR40A | Subgroup_I |  |
| VPS28 | Subgroup_I |  |
| SUV39H2 | Subgroup_I |  |
| TSPAN1 | Subgroup_I |  |
| MGC10993 | Subgroup_I |  |
| VWCE | Subgroup_I |  |
| SAMD11 | Subgroup_I |  |
| ENPP1 | Subgroup_I |  |
| SH3KBP1 | Subgroup_I |  |
| SLC6A12 | Subgroup_I |  |
| TIGD6 | Subgroup_I |  |
| RNPEP | Subgroup_I |  |
| SH3GLB2 | Subgroup_I |  |
| FURIN | Subgroup_I |  |
| C5 | Subgroup_I |  |
| Ells1 | Subgroup_I |  |
| ALOX12 | Subgroup_I |  |
| PRDX5 | Subgroup_I |  |
| C6orf69 | Subgroup_I |  |
| ARL4 | Subgroup_I |  |
| TSTA3 | Subgroup_I |  |
| ATP6AP1 | Subgroup_I |  |
| AP2A1 | Subgroup_I |  |
| ITLN1 | Subgroup_I |  |
| SIAH2 | Subgroup_I |  |
| ALDH3B1 | Subgroup_I |  |
| C6orf21 | Subgroup_I |  |
| CTTN | Subgroup_I |  |
| HGD | Subgroup_I |  |
| MAP1A | Subgroup_I |  |
| BSCL2 | Subgroup_I |  |
| GPR84 | Subgroup_I |  |
| PCBP2 | Subgroup_I |  |
| CML2 | Subgroup_I |  |
| MMRN1 | Subgroup_I |  |
| CDIPT | Subgroup_I |  |
| NOMO1 | Subgroup_I |  |
| CCBP2 | Subgroup_I |  |
| PKM2 | Subgroup_I |  |
| AGTR2 | Subgroup_I |  |
| GFRA2 | Subgroup_I |  |
| SRF | Subgroup_I |  |
| HDGF | Subgroup_I |  |
| H2BFS | Subgroup_I |  |
| GCAT | Subgroup_I |  |
| LOC128439 | Subgroup_I |  |
| MIR16 | Subgroup_I |  |
| F8A1 | Subgroup_I |  |
| NGFB | Subgroup_I |  |
| SREBF2 | Subgroup_I |  |
| IMPA2 | Subgroup_I |  |
| GPX1 | Subgroup_I |  |
| BTBD12 | Subgroup_I |  |
| YPEL4 | Subgroup_I |  |
| XK | Subgroup_I |  |
| EPN1 | Subgroup_I |  |
| HMG20B | Subgroup_I |  |
| FAM104A | Subgroup_I |  |
| FECH | Subgroup_I |  |
| PTGIR | Subgroup_I |  |
| FAM53C | Subgroup_I |  |
| PRUNE | Subgroup_I |  |
| ATF5 | Subgroup_I |  |
| GRK6 | Subgroup_I |  |
| POLL | Subgroup_I |  |
| VENTX | Subgroup_I |  |
| LRRC35 | Subgroup_I |  |
| CUEDC1 | Subgroup_I |  |
| ACSBG1 | Subgroup_I |  |
| CABP5 | Subgroup_I |  |
| LOC401052 | Subgroup_I |  |
| CAPNS1 | Subgroup_I |  |
| BAT5 | Subgroup_I |  |
| DHRS1 | Subgroup_I |  |
| NRK | Subgroup_I |  |
| PTMS | Subgroup_I |  |
| ABCA4 | Subgroup_I |  |
| C1orf198 | Subgroup_I |  |
| OSBP2 | Subgroup_I |  |
| WNK1 | Subgroup_I |  |
| LASP1 | Subgroup_I |  |
| PTPRC | Subgroup_I | Subgroup_II |
| LOC402055 | Subgroup_I |  |
| CXCL13 | Subgroup_I | Subgroup_II |
| HYAL3 | Subgroup_I |  |
| TNNC2 | Subgroup_I |  |
| HADHA | Subgroup_I |  |
| TUBB2C | Subgroup_I |  |
| CLDN5 | Subgroup_I |  |
| UBXD1 | Subgroup_I |  |
| RNF123 | Subgroup_I |  |
| FBP1 | Subgroup_I |  |
| BPGM | Subgroup_I |  |
| ADCY4 | Subgroup_I |  |
| ACTN4 | Subgroup_I |  |
| SH3BGRL3 | Subgroup_I |  |
| SLC24A4 | Subgroup_I |  |
| LILRB3 | Subgroup_I |  |
| LYL1 | Subgroup_I |  |
| GYPB | Subgroup_I |  |
| COX15 | Subgroup_I |  |
| PBX1 | Subgroup_I |  |
| FKBP1A | Subgroup_I |  |
| AP2B1 | Subgroup_I |  |
| VWF | Subgroup_I |  |
| ACTA2 | Subgroup_I |  |
| TMEM127 | Subgroup_I |  |
| TRAFD1 | Subgroup_I |  |
| ATP6V0C | Subgroup_I |  |
| C19orf22 | Subgroup_I |  |
| CMTM5 | Subgroup_I |  |
| MAP2K3 | Subgroup_I |  |
| FLJ22386 | Subgroup_I |  |
| SYTL4 | Subgroup_I |  |
| CCDC23 | Subgroup_I |  |
| XPO7 | Subgroup_I |  |
| LRCH4 | Subgroup_I |  |
| DEFB106B | Subgroup_I |  |
| SHARPIN | Subgroup_I |  |
| MSN | Subgroup_I |  |
| HIST1H3H | Subgroup_I |  |
| RAP1GA1 | Subgroup_I |  |
| ACVR1B | Subgroup_I |  |
| GBGT1 | Subgroup_I |  |
| ATG9A | Subgroup_I |  |
| PDZK1IP1 | Subgroup_I |  |
| PRDX2 | Subgroup_I |  |
| ESAM | Subgroup_I |  |
| C16orf35 | Subgroup_I |  |
| MGLL | Subgroup_I |  |
| SNX27 | Subgroup_I |  |
| MPP1 | Subgroup_I |  |
| C5orf4 | Subgroup_I |  |
| EHD1 | Subgroup_I |  |
| RPIP8 | Subgroup_I |  |
| ZXDC | Subgroup_I |  |
| ZMAT2 | Subgroup_I |  |
| NAPA | Subgroup_I |  |
| MFSD7 | Subgroup_I |  |
| NCOA4 | Subgroup_I |  |
| TMOD1 | Subgroup_I |  |
| MICALCL | Subgroup_I |  |
| SAPS1 | Subgroup_I |  |
| MPL | Subgroup_I |  |
| TRIM10 | Subgroup_I |  |
| LRRC15 | Subgroup_I |  |
| BAT3 | Subgroup_I |  |
| WDR1 | Subgroup_I |  |
| DGCR14 | Subgroup_I |  |
| MYO1A | Subgroup_I |  |
| UROD | Subgroup_I |  |
| GP9 | Subgroup_I |  |
| LATS2 | Subgroup_I |  |
| SSBP3 | Subgroup_I |  |
| GPR108 | Subgroup_I |  |
| HIST1H2AE | Subgroup_I |  |
| GFI1B | Subgroup_I |  |
| GATS | Subgroup_I |  |
| GP1BA | Subgroup_I |  |
| CCS | Subgroup_I |  |
| ARHGAP1 | Subgroup_I |  |
| ALDOA | Subgroup_I |  |
| UBE2O | Subgroup_I |  |
| BRD4 | Subgroup_I |  |
| DENND1A | Subgroup_I |  |
| CCNY | Subgroup_I |  |
| ABCC3 | Subgroup_I |  |
| INTS1 | Subgroup_I |  |
| CGI-69 | Subgroup_I |  |
| MOSPD3 | Subgroup_I |  |
| CARM1 | Subgroup_I |  |
| INPPL1 | Subgroup_I |  |
| EGLN2 | Subgroup_I |  |
| IMPG2 | Subgroup_I |  |
| TNFRSF19 | Subgroup_I | Subgroup_II |
| SIGLEC9 | Subgroup_I |  |
| BDKRB1 | Subgroup_I |  |
| CD82 | Subgroup_I |  |
| NTN2L | Subgroup_I |  |
| UNC45A | Subgroup_I |  |
| CDKN1A | Subgroup_I |  |
| GYPC | Subgroup_I |  |
| SLC30A3 | Subgroup_I |  |
| CTDSPL | Subgroup_I |  |
| ABCC13 | Subgroup_I |  |
| CYB5R3 | Subgroup_I |  |
| RAPGEF1 | Subgroup_I |  |
| GOLGA2 | Subgroup_I |  |
| CRYGA | Subgroup_I |  |
| HMHA1 | Subgroup_I |  |
| GABBR1 | Subgroup_I |  |
| GALNTL1 | Subgroup_I | Subgroup_II |
| CDC34 | Subgroup_I |  |
| MGC15476 | Subgroup_I |  |
| ANKRD9 | Subgroup_I |  |
| NFE2 | Subgroup_I |  |
| MGC3121 | Subgroup_I |  |
| ODZ2 | Subgroup_I |  |
| IL21 | Subgroup_I | Subgroup_III |
| MEF2B | Subgroup_I |  |
| FLJ21839 | Subgroup_I |  |
| E2F2 | Subgroup_I |  |
| SLC6A8 | Subgroup_I |  |
| RDBP | Subgroup_I |  |
| EPB49 | Subgroup_I |  |
| MGC13057 | Subgroup_I |  |
| TCN1 | Subgroup_I |  |
| TSPAN9 | Subgroup_I |  |
| CA1 | Subgroup_I |  |
| TSPAN33 | Subgroup_I |  |
| CLEC1B | Subgroup_I |  |
| FAM77D | Subgroup_I |  |
| PAPD5 | Subgroup_I |  |
| CDK5RAP2 | Subgroup_I |  |
| ARID4B | Subgroup_I | Subgroup_II |
| SLCO4A1 | Subgroup_I |  |
| OSBPL6 | Subgroup_I |  |
| GPR179 | Subgroup_I |  |
| C2orf18 | Subgroup_I |  |
| KIAA0310 | Subgroup_I |  |
| ASCC2 | Subgroup_I |  |
| EIF4G1 | Subgroup_I |  |
| TXNRD2 | Subgroup_I |  |
| SLC7A5 | Subgroup_I |  |
| GYPE | Subgroup_I |  |
| PVALB | Subgroup_I |  |
| ADAMTS19 | Subgroup_I |  |
| PIP5K1C | Subgroup_I |  |
| PHCA | Subgroup_I |  |
| LOC92270 | Subgroup_I |  |
| WIRE | Subgroup_I |  |
| ATP10A | Subgroup_I |  |
| LOC400566 | Subgroup_I |  |
| BCL2L1 | Subgroup_I |  |
| RIOK3 | Subgroup_I |  |
| GSPT1 | Subgroup_I |  |
| LOC651423 | Subgroup_I |  |
| TMEM2 | Subgroup_I |  |
| CRHR1 | Subgroup_I |  |
| AQP10 | Subgroup_I |  |
| CD151 | Subgroup_I |  |
| SESN3 | Subgroup_I |  |
| GPS2 | Subgroup_I |  |
| GATA1 | Subgroup_I |  |
| REXO1 | Subgroup_I |  |
| WASF2 | Subgroup_I |  |
| GALNAC4S-6ST | Subgroup_I |  |
| PTCRA | Subgroup_I |  |
| KLC3 | Subgroup_I |  |
| IFIT1L | Subgroup_I |  |
| UBL7 | Subgroup_I |  |
| SNX26 | Subgroup_I |  |
| HIF1AN | Subgroup_I |  |
| ACRBP | Subgroup_I |  |
| EIF5B | Subgroup_I |  |
| DAP | Subgroup_I |  |
| EXT1 | Subgroup_I |  |
| GRINA | Subgroup_I |  |
| IFI27 | Subgroup_I |  |
| CEACAM21 | Subgroup_I |  |
| BCKDHA | Subgroup_I |  |
| ABCD1 | Subgroup_I |  |
| OR2W5 | Subgroup_I |  |
| RAB3IL1 | Subgroup_I |  |
| C10orf56 | Subgroup_I |  |
| RBPMS2 | Subgroup_I |  |
| NME4 | Subgroup_I |  |
| LRP6 | Subgroup_I |  |
| CASC3 | Subgroup_I |  |
| KEL | Subgroup_I |  |
| STYXL1 | Subgroup_I |  |
| GBF1 | Subgroup_I |  |
| LST1 | Subgroup_I |  |
| CES3 | Subgroup_I |  |
| EIF2AK1 | Subgroup_I |  |
| RBM38 | Subgroup_I |  |
| MMP2 | Subgroup_I | Subgroup_II |
| PIP5K2A | Subgroup_I |  |
| HEMGN | Subgroup_I |  |
| C20orf55 | Subgroup_I |  |
| MKRN1 | Subgroup_I |  |
| GPR146 | Subgroup_I |  |
| FCN1 | Subgroup_I | Subgroup_II |
| TSPAN5 | Subgroup_I |  |
| C11orf30 | Subgroup_I |  |
| SH3BGRL2 | Subgroup_I |  |
| PRKCB1 | Subgroup_I |  |
| DPM2 | Subgroup_I |  |
| FLJ43855 | Subgroup_I |  |
| RGS10 | Subgroup_I |  |
| AP2M1 | Subgroup_I |  |
| KRTHB3 | Subgroup_I |  |
| CITED4 | Subgroup_I |  |
| SCGB1C1 | Subgroup_I |  |
| PAIP1 | Subgroup_I |  |
| LOC349338 | Subgroup_I |  |
| GLRX5 | Subgroup_I |  |
| GATAD2A | Subgroup_I |  |
| DEDD | Subgroup_I |  |
| GALNT8 | Subgroup_I | Subgroup_II |
| SH2D3C | Subgroup_I |  |
| ACTN1 | Subgroup_I |  |
| FGF18 | Subgroup_I |  |
| ELA2B | Subgroup_I | Subgroup_II |
| LHPP | Subgroup_I |  |
| U1SNRNPBP | Subgroup_I |  |
| GMPR | Subgroup_I |  |
| AATF | Subgroup_I |  |
| SAMD14 | Subgroup_I |  |
| GRIN1 | Subgroup_I |  |
| C2orf24 | Subgroup_I |  |
| C20orf67 | Subgroup_I |  |
| KRT1 | Subgroup_I |  |
| IQGAP3 | Subgroup_I |  |
| PIK3R2 | Subgroup_I |  |
| SP2 | Subgroup_I |  |
| LOC407835 | Subgroup_I |  |
| NFIX | Subgroup_I |  |
| BRE | Subgroup_I |  |
| HSPC142 | Subgroup_I |  |
| RDH5 | Subgroup_I |  |
| CDKN2D | Subgroup_I |  |
| PIGQ | Subgroup_I |  |
| ELOF1 | Subgroup_I |  |
| ZYG11BL | Subgroup_I |  |
| LMNA | Subgroup_I |  |
| FAM46C | Subgroup_I |  |
| ACPT | Subgroup_I |  |
| AKAP3 | Subgroup_I |  |
| CTSL | Subgroup_I |  |
| CREBL1 | Subgroup_I |  |
| IL17E | Subgroup_I | Subgroup_III |
| PAPSS2 | Subgroup_I |  |
| PPP2R5B | Subgroup_I |  |
| LRSAM1 | Subgroup_I |  |
| MGC9850 | Subgroup_I |  |
| MCOLN1 | Subgroup_I |  |
| FLJ20297 | Subgroup_I |  |
| GUCA1C | Subgroup_I |  |
| NINJ2 | Subgroup_I |  |
| KCNJ10 | Subgroup_I |  |
| SH3BP1 | Subgroup_I |  |
| CHRNA2 | Subgroup_I |  |
| PIAS4 | Subgroup_I |  |
| MED25 | Subgroup_I |  |
| RANBP10 | Subgroup_I |  |
| C18orf8 | Subgroup_I |  |
| PHKA1 | Subgroup_I |  |
| MAOA | Subgroup_I |  |
| NIBP | Subgroup_I |  |
| TGM2 | Subgroup_I |  |
| PARVB | Subgroup_I |  |
| ALS2CR2 | Subgroup_I |  |
| EPS15L1 | Subgroup_I |  |
| RUFY1 | Subgroup_I |  |
| IRF3 | Subgroup_I |  |
| GLUL | Subgroup_I |  |
| ACTR1A | Subgroup_I |  |
| CRIPAK | Subgroup_I |  |
| LTF | Subgroup_I |  |
| LOC253012 | Subgroup_I |  |
| MAF1 | Subgroup_I |  |
| PPGB | Subgroup_I |  |
| OGFR | Subgroup_I |  |
| ZNF659 | Subgroup_I |  |
| HAGH | Subgroup_I |  |
| DSCAM | Subgroup_I |  |
| KIAA1862 | Subgroup_I |  |
| PSD4 | Subgroup_I |  |
| STK11 | Subgroup_I |  |
| C19orf33 | Subgroup_I |  |
| CNOT3 | Subgroup_I |  |
| GFRA4 | Subgroup_I |  |
| NT5M | Subgroup_I |  |
| HBQ1 | Subgroup_I |  |
| C22orf13 | Subgroup_I |  |
| GPR137 | Subgroup_I |  |
| RAB2B | Subgroup_I |  |
| ATAD4 | Subgroup_I |  |
| URP2 | Subgroup_I |  |
| LOC400120 | Subgroup_I |  |
| TPP1 | Subgroup_I |  |
| OR2W3 | Subgroup_I |  |
| CLPTM1 | Subgroup_I |  |
| C12orf10 | Subgroup_I |  |
| ODC1 | Subgroup_I |  |
| MYOM1 | Subgroup_I |  |
| SLC2A1 | Subgroup_I |  |
| NLRP3 | Subgroup_I |  |
| CARHSP1 | Subgroup_I |  |
| SPARC | Subgroup_I |  |
| LOC285429 | Subgroup_I |  |
| IL31RA | Subgroup_I | Subgroup_II |
| LYK5 | Subgroup_I |  |
| TLR5 | Subgroup_I | Subgroup_II |
| C19orf21 | Subgroup_I |  |
| FLJ90805 | Subgroup_I |  |
| PPME1 | Subgroup_I |  |
| GNAS | Subgroup_I |  |
| KHSRP | Subgroup_I |  |
| PF4V1 | Subgroup_I |  |
| RAB4B | Subgroup_I |  |
| CDH9 | Subgroup_I |  |
| CHMP4B | Subgroup_I |  |
| C2orf17 | Subgroup_I |  |
| PRDM9 | Subgroup_I |  |
| CFHR2 | Subgroup_I |  |
| PLCB2 | Subgroup_I |  |
| SLC22A18AS | Subgroup_I |  |
| CACNA1D | Subgroup_I |  |
| FLJ13910 | Subgroup_I |  |
| FES | Subgroup_I |  |
| ACTRT1 | Subgroup_I |  |
| FLJ20489 | Subgroup_I |  |
| RAD23A | Subgroup_I |  |
| HMG2L1 | Subgroup_I |  |
| ATP1A3 | Subgroup_I |  |
| FNTB | Subgroup_I |  |
| PCDHGB6 | Subgroup_I |  |
| B3GALT1 | Subgroup_I |  |
| C14orf48 | Subgroup_I |  |
| GAS2L3 | Subgroup_I |  |
| HPS1 | Subgroup_I |  |
| WDR13 | Subgroup_I |  |
| PLEK2 | Subgroup_I |  |
| ICAM4 | Subgroup_I |  |
| MICAL1 | Subgroup_I |  |
| SIPA1L3 | Subgroup_I |  |
| FLJ33790 | Subgroup_I |  |
| FCHO1 | Subgroup_I |  |
| GNA12 | Subgroup_I |  |
| LOC653133 | Subgroup_I |  |
| TSPAN6 | Subgroup_I |  |
| PPM1A | Subgroup_I |  |
| MARCKSL1 | Subgroup_I |  |
| IER3 | Subgroup_I |  |
| DARC | Subgroup_I |  |
| PTK6 | Subgroup_I |  |
| FHL2 | Subgroup_I |  |
| TCTA | Subgroup_I |  |
| MGC26733 | Subgroup_I |  |
| EPB42 | Subgroup_I |  |
| LGALS9 | Subgroup_I |  |
| HDAC6 | Subgroup_I |  |
| PRSS7 | Subgroup_I |  |
| TPM1 | Subgroup_I |  |
| FHL1 | Subgroup_I |  |
| FOLH1 | Subgroup_I |  |
| ERAF | Subgroup_I |  |
| CYB5R1 | Subgroup_I |  |
| CDH23 | Subgroup_I |  |
| NGFRAP1 | Subgroup_I |  |
| BPI | Subgroup_I |  |
| DULLARD | Subgroup_I |  |
| EIF2C2 | Subgroup_I |  |
| LRRC28 | Subgroup_I |  |
| UBAP1 | Subgroup_I |  |
| DNAJB2 | Subgroup_I |  |
| LOC653742 | Subgroup_I |  |
| KLF1 | Subgroup_I |  |
| FMO6 | Subgroup_I |  |
| CNNM1 | Subgroup_I |  |
| STAT5A | Subgroup_I |  |
| PRR5 | Subgroup_I |  |
| APCS | Subgroup_I |  |
| CHRNA3 | Subgroup_I |  |
| INPP5F | Subgroup_I |  |
| CECR6 | Subgroup_I |  |
| BAT2 | Subgroup_I |  |
| TMEM40 | Subgroup_I |  |
| WARS | Subgroup_I |  |
| CSNK2A1 | Subgroup_I |  |
| CDC2L2 | Subgroup_I |  |
| STX5A | Subgroup_I |  |
| ZNF341 | Subgroup_I |  |
| LOC284194 | Subgroup_I |  |
| PPP1R9B | Subgroup_I |  |
| SLC4A1 | Subgroup_I |  |
| ATP8A2 | Subgroup_I |  |
| HBZ | Subgroup_I |  |
| DNASE1L1 | Subgroup_I |  |
| IGHMBP2 | Subgroup_I |  |
| ZDHHC5 | Subgroup_I |  |
| NFKB2 | Subgroup_I |  |
| RFNG | Subgroup_I |  |
| CASP5 | Subgroup_I | Subgroup_II |
| KIF14 | Subgroup_I |  |
| FOXO3A | Subgroup_I |  |
| SCML2 | Subgroup_I |  |
| STK33 | Subgroup_I |  |
| TMEM8 | Subgroup_I |  |
| ARHGEF2 | Subgroup_I |  |
| TFDP1 | Subgroup_I |  |
| FIBCD1 | Subgroup_I |  |
| IL17D | Subgroup_I |  |
| SULT6B1 | Subgroup_I |  |
| C22orf25 | Subgroup_I |  |
| ZC3H3 | Subgroup_I |  |
| TXNRD1 | Subgroup_I |  |
| SPRR1B | Subgroup_I |  |
| CXorf43 | Subgroup_I |  |
| EPB41 | Subgroup_I |  |
| GNL3L | Subgroup_I |  |
| CCDC17 | Subgroup_I |  |
| C16orf9 | Subgroup_I |  |
| RAP80 | Subgroup_I |  |
| C1orf128 | Subgroup_I |  |
| HPCAL1 | Subgroup_I |  |
| TTC25 | Subgroup_I |  |
| GJA12 | Subgroup_I |  |
| C8orf34 | Subgroup_I |  |
| DDB1 | Subgroup_I |  |
| CHST3 | Subgroup_I |  |
| FBXO7 | Subgroup_I |  |
| SYK | Subgroup_I |  |
| ZRANB1 | Subgroup_I |  |
| TBC1D16 | Subgroup_I |  |
| RAMP2 | Subgroup_I |  |
| GPC6 | Subgroup_I |  |
| RFXANK | Subgroup_I |  |
| DKFZp434K1815 | Subgroup_I |  |
| LOC115098 | Subgroup_I |  |
| MGC70857 | Subgroup_I |  |
| ANXA8 | Subgroup_I |  |
| IL1B | Subgroup_I | Subgroup_II |
| NSUN3 | Subgroup_I |  |
| HMBS | Subgroup_I |  |
| C6orf25 | Subgroup_I |  |
| MMP15 | Subgroup_I |  |
| WDR45 | Subgroup_I |  |
| HBM | Subgroup_I |  |
| IL1F8 | Subgroup_I | Subgroup_III |
| PIM1 | Subgroup_I |  |
| HGS | Subgroup_I |  |
| UBXD3 | Subgroup_I |  |
| CSPG3 | Subgroup_I |  |
| UCP2 | Subgroup_I |  |
| HOXD3 | Subgroup_I |  |
| GUK1 | Subgroup_I |  |
| TFDP2 | Subgroup_I |  |
| UNC5C | Subgroup_I |  |
| FAM12A | Subgroup_I |  |
| MGC10433 | Subgroup_I |  |
| RILP | Subgroup_I |  |
| FLJ20581 | Subgroup_I |  |
| UNQ846 | Subgroup_I |  |
| GMEB2 | Subgroup_I |  |
| UBQLN3 | Subgroup_I |  |
| MSI2 | Subgroup_I |  |
| ORMDL3 | Subgroup_I |  |
| JAM3 | Subgroup_I |  |
| LRP12 | Subgroup_I |  |
| GLTSCR1 | Subgroup_I |  |
| FARP2 | Subgroup_I |  |
| ACP5 | Subgroup_I |  |
| SCRN2 | Subgroup_I |  |
| ALAS2 | Subgroup_I |  |
| SPATA20 | Subgroup_I |  |
| RIS1 | Subgroup_I |  |
| DNAJA4 | Subgroup_I |  |
| RNF10 | Subgroup_I |  |
| ALDH5A1 | Subgroup_I |  |
| STX10 | Subgroup_I |  |
| KLHL21 | Subgroup_I |  |
| LOC285074 | Subgroup_I |  |
| UBE2M | Subgroup_I |  |
| SMARCC2 | Subgroup_I |  |
| TSSC4 | Subgroup_I |  |
| BRPF3 | Subgroup_I |  |
| PURG | Subgroup_I |  |
| ZNF718 | Subgroup_I |  |
| MXI1 | Subgroup_I |  |
| WDR48 | Subgroup_I |  |
| TBC1D3B | Subgroup_I |  |
| RPIA | Subgroup_I |  |
| IRS4 | Subgroup_I |  |
| B4GALT3 | Subgroup_I |  |
| DNA2L | Subgroup_I |  |
| C17orf74 | Subgroup_I |  |
| MGC11335 | Subgroup_I |  |
| CTSG | Subgroup_I |  |
| GPR113 | Subgroup_I |  |
| FZD7 | Subgroup_I |  |
| MGC2654 | Subgroup_I |  |
| D4ST1 | Subgroup_I |  |
| USP7 | Subgroup_I |  |
| SULT1A3 | Subgroup_I |  |
| FAM79A | Subgroup_I |  |
| CCNI | Subgroup_I |  |
| NBPF1 | Subgroup_I |  |
| IFNAR2 | Subgroup_I |  |
| MAP1LC3C | Subgroup_I |  |
| TMEM86B | Subgroup_I |  |
| RNASE10 | Subgroup_I |  |
| KIAA1838 | Subgroup_I |  |
| TMCC2 | Subgroup_I |  |
| SKIV2L | Subgroup_I |  |
| AGPAT1 | Subgroup_I |  |
| IBRDC3 | Subgroup_I |  |
| FEM1A | Subgroup_I |  |
| TNPO3 | Subgroup_I |  |
| TNS1 | Subgroup_I |  |
| ZNF653 | Subgroup_I |  |
| CLK3 | Subgroup_I |  |
| IRF5 | Subgroup_I |  |
| SOX12 | Subgroup_I |  |
| NIFUN | Subgroup_I |  |
| SUPT5H | Subgroup_I |  |
| FAM100A | Subgroup_I |  |
| PRAME | Subgroup_I |  |
| RNPC1 | Subgroup_I |  |
| TMEM16K | Subgroup_I |  |
| PTPN6 | Subgroup_I |  |
| RNF40 | Subgroup_I |  |
| HK1 | Subgroup_I |  |
| MEP1A | Subgroup_I |  |
| RAVER1 | Subgroup_I |  |
| SKI | Subgroup_I |  |
| CHRNA6 | Subgroup_I |  |
| IHPK3 | Subgroup_I |  |
| BSG | Subgroup_I |  |
| SLC16A5 | Subgroup_I |  |
| SQSTM1 | Subgroup_I |  |
| GAK | Subgroup_I |  |
| CTCF | Subgroup_I |  |
| TBC1D17 | Subgroup_I |  |
| TBC1D22B | Subgroup_I |  |
| SEC22L1 | Subgroup_I |  |
| SRXN1 | Subgroup_I |  |
| MGC42174 | Subgroup_I |  |
| ADD1 | Subgroup_I |  |
| NCOR1 | Subgroup_I |  |
| PA2G4 | Subgroup_I |  |
| FLCN | Subgroup_I |  |
| KPNA6 | Subgroup_I |  |
| POLD4 | Subgroup_I |  |
| ARIH1 | Subgroup_I |  |
| SRC | Subgroup_I | Subgroup_II |
| FIS1 | Subgroup_I |  |
| CCNDBP1 | Subgroup_I |  |
| GATA2 | Subgroup_I |  |
| LOC349136 | Subgroup_I |  |
| FOXJ2 | Subgroup_I |  |
| CDKL1 | Subgroup_I |  |
| ZNF148 | Subgroup_I |  |
| C9orf78 | Subgroup_I |  |
| WBP2 | Subgroup_I |  |
| TRIM58 | Subgroup_I |  |
| C14orf121 | Subgroup_I |  |
| DUSP18 | Subgroup_I |  |
| SLC22A18 | Subgroup_I |  |
| STS-1 | Subgroup_I |  |
| YIPF6 | Subgroup_I |  |
| RSPO2 | Subgroup_I |  |
| MPO | Subgroup_I |  |
| PLCL2 | Subgroup_I |  |
| SLC13A3 | Subgroup_I |  |
| PLAGL2 | Subgroup_I |  |
| STAU1 | Subgroup_I |  |
| IRX1 | Subgroup_I |  |
| EFNA4 | Subgroup_I |  |
| SELENBP1 | Subgroup_I |  |
| HCCA2 | Subgroup_I |  |
| TCP11L2 | Subgroup_I |  |
| FLJ22471 | Subgroup_I |  |
| LECT1 | Subgroup_I |  |
| SERF2 | Subgroup_I |  |
| ROM1 | Subgroup_I |  |
| CD24 | Subgroup_III |  |
| NFAT5 | Subgroup_III | Subgroup_II |
| AP2S1 |  | Subgroup_II |
| FAM50A |  | Subgroup_II |
| AMFR |  | Subgroup_II |
| CCND3 |  | Subgroup_II |
| CEACAM3 |  | Subgroup_II |
| CARD15 |  | Subgroup_II |
| FCER1G |  | Subgroup_II |
| C9orf19 |  | Subgroup_II |
| CHST7 |  | Subgroup_II |
| C3orf40 |  | Subgroup_II |
| C16orf7 |  | Subgroup_II |
| C1orf85 |  | Subgroup_II |
| BRMS1 |  | Subgroup_II |
| DCAKD |  | Subgroup_II |
| BTBD5 |  | Subgroup_II |
| C17orf68 |  | Subgroup_II |
| UBN1 | Subgroup_I |  |
| SPTLC2 | Subgroup_I |  |
| SNAI3 | Subgroup_I |  |
| TOP3A | Subgroup_I |  |
| GAS7 | Subgroup_I |  |
| THRAP5 | Subgroup_I |  |
| SAP130 | Subgroup_I |  |
| SMAP1L | Subgroup_I |  |
| SLC11A1 | Subgroup_I |  |
| VPS37B | Subgroup_I |  |
| UGCGL1 | Subgroup_I |  |
| XPO6 | Subgroup_I |  |
| TRIB1 | Subgroup_I |  |
| ULK1 | Subgroup_I |  |
| SORL1 | Subgroup_I |  |
| SH3GLB1 | Subgroup_I |  |
| RRAGC | Subgroup_I |  |
| SEMA4A | Subgroup_I |  |
| FAM101B | Subgroup_I |  |
| PCNX | Subgroup_I |  |
| AP1G1 | Subgroup_I |  |
| SORT1 | Subgroup_I |  |
| CD177 | Subgroup_I | Subgroup_III |
| LOC401357 | Subgroup_I |  |
| HIP1 | Subgroup_I |  |
| ABR | Subgroup_I |  |
| ADAMTS13 | Subgroup_I | Subgroup_III |
| DNAJB12 | Subgroup_I |  |
| RAB31 | Subgroup_I |  |
| DVL3 | Subgroup_I |  |
| FMNL1 | Subgroup_I |  |
| APOB48R | Subgroup_I |  |
| JMJD3 | Subgroup_I |  |
| FLJ10357 | Subgroup_I |  |
| FLOT1 | Subgroup_I |  |
| IFNAR1 | Subgroup_I |  |
| STAT3 | Subgroup_I |  |
| C1QR1 | Subgroup_I |  |
| MYH9 | Subgroup_I |  |
| KLHL8 | Subgroup_I |  |
| MSL3L1 | Subgroup_I |  |
| PDLIM7 | Subgroup_I |  |
| PGLYRP1 | Subgroup_I |  |
| CEACAM4 | Subgroup_I |  |
| RBAF600 | Subgroup_I |  |
| LOC654052 | Subgroup_I |  |
| EFCBP2 | Subgroup_I |  |
| LYN | Subgroup_I |  |
| RBM23 | Subgroup_I |  |
| C19orf35 | Subgroup_I |  |
| PFKFB4 | Subgroup_I |  |
| SEMA4D | Subgroup_I |  |
| IL8RA | Subgroup_I |  |
| GPR97 | Subgroup_I |  |
| C11orf56 | Subgroup_I |  |
| MYL9 | Subgroup_I |  |
| NPL | Subgroup_I | Subgroup_III |
| DNM2 | Subgroup_I |  |
| PADI4 | Subgroup_I |  |
| MFN2 | Subgroup_I |  |
| KLHDC8B | Subgroup_I |  |
| EXTL3 | Subgroup_I |  |
| HRIHFB2122 | Subgroup_I |  |
| LOC648293 | Subgroup_I |  |
| TALDO1 | Subgroup_I |  |
| LAMP1 | Subgroup_I |  |
| NDEL1 | Subgroup_I |  |
| GMIP | Subgroup_I |  |
| CAP1 | Subgroup_I |  |
| LITAF | Subgroup_I |  |
| MIDN | Subgroup_I |  |
| CA4 | Subgroup_I |  |
| PHF21A | Subgroup_I |  |
| CORO7 | Subgroup_I |  |
| ATP6V1B2 | Subgroup_I |  |
| KIAA0513 | Subgroup_I |  |
| HIST1H4H | Subgroup_I |  |
| NCF1 | Subgroup_I |  |
| ETS2 | Subgroup_I |  |
| OPRL1 | Subgroup_I |  |
| MOSC1 | Subgroup_I |  |
| LILRA3 | Subgroup_I |  |
| FLJ40448 | Subgroup_I |  |
| ATHL1 | Subgroup_I |  |
| MYBPC3 | Subgroup_I |  |
| NRD1 | Subgroup_I |  |
| EDG4 | Subgroup_I |  |
| IGF2R | Subgroup_I |  |
| CBARA1 | Subgroup_I |  |
| GAST | Subgroup_I |  |
| MYST1 | Subgroup_I |  |
| PLAUR | Subgroup_I |  |
| NSFL1C | Subgroup_I |  |
| DCTN1 | Subgroup_I |  |
| TREML2 | Subgroup_I |  |
| DTX2 | Subgroup_I |  |
| OSCAR | Subgroup_I |  |
| OS9 | Subgroup_I |  |
| SFXN5 | Subgroup_I |  |
| ITGB2 | Subgroup_I |  |
| NFE2L1 | Subgroup_I |  |
| SLC6A6 | Subgroup_I |  |
| ICAM3 | Subgroup_I |  |
| MED12 | Subgroup_I |  |
| SSH3 | Subgroup_I |  |
| LOC126208 | Subgroup_I |  |
| HMFN0839 | Subgroup_I |  |
| RBCK1 | Subgroup_I |  |
| DOCK5 | Subgroup_I |  |
| PFKFB3 | Subgroup_I |  |
| PCOLN3 | Subgroup_I |  |
| MAP1LC3B | Subgroup_I |  |
| WAS | Subgroup_I |  |
| C6orf166 | Subgroup_I |  |
| RRBP1 | Subgroup_I |  |
| PGCP | Subgroup_I |  |
| CRISPLD2 | Subgroup_I |  |
| KENAE | Subgroup_I |  |
| KIAA2013 | Subgroup_I |  |
| MSRA | Subgroup_I |  |
| TLE3 | Subgroup_I |  |
| COPB2 | Subgroup_I |  |
| DGCR2 | Subgroup_I |  |
| DOK3 | Subgroup_I |  |
| APS | Subgroup_I |  |
| FLJ14166 | Subgroup_I |  |
| ITGAX | Subgroup_I |  |
| LAPTM5 | Subgroup_I |  |
| MTF1 | Subgroup_I |  |
| PLCG2 | Subgroup_I |  |
| PGM1 | Subgroup_I |  |
| HCLS1 | Subgroup_I |  |
| ATG16L2 | Subgroup_I |  |
| DIAPH1 | Subgroup_I |  |
| ATP6V0D1 | Subgroup_I |  |
| C1RL | Subgroup_I |  |
| ALOX5 | Subgroup_I |  |
| TOLLIP | Subgroup_I |  |
| KIAA1754 | Subgroup_I |  |
| ADAM19 | Subgroup_I |  |
| REPS2 | Subgroup_I | Subgroup_III |
| ADAMTS15 | Subgroup_I | Subgroup_II |
| TIMP2 | Subgroup_I |  |
| MARK2 | Subgroup_I |  |
| LOC284757 | Subgroup_I |  |
| APH1B | Subgroup_I |  |
| PRKCD | Subgroup_I |  |
| RENBP | Subgroup_I |  |
| ADAM8 | Subgroup_I | Subgroup_III |
| CR1 | Subgroup_I |  |
| DHX34 | Subgroup_I |  |
| RFX2 | Subgroup_I |  |
| RIT1 | Subgroup_I |  |
| FLII | Subgroup_I |  |
| NCSTN | Subgroup_I |  |
| C1orf24 | Subgroup_I |  |
| CSNK1D | Subgroup_I |  |
| FLJ11000 | Subgroup_I |  |
| UBTD1 | Subgroup_I |  |
| CMIP | Subgroup_I |  |
| SEPX1 | Subgroup_I |  |
| FLJ25084 | Subgroup_I |  |
| IL8RB | Subgroup_I |  |
| PITPNM2 | Subgroup_I |  |
| PROS1 | Subgroup_I |  |
| MTMR3 | Subgroup_I |  |
| MKL1 | Subgroup_I |  |
| GSN | Subgroup_I |  |
| TIAM2 | Subgroup_I |  |
| TMEM185A | Subgroup_I |  |
| LSP1 | Subgroup_I |  |
| LCN2 | Subgroup_I |  |
| TST | Subgroup_I |  |
| MBD6 | Subgroup_I |  |
| PGD | Subgroup_I |  |
| XKR8 | Subgroup_I |  |
| CEBPB | Subgroup_I |  |
| CAMK1D | Subgroup_I |  |
| ANPEP | Subgroup_I |  |
| PACSIN2 | Subgroup_I |  |
| LIN7A | Subgroup_I |  |
| KIAA0274 | Subgroup_I |  |
| EIF2C4 | Subgroup_I |  |
| FLJ20186 | Subgroup_I |  |
| MTX1 | Subgroup_I |  |
| ATP6V0A1 | Subgroup_I |  |
| MMP9 | Subgroup_I |  |
| C20orf149 | Subgroup_I |  |
| ALPK1 | Subgroup_I |  |
| MYO9B | Subgroup_I |  |
| FPR1 | Subgroup_I |  |
| DBN1 | Subgroup_I |  |
| IL6R | Subgroup_I |  |
| ARID3A | Subgroup_I |  |
| ST3GAL4 | Subgroup_I |  |
| DNAJC5 | Subgroup_I |  |
| GRB2 | Subgroup_I |  |
| CNN2 | Subgroup_I |  |
| TLR8 | Subgroup_I | Subgroup_II |
| RARA | Subgroup_I |  |
| CTNNA1 | Subgroup_I |  |
| ACSS2 | Subgroup_I |  |
| LOC201175 | Subgroup_I |  |
| NLRP1 | Subgroup_I | Subgroup_III |
| BRD3 | Subgroup_I |  |
| EIF4G3 | Subgroup_I |  |
| FKBP9L | Subgroup_I |  |
| NCOA6 | Subgroup_I |  |
| IQSEC1 | Subgroup_I |  |
| MYD88 | Subgroup_I |  |
| THOC5 | Subgroup_I |  |
| LEPROT | Subgroup_I |  |
| RAB7 | Subgroup_I |  |
| PTP4A3 | Subgroup_I |  |
| GABARAPL1 | Subgroup_I |  |
| KIAA1324 | Subgroup_I |  |
| HK3 | Subgroup_I |  |
| LILRA5 | Subgroup_I |  |
| LOC653723 | Subgroup_I |  |
| PIK3CD | Subgroup_I |  |
| RALB | Subgroup_I |  |
| IL33 | Subgroup_I | Subgroup_II |
| LRRC25 | Subgroup_I |  |
| DYSF | Subgroup_I |  |
| DENND1C | Subgroup_I |  |
| PPP3R1 | Subgroup_I |  |
| SEMA4B | Subgroup_I |  |
| TAPBP | Subgroup_I |  |
| TOM1 | Subgroup_I |  |
| VCP | Subgroup_I |  |
| CLTCL1 | Subgroup_I |  |
| DDAH2 | Subgroup_I |  |
| MAP1LC3A | Subgroup_I |  |
| EIF2C1 | Subgroup_I |  |
| P2RX1 | Subgroup_I |  |
| MYO7B | Subgroup_I |  |
| PSTPIP1 | Subgroup_I |  |
| GBA | Subgroup_I |  |
| SFRS2IP | Subgroup_I |  |
| RPS6KA1 | Subgroup_I |  |
| SOLH | Subgroup_I |  |
| MAPK1 | Subgroup_I |  |
| EPHB1 | Subgroup_I |  |
| NDST1 | Subgroup_I |  |
| NALP12 | Subgroup_I |  |
| FAH | Subgroup_I |  |
| ORF1-FL49 | Subgroup_I |  |
| DBNL | Subgroup_I |  |
| RNF148 | Subgroup_I |  |
| FLOT2 | Subgroup_I |  |
| VPS37C | Subgroup_I |  |
| ANXA11 | Subgroup_I |  |
| SLC15A3 | Subgroup_I |  |
| EGFL5 | Subgroup_I |  |
| C20orf43 | Subgroup_I |  |
| PDK3 | Subgroup_I |  |
| KIAA0404 | Subgroup_I |  |
| OXSR1 | Subgroup_I |  |
| LOC653098 | Subgroup_I |  |
| C3 | Subgroup_I | Subgroup_II |
| ZYX | Subgroup_I |  |
| LOC400499 | Subgroup_I |  |
| CAMP | Subgroup_I |  |
| F5 | Subgroup_I |  |
| FUT7 | Subgroup_I |  |
| KLRG2 | Subgroup_I |  |
| SRPK1 | Subgroup_I |  |
| GSK3B | Subgroup_I |  |
| PHC2 | Subgroup_I |  |
| LIMK2 | Subgroup_I |  |
| NRGN | Subgroup_I |  |
| SSH2 | Subgroup_I |  |
| ENTPD1 | Subgroup_I |  |
| DENND3 | Subgroup_I |  |
| C19orf7 | Subgroup_I |  |
| DAPK2 | Subgroup_I |  |
| ANKRD13 | Subgroup_I |  |
| FGR | Subgroup_I |  |
| TLR4 | Subgroup_I | Subgroup_III |
| RAB11FIP1 | Subgroup_I |  |
| MGRN1 | Subgroup_I |  |
| DNAJB6 | Subgroup_I |  |
| LRG1 | Subgroup_I |  |
| C22orf5 | Subgroup_I |  |
| MYO1F | Subgroup_I |  |
| MRVI1 | Subgroup_I |  |
| NEXN | Subgroup_I |  |
| TRPM6 | Subgroup_I |  |
| TP53I11 | Subgroup_I |  |
| RAB43 | Subgroup_I |  |
| RENT1 | Subgroup_I |  |
| DAB2 | Subgroup_I |  |
| ZNF438 | Subgroup_I |  |
| B3GNTL1 | Subgroup_I |  |
| PLOD1 | Subgroup_I |  |
| HIST1H2BD | Subgroup_I |  |
| IL1RN | Subgroup_I |  |
| N4BP1 | Subgroup_I |  |
| LMNB1 | Subgroup_I |  |
| MAP3K11 | Subgroup_I |  |
| SAMD4B | Subgroup_I |  |
| KATNB1 | Subgroup_I |  |
| ITGB5 | Subgroup_I |  |
| FLJ31413 | Subgroup_I |  |
| OSTF1 | Subgroup_I |  |
| NOD9 | Subgroup_I |  |
| MVP | Subgroup_I |  |
| ANTXR2 | Subgroup_I |  |
| PIAS1 | Subgroup_I |  |
| SELL | Subgroup_I |  |
| Rgr | Subgroup_I |  |
| TBL1X | Subgroup_I |  |
| VCL | Subgroup_I |  |
| SLC35A2 | Subgroup_I |  |
| USP32 | Subgroup_I |  |
| SLC2A3 | Subgroup_I |  |
| IHPK1 | Subgroup_I |  |
| ABCA7 | Subgroup_I |  |
| RYBP | Subgroup_I |  |
| SNTB2 | Subgroup_I |  |
| SSH1 | Subgroup_I |  |
| TIMP1 | Subgroup_I |  |
| H1F0 | Subgroup_I |  |
| ZNF213 | Subgroup_I |  |
| NPEPL1 | Subgroup_I |  |
| IL17R | Subgroup_I |  |
| VPS24 | Subgroup_I |  |
| ZFP106 | Subgroup_I |  |
| DISC1 | Subgroup_I |  |
| IMPDH1 | Subgroup_I |  |
| ATP9A | Subgroup_I |  |
| HNRPUL2 | Subgroup_I |  |
| CENTD3 | Subgroup_I |  |
| ITGA2B | Subgroup_I |  |
| PSORS1C1 | Subgroup_I |  |
| HIPK2 | Subgroup_I |  |
| TNFRSF10C | Subgroup_I |  |
| MLL4 | Subgroup_I |  |
| VPS18 | Subgroup_I |  |
| NUAK2 | Subgroup_I |  |
| SLC26A8 | Subgroup_I |  |
| PAK1 | Subgroup_I |  |
| CHD4 | Subgroup_I |  |
| RASSF2 | Subgroup_I |  |
| DPEP2 | Subgroup_I |  |
| LOC349236 | Subgroup_I |  |
| GPR141 | Subgroup_I |  |
| TGM3 | Subgroup_I |  |
| GRN | Subgroup_I |  |
| GAA | Subgroup_I |  |
| PRKCZ | Subgroup_I |  |
| APBB1IP | Subgroup_I |  |
| DOCK2 | Subgroup_I |  |
| SIRPB1 | Subgroup_I |  |
| CASZ1 | Subgroup_I |  |
| SLCO3A1 | Subgroup_I |  |
| CRKRS | Subgroup_I |  |
| OSBPL1A | Subgroup_I |  |
| ZYG11B | Subgroup_I |  |
| MNT | Subgroup_I |  |
| IL18RAP | Subgroup_I |  |
| CETP | Subgroup_I |  |
| HIST2H2AC | Subgroup_I |  |
| OPLAH | Subgroup_I |  |
| ALPL | Subgroup_I |  |
| RAB37 | Subgroup_I |  |
| BTBD14A | Subgroup_I |  |
| AQP9 | Subgroup_I |  |
| VPS8 | Subgroup_I |  |
| TMEM45B | Subgroup_I |  |
| ZNFX1 | Subgroup_I |  |
| BRSK1 | Subgroup_I | Subgroup_II |
| IL1R2 | Subgroup_I | Subgroup_III |
| FCGRT | Subgroup_I |  |
| GNS | Subgroup_I |  |
| RGS14 | Subgroup_I |  |
| LTB4R | Subgroup_I |  |
| C10orf54 | Subgroup_I |  |
| HIST1H2BK | Subgroup_I |  |
| TREML1 | Subgroup_I |  |
| SH3BP5L | Subgroup_I |  |
| IL4R | Subgroup_I |  |
| CHMP2A | Subgroup_I |  |
| COPG | Subgroup_I |  |
| KIAA0690 | Subgroup_I |  |
| NUDT16 | Subgroup_I |  |
| SOD2 | Subgroup_I |  |
| GATAD2B | Subgroup_I |  |
| ZMYND15 | Subgroup_I |  |
| PRMT5 | Subgroup_I |  |
| PTGS1 | Subgroup_I |  |
| LCP1 | Subgroup_I |  |
| UBR2 | Subgroup_I |  |
| ECHDC3 | Subgroup_I |  |
| AXUD1 | Subgroup_I | Subgroup_II |
| TMEM91 | Subgroup_I |  |
| ELMO1 | Subgroup_I |  |
| RXRA | Subgroup_I |  |
| FKBP5 | Subgroup_I |  |
| MX2 | Subgroup_I |  |
| TTLL4 | Subgroup_I |  |
| CXCL16 | Subgroup_I |  |
| C16orf28 | Subgroup_I |  |
| NARF | Subgroup_I |  |
| LKAP | Subgroup_I |  |
| JARID2 | Subgroup_I |  |
| PHF20 | Subgroup_I |  |
| LBR | Subgroup_I |  |
| HIST2H2AA3 | Subgroup_I |  |
| SIGLEC5 | Subgroup_I |  |
| SMARCD2 | Subgroup_I |  |
| NQO2 | Subgroup_I |  |
| FLJ22662 | Subgroup_I |  |
| TRIM21 | Subgroup_I |  |
| CARD12 | Subgroup_I |  |
| TBC1D14 | Subgroup_I |  |
| IRAK3 | Subgroup_I |  |
| RABGAP1 | Subgroup_I |  |
| KIAA1706 | Subgroup_I |  |
| STK4 | Subgroup_I |  |
| STK40 | Subgroup_I |  |
| TBXAS1 | Subgroup_I |  |
| TCIRG1 | Subgroup_I |  |
| RRAGD | Subgroup_I |  |
| PRIC285 | Subgroup_I |  |
| SLC9A1 | Subgroup_I |  |
| ZFAND3 | Subgroup_I |  |
| C16orf57 | Subgroup_I |  |
| ATG7 | Subgroup_I |  |
| RPRC1 | Subgroup_I |  |
| ROPN1L | Subgroup_I |  |
| NR1H2 | Subgroup_I |  |
| RIN3 | Subgroup_I |  |
| HS1BP3 | Subgroup_I |  |
| RNF31 | Subgroup_I |  |
| IGF1R | Subgroup_I |  |
| CALCOCO2 | Subgroup_I |  |
| BCKDK | Subgroup_I |  |
| TMEM11 | Subgroup_I |  |
| AKAP8L | Subgroup_I |  |
| TLR6 | Subgroup_I | Subgroup_II |
| LRP3 | Subgroup_I |  |
| THBS1 | Subgroup_I |  |
| STARD10 | Subgroup_I |  |
| CLDN9 | Subgroup_I |  |
| TNFRSF9 | Subgroup_I | Subgroup_II |
| RNF24 | Subgroup_I |  |
| TSC22D3 | Subgroup_II |  |
| FLJ10379 | Subgroup_II | Subgroup_III |
| CCPG1 | Subgroup_II | Subgroup_III |
| IL1F7 | Subgroup_II | Subgroup_III |
| PDPK1 | Subgroup_II | Subgroup_I |
| MMP25 | Subgroup_II | Subgroup_III |
| TLR2 | Subgroup_III | Subgroup_II |
| CXCL2 | Subgroup_III | Subgroup_II |
| BAZ1A | Subgroup_III |  |
| IL13RA1 | Subgroup_III | Subgroup_II |
| IL10RB | Subgroup_III | Subgroup_II |
| TREM1 | Subgroup_III | Subgroup_II |
| IL2 | Subgroup_III | Subgroup_II |
| SULT1A2 |  | Subgroup_III |
| PFC |  | Subgroup_III |
| WDR52 |  | Subgroup_III |
| DIP13B |  | Subgroup_II |
| PSENEN |  | Subgroup_III |
| ADM |  | Subgroup_II |
| PADI2 |  | Subgroup_III |
| PLAC2 |  | Subgroup_III |
| BPY2IP1 |  | Subgroup_III |
| KIDINS220 |  | Subgroup_III |
| BCL6 |  | Subgroup_II |
| KRT23 |  | Subgroup_III |
| SQRDL |  | Subgroup_III |
| NPEPPS |  | Subgroup_III |
| ZNF93 |  | Subgroup_III |
| HOXC11 |  | Subgroup_III |
| EGLN1 |  | Subgroup_III |
| ZNF537 |  | Subgroup_III |
| HECW2 |  | Subgroup_III |
| ACOX1 |  | Subgroup_III |
| MME |  | Subgroup_III |
| STXBP5 |  | Subgroup_III |
| KLHL18 |  | Subgroup_III |
| VNN3 |  | Subgroup_III |
| RBP7 |  | Subgroup_III |
| LAT2 |  | Subgroup_III |
| SCG3 |  | Subgroup_III |
| MGAM |  | Subgroup_III |
| LOC349114 |  | Subgroup_III |
| C3orf17 |  | Subgroup_III |
| MLF2 |  | Subgroup_III |
| PDZD8 |  | Subgroup_III |
| KIF1B |  | Subgroup_III |
| TMPIT |  | Subgroup_III |
| LOC649377 |  | Subgroup_III |
| NCOA1 |  | Subgroup_III |
| DAAM2 |  | Subgroup_III |
| CD300LB |  | Subgroup_II |
| CREBBP |  | Subgroup_III |
| SVIL |  | Subgroup_III |
| LENG4 |  | Subgroup_III |
| CDS2 |  | Subgroup_II |
| PBEF1 |  | Subgroup_III |
| NDRG1 |  | Subgroup_III |
| RNF122 |  | Subgroup_III |
| ANKRD22 |  | Subgroup_II |
| HLA-E |  | Subgroup_III |
| GAB2 |  | Subgroup_III |
| HCK |  | Subgroup_III |
| SDCBP |  | Subgroup_III |
| CXorf38 |  | Subgroup_II |
| KRAS |  | Subgroup_III |
| PLXDC2 |  | Subgroup_III |
| PLEKHQ1 |  | Subgroup_III |
| GLT1D1 |  | Subgroup_III |
| DDEF1 |  | Subgroup_III |
| MLSTD1 |  | Subgroup_III |
| PLP2 |  | Subgroup_III |
| ARG1 |  | Subgroup_III |
| C1orf38 |  | Subgroup_III |
| TNFRSF4 | Subgroup_I | Subgroup_II |
| LTBP4 | Subgroup_I | Subgroup_II |
| TRAP1 | Subgroup_I | Subgroup_II |
| KIAA0495 | Subgroup_I | Subgroup_III |
| IL18BP | Subgroup_I | Subgroup_II |
| ELAC2 | Subgroup_I | Subgroup_II |
| TNFRSF13B | Subgroup_I | Subgroup_II |
| TNFRSF7 | Subgroup_II | Subgroup_I |
| TNFRSF13C | Subgroup_II | Subgroup_I |
| SHFM1 | Subgroup_III |  |
| TMEM50B | Subgroup_III |  |
| RPL8 | Subgroup_III |  |
| ZFP90 | Subgroup_III |  |
| WRNIP1 | Subgroup_III |  |
| MYH3 | Subgroup_III |  |
| ZNF135 | Subgroup_III |  |
| SPCS1 | Subgroup_III |  |
| TAP1 | Subgroup_III |  |
| RPS3 | Subgroup_III |  |
| TSPAN31 | Subgroup_III |  |
| RPL38 | Subgroup_III |  |
| UQCRFS1 | Subgroup_III |  |
| RPP21 | Subgroup_III |  |
| TMEM101 | Subgroup_III | Subgroup_II |
| EVL | Subgroup_III |  |
| ZBTB25 | Subgroup_III |  |
| SMARCAD1 | Subgroup_III |  |
| TMEM32 | Subgroup_III |  |
| MGC3207 | Subgroup_III |  |
| VAMP5 | Subgroup_III |  |
| RBM5 | Subgroup_III |  |
| SFRS5 | Subgroup_III |  |
| WBSCR18 | Subgroup_III |  |
| GPR89A | Subgroup_III |  |
| TIA1 | Subgroup_III |  |
| SFRS14 | Subgroup_III |  |
| TNFRSF10A | Subgroup_III |  |
| MXD4 | Subgroup_III |  |
| RWDD1 | Subgroup_III |  |
| TF | Subgroup_III | Subgroup_II |
| GIMAP5 | Subgroup_III |  |
| THNSL1 | Subgroup_III |  |
| TMEM59 | Subgroup_III |  |
| IL11RA | Subgroup_III | Subgroup_II |
| DAP3 | Subgroup_III |  |
| RPS9 | Subgroup_III |  |
| PPP3CB | Subgroup_III |  |
| SNRPA1 | Subgroup_III |  |
| DNAJC8 | Subgroup_III |  |
| UFC1 | Subgroup_III | Subgroup_II |
| LCMT2 | Subgroup_III |  |
| ICOS | Subgroup_III |  |
| SDF2L1 | Subgroup_III |  |
| C10orf35 | Subgroup_III |  |
| TBCA | Subgroup_III |  |
| YWHAQ | Subgroup_III | Subgroup_I |
| LOC400464 | Subgroup_III |  |
| C9orf123 | Subgroup_III |  |
| GPR18 | Subgroup_III |  |
| DENND2D | Subgroup_III |  |
| MTX3 | Subgroup_III |  |
| AXIN2 | Subgroup_III |  |
| RBM17 | Subgroup_III |  |
| SKP2 | Subgroup_III |  |
| TMEM123 | Subgroup_III | Subgroup_I |
| MRPL46 | Subgroup_III |  |
| ATP6V0E2L | Subgroup_III |  |
| OR2A9P | Subgroup_III |  |
| NELL2 | Subgroup_III |  |
| FLJ10178 | Subgroup_III |  |
| KIAA2010 | Subgroup_III |  |
| AGMAT | Subgroup_III |  |
| MGC17330 | Subgroup_III |  |
| C1orf109 | Subgroup_III |  |
| CCR7 | Subgroup_III |  |
| PASK | Subgroup_III |  |
| MGC19764 | Subgroup_III |  |
| ZNF700 | Subgroup_III |  |
| EMG1 | Subgroup_III |  |
| DPH5 | Subgroup_III |  |
| APEX1 | Subgroup_III |  |
| BEX2 | Subgroup_III |  |
| C6orf130 | Subgroup_III |  |
| MGC15763 | Subgroup_III |  |
| C11orf1 | Subgroup_III |  |
| BNIP3 | Subgroup_III |  |
| C6orf48 | Subgroup_III |  |
| IL23A | Subgroup_III |  |
| ZNF277 | Subgroup_III |  |
| C12orf57 | Subgroup_III |  |
| TMED5 | Subgroup_III | Subgroup_I |
| LOC388524 | Subgroup_III |  |
| TIGD7 | Subgroup_III |  |
| SLC11A2 | Subgroup_III |  |
| FLJ11171 | Subgroup_III | Subgroup_I |
| PSMA5 | Subgroup_III |  |
| IL2RB | Subgroup_III | Subgroup_I |
| ZDHHC8 | Subgroup_III |  |
| KLF9 | Subgroup_III |  |
| MRPL22 | Subgroup_III |  |
| POLE4 | Subgroup_III |  |
| GALNT11 | Subgroup_III | Subgroup_I |
| FLJ20160 | Subgroup_III |  |
| TRIM52 | Subgroup_III |  |
| SNX2 | Subgroup_III |  |
| FASLG | Subgroup_III |  |
| C14orf135 | Subgroup_III |  |
| SLC35B3 | Subgroup_III |  |
| PSMA6 | Subgroup_III | Subgroup_I |
| STX16 | Subgroup_III |  |
| TIMM22 | Subgroup_III |  |
| GZMM | Subgroup_III |  |
| ELA1 | Subgroup_III |  |
| ATP5H | Subgroup_III |  |
| MGC4562 | Subgroup_III |  |
| TMEM85 | Subgroup_III |  |
| HPRT1 | Subgroup_III | Subgroup_I |
| CD69 | Subgroup_III | Subgroup_I |
| NDUFS4 | Subgroup_III |  |
| DDX39 | Subgroup_III |  |
| ARMET | Subgroup_III |  |
| HCST | Subgroup_III |  |
| FUT4 | Subgroup_III |  |
| RPL35A | Subgroup_III |  |
| QIL1 | Subgroup_III |  |
| TNFRSF6B | Subgroup_III | Subgroup_I |
| NIFIE14 | Subgroup_III |  |
| ACAT1 | Subgroup_III |  |
| SESN1 | Subgroup_III |  |
| MRPL33 | Subgroup_III |  |
| RPL32 | Subgroup_III |  |
| FAM96B | Subgroup_III |  |
| IL32 | Subgroup_III | Subgroup_I |
| LAX1 | Subgroup_III |  |
| CNIH | Subgroup_III | Subgroup_I |
| NCK1 | Subgroup_III |  |
| RNF125 | Subgroup_III |  |
| C2orf28 | Subgroup_III |  |
| RYK | Subgroup_III | Subgroup_I |
| PSMB7 | Subgroup_III |  |
| STX2 | Subgroup_III |  |
| SAMD3 | Subgroup_III |  |
| LRP5L | Subgroup_III |  |
| C11orf10 | Subgroup_III |  |
| SORBS3 | Subgroup_III |  |
| C14orf147 | Subgroup_III |  |
| QP-C | Subgroup_III |  |
| C1orf19 | Subgroup_III |  |
| HSPC176 | Subgroup_III |  |
| ZNF514 | Subgroup_III |  |
| PGBD2 | Subgroup_III |  |
| MAPK6 | Subgroup_III |  |
| C8orf35 | Subgroup_III |  |
| S100A10 | Subgroup_III |  |
| NDFIP1 | Subgroup_III |  |
| RPS17 | Subgroup_III | Subgroup_I |
| NCALD | Subgroup_III |  |
| CDC26 | Subgroup_III |  |
| ARPC5L | Subgroup_III |  |
| ZNF529 | Subgroup_III |  |
| IL28RA | Subgroup_III | Subgroup_I |
| CCDC56 | Subgroup_III |  |
| ARHGEF3 | Subgroup_III | Subgroup_I |
| EIF4A1 | Subgroup_III |  |
| RPS14 | Subgroup_III |  |
| SCMH1 | Subgroup_III |  |
| IL10RA | Subgroup_III | Subgroup_II |
| BTBD6 | Subgroup_III |  |
| LOC205251 | Subgroup_III |  |
| MRPL41 | Subgroup_III |  |
| RASSF1 | Subgroup_III |  |
| FLJ20512 | Subgroup_III |  |
| ZFP161 | Subgroup_III |  |
| MRPS21 | Subgroup_III |  |
| AUTS2 | Subgroup_III |  |
| CYC1 | Subgroup_III |  |
| RPL22 | Subgroup_III |  |
| DNAJB11 | Subgroup_III |  |
| C19orf10 | Subgroup_III |  |
| COX17 | Subgroup_III |  |
| SELS | Subgroup_III |  |
| RPL26L1 | Subgroup_III |  |
| MRPL54 | Subgroup_III |  |
| MTMR9 | Subgroup_III |  |
| C20orf52 | Subgroup_III |  |
| MCM3APAS | Subgroup_III |  |
| TMEM173 | Subgroup_III |  |
| TFAM | Subgroup_III |  |
| TNF | Subgroup_III | Subgroup_II |
| TARBP1 | Subgroup_III |  |
| MATK | Subgroup_III |  |
| RPS10 | Subgroup_III |  |
| RPL27 | Subgroup_III |  |
| MRPL9 | Subgroup_III |  |
| NDUFS5 | Subgroup_III |  |
| COX7A2 | Subgroup_III |  |
| PGBD4 | Subgroup_III |  |
| NOL7 | Subgroup_III |  |
| TP53RK | Subgroup_III |  |
| UBE2N | Subgroup_III | Subgroup_I |
| SRPRB | Subgroup_III |  |
| DERL1 | Subgroup_III | Subgroup_I |
| PLEKHF1 | Subgroup_III |  |
| LEMD2 | Subgroup_III |  |
| SLC20A1 | Subgroup_III |  |
| RUTBC1 | Subgroup_III |  |
| DPM3 | Subgroup_III | Subgroup_II |
| C10orf33 | Subgroup_III |  |
| ITR | Subgroup_III | Subgroup_I |
| RBM12B | Subgroup_III |  |
| BCL7C | Subgroup_III |  |
| HLA-DMB | Subgroup_III |  |
| LSM1 | Subgroup_III |  |
| PSARL | Subgroup_III |  |
| CXXC5 | Subgroup_III |  |
| HLA-DPB1 | Subgroup_III |  |
| MRPS17 | Subgroup_III |  |
| PTGDR | Subgroup_III |  |
| COX5A | Subgroup_III |  |
| TGDS | Subgroup_III |  |
| PLAC8 | Subgroup_III |  |
| RPL12 | Subgroup_III |  |
| RPS5 | Subgroup_III |  |
| GTPBP4 | Subgroup_III |  |
| NENF | Subgroup_III |  |
| LOC119710 | Subgroup_III |  |
| MTFR1 | Subgroup_III | Subgroup_I |
| SLC35B2 | Subgroup_III |  |
| RPA3 | Subgroup_III |  |
| HLA-DRA | Subgroup_III |  |
| RPL13A | Subgroup_III |  |
| ELAC1 | Subgroup_III | Subgroup_I |
| GPR114 | Subgroup_III |  |
| CA5B | Subgroup_III |  |
| MRPL40 | Subgroup_III |  |
| SSR1 | Subgroup_III |  |
| PYHIN1 | Subgroup_III |  |
| NDUFA1 | Subgroup_III |  |
| RPS4X | Subgroup_III |  |
| ANXA1 | Subgroup_III |  |
| ZBTB40 | Subgroup_III |  |
| NDUFB7 | Subgroup_III |  |
| CDC40 | Subgroup_III |  |
| FCRL6 | Subgroup_III |  |
| HLA-DMA | Subgroup_III |  |
| GZMK | Subgroup_III |  |
| C12orf32 | Subgroup_III |  |
| MED4 | Subgroup_III |  |
| RPAIN | Subgroup_III |  |
| STK39 | Subgroup_III |  |
| SELM | Subgroup_III |  |
| LOC642090 | Subgroup_III | Subgroup_I |
| HOP | Subgroup_III | Subgroup_I |
| SS18L2 | Subgroup_III |  |
| ACTL6A | Subgroup_III |  |
| DERA | Subgroup_III |  |
| GIMAP6 | Subgroup_III |  |
| GPR56 | Subgroup_III |  |
| RDH14 | Subgroup_III | Subgroup_I |
| NAT5 | Subgroup_III |  |
| TMEM62 | Subgroup_III |  |
| PCMT1 | Subgroup_III |  |
| SEC22C | Subgroup_III |  |
| NPC1 | Subgroup_III |  |
| NDUFB2 | Subgroup_III |  |
| DBI | Subgroup_III | Subgroup_I |
| CXCR3 | Subgroup_III | Subgroup_I |
| RGPD1 | Subgroup_III |  |
| AHCY | Subgroup_III |  |
| ZNFN1A5 | Subgroup_III |  |
| D4S234E | Subgroup_III |  |
| PCGF4 | Subgroup_III | Subgroup_I |
| MDH1 | Subgroup_III |  |
| TNFRSF14 | Subgroup_III |  |
| MRPL51 | Subgroup_III |  |
| RPS26 | Subgroup_III |  |
| CXCR6 | Subgroup_III | Subgroup_II |
| STAMBPL1 | Subgroup_III |  |
| PTGER2 | Subgroup_III |  |
| PDHB | Subgroup_III |  |
| BTG1 | Subgroup_III |  |
| ZNHIT3 | Subgroup_III | Subgroup_I |
| CLDND1 | Subgroup_III | Subgroup_I |
| CIP29 | Subgroup_III |  |
| MDP-1 | Subgroup_III |  |
| SLC35A3 | Subgroup_III | Subgroup_I |
| MRPS22 | Subgroup_III |  |
| NT5C3L | Subgroup_III |  |
| PXMP3 | Subgroup_III |  |
| FLJ20054 | Subgroup_III |  |
| UQCRH | Subgroup_III | Subgroup_I |
| BTLA | Subgroup_III |  |
| COMMD3 | Subgroup_III |  |
| WDR54 | Subgroup_III |  |
| CR2 | Subgroup_III | Subgroup_I |
| TOMM7 | Subgroup_III |  |
| LRP8 | Subgroup_III | Subgroup_I |
| MTP18 | Subgroup_III |  |
| MGC40579 | Subgroup_III |  |
| STAT4 | Subgroup_III |  |
| RORA | Subgroup_III |  |
| CD40 | Subgroup_III | Subgroup_II |
| MCEE | Subgroup_III |  |
| MMP21 | Subgroup_III | Subgroup_II |
| TMEM5 | Subgroup_III |  |
| CDK4 | Subgroup_III |  |
| BTAF1 | Subgroup_III | Subgroup_I |
| RPL4 | Subgroup_III |  |
| C17orf61 | Subgroup_III |  |
| TOR3A | Subgroup_III |  |
| TMEM87A | Subgroup_III |  |
| HSPC023 | Subgroup_III |  |
| C6orf125 | Subgroup_III |  |
| DUSP12 | Subgroup_III | Subgroup_I |
| PTPRM | Subgroup_III |  |
| DCUN1D4 | Subgroup_III |  |
| NUPL2 | Subgroup_III |  |
| TMED4 | Subgroup_III |  |
| LIME1 | Subgroup_III |  |
| KIAA0143 | Subgroup_III |  |
| TLR3 | Subgroup_III | Subgroup_II |
| C21orf66 | Subgroup_III |  |
| IL17RB | Subgroup_III | Subgroup_I |
| KCNK12 | Subgroup_III |  |
| ZCD1 | Subgroup_III |  |
| TNFAIP8 | Subgroup_III |  |
| DNAJB9 | Subgroup_III |  |
| POLR3GL | Subgroup_III |  |
| KSP37 | Subgroup_III |  |
| MGC52110 | Subgroup_III | Subgroup_I |
| LSM2 | Subgroup_III |  |
| UNC50 | Subgroup_III |  |
| LOC168850 | Subgroup_III |  |
| RPA2 | Subgroup_III |  |
| DDX5 | Subgroup_III |  |
| RUNX3 | Subgroup_III |  |
| C8orf40 | Subgroup_III |  |
| RSL1D1 | Subgroup_III |  |
| MRPL43 | Subgroup_III |  |
| LRAP | Subgroup_III |  |
| OGT | Subgroup_III |  |
| EXOSC1 | Subgroup_III |  |
| C10orf32 | Subgroup_III | Subgroup_I |
| GEMIN6 | Subgroup_III |  |
| RPL27A | Subgroup_III |  |
| RARRES3 | Subgroup_III |  |
| MRPS26 | Subgroup_III |  |
| RSBN1 | Subgroup_III |  |
| KLRB1 | Subgroup_III | Subgroup_I |
| HMGN1 | Subgroup_III | Subgroup_I |
| EBNA1BP2 | Subgroup_III |  |
| CRIP1 | Subgroup_III |  |
| NDUFA8 | Subgroup_III |  |
| C16orf61 | Subgroup_III |  |
| EDG8 | Subgroup_III |  |
| FLJ20647 | Subgroup_III | Subgroup_I |
| OXCT1 | Subgroup_III |  |
| THRAP6 | Subgroup_III |  |
| FKBP11 | Subgroup_III |  |
| MRPS6 | Subgroup_III |  |
| SEC22L2 | Subgroup_III |  |
| MTERFD1 | Subgroup_III |  |
| ZNF22 | Subgroup_III | Subgroup_I |
| HSPA14 | Subgroup_III |  |
| HADHSC | Subgroup_III |  |
| HAVCR2 | Subgroup_III |  |
| INSIG2 | Subgroup_III |  |
| ANKRD36 | Subgroup_III |  |
| PARK7 | Subgroup_III |  |
| TCF12 | Subgroup_III |  |
| RPL10A | Subgroup_III |  |
| HEL308 | Subgroup_III |  |
| KIAA1826 | Subgroup_III |  |
| LSM7 | Subgroup_III |  |
| PAM | Subgroup_III |  |
| C1orf41 | Subgroup_III |  |
| COMMD7 | Subgroup_III |  |
| MRPL36 | Subgroup_III |  |
| C11orf46 | Subgroup_III | Subgroup_I |
| SFRS2 | Subgroup_III | Subgroup_I |
| C2orf30 | Subgroup_III | Subgroup_I |
| TLR7 | Subgroup_III |  |
| C5orf15 | Subgroup_III |  |
| C8orf76 | Subgroup_III |  |
| SNRPC | Subgroup_III |  |
| C6orf129 | Subgroup_III |  |
| PHF5A | Subgroup_III |  |
| EPSTI1 | Subgroup_III |  |
| TMEM116 | Subgroup_III | Subgroup_II |
| IKZF3 | Subgroup_III |  |
| RPS2 | Subgroup_III |  |
| SNRPG | Subgroup_III | Subgroup_I |
| ZNF337 | Subgroup_III |  |
| LOC84661 | Subgroup_III |  |
| NDUFB5 | Subgroup_III |  |
| RIP | Subgroup_III |  |
| UXT | Subgroup_III |  |
| GALNT12 | Subgroup_III | Subgroup_I |
| NKG7 | Subgroup_III |  |
| TNFSF12 | Subgroup_III | Subgroup_II |
| TRAM1 | Subgroup_III | Subgroup_I |
| CDC16 | Subgroup_III |  |
| PRKRIR | Subgroup_III | Subgroup_I |
| NFKBIZ | Subgroup_III |  |
| MMP11 | Subgroup_III | Subgroup_II |
| QARS | Subgroup_III |  |
| ZNF17 | Subgroup_III |  |
| PTRH2 | Subgroup_III |  |
| PTPN4 | Subgroup_III |  |
| C18orf55 | Subgroup_III |  |
| PARP15 | Subgroup_III |  |
| C12orf41 | Subgroup_III |  |
| BXDC1 | Subgroup_III | Subgroup_I |
| ATP5I | Subgroup_III |  |
| NKIRAS1 | Subgroup_III |  |
| LOC219854 | Subgroup_III |  |
| EXOSC6 | Subgroup_III |  |
| MRPL11 | Subgroup_III |  |
| SSR2 | Subgroup_III |  |
| CLNS1A | Subgroup_III | Subgroup_I |
| CMAH | Subgroup_III |  |
| IRF8 | Subgroup_III |  |
| SFRS6 | Subgroup_III |  |
| SCAMP1 | Subgroup_III |  |
| GFI1 | Subgroup_III |  |
| ZNF443 | Subgroup_III |  |
| EIF3S5 | Subgroup_III |  |
| MMAB | Subgroup_III |  |
| CYP4V2 | Subgroup_III |  |
| METAP1 | Subgroup_III |  |
| WDR36 | Subgroup_III |  |
| WDR19 | Subgroup_III |  |
| CD81 | Subgroup_III |  |
| OXR1 | Subgroup_III | Subgroup_I |
| DOCK10 | Subgroup_III |  |
| PEX1 | Subgroup_III |  |
| SFRS9 | Subgroup_III |  |
| TNFRSF25 | Subgroup_III |  |
| HSPA8 | Subgroup_III | Subgroup_I |
| MED28 | Subgroup_III |  |
| MTO1 | Subgroup_III | Subgroup_I |
| KCNA3 | Subgroup_III |  |
| LOC646200 | Subgroup_III |  |
| SYTL2 | Subgroup_III |  |
| NDUFA12 | Subgroup_III |  |
| FLJ22531 | Subgroup_III |  |
| FAM24B | Subgroup_III |  |
| FAM44B | Subgroup_III |  |
| BPNT1 | Subgroup_III |  |
| TMEM60 | Subgroup_III |  |
| ATP5J | Subgroup_III |  |
| MRFAP1L1 | Subgroup_III |  |
| MRPS31 | Subgroup_III |  |
| IL29 | Subgroup_III | Subgroup_I |
| NUP37 | Subgroup_III |  |
| CYB5-M | Subgroup_III |  |
| ZCCHC14 | Subgroup_III |  |
| NKRF | Subgroup_III |  |
| NMT2 | Subgroup_III |  |
| PCNP | Subgroup_III | Subgroup_I |
| P15RS | Subgroup_III |  |
| TFRC | Subgroup_III |  |
| EIF3S6 | Subgroup_III |  |
| COX4I1 | Subgroup_III |  |
| CEP135 | Subgroup_III |  |
| CLEC2B | Subgroup_III |  |
| C18orf25 | Subgroup_III |  |
| ANGEL2 | Subgroup_III |  |
| UTP11L | Subgroup_III |  |
| C19orf12 | Subgroup_III |  |
| TSEN2 | Subgroup_III |  |
| ZNF30 | Subgroup_III |  |
| TNFRSF17 | Subgroup_III |  |
| LOC285513 | Subgroup_III |  |
| C20orf174 | Subgroup_III |  |
| POLE3 | Subgroup_III |  |
| GNLY | Subgroup_III |  |
| HSPC148 | Subgroup_III |  |
| KIAA0494 | Subgroup_III |  |
| PGRMC2 | Subgroup_III |  |
| TNFSF7 | Subgroup_III | Subgroup_II |
| LOC125893 | Subgroup_III | Subgroup_I |
| LOC644096 | Subgroup_III |  |
| ABCB1 | Subgroup_III |  |
| ZNF239 | Subgroup_III |  |
| NDUFV2 | Subgroup_III |  |
| CXCR7 | Subgroup_III | Subgroup_II |
| EOMES | Subgroup_III |  |
| C17orf45 | Subgroup_III |  |
| GZMA | Subgroup_III | Subgroup_I |
| C7orf30 | Subgroup_III |  |
| PLEKHA1 | Subgroup_III |  |
| VAMP8 | Subgroup_III |  |
| TINP1 | Subgroup_III | Subgroup_I |
| SUMF2 | Subgroup_III |  |
| MGST3 | Subgroup_III |  |
| SETMAR | Subgroup_III |  |
| FLJ11305 | Subgroup_III |  |
| MRPS15 | Subgroup_III |  |
| CHST12 | Subgroup_III |  |
| MTCP1 | Subgroup_III | Subgroup_I |
| MAT2A | Subgroup_III |  |
| ARMC10 | Subgroup_III |  |
| MPP6 | Subgroup_III |  |
| COX7C | Subgroup_III |  |
| RPL30 | Subgroup_III |  |
| CD2 | Subgroup_III |  |
| PFDN1 | Subgroup_III |  |
| EIF3S6IP | Subgroup_III |  |
| ERP29 | Subgroup_III |  |
| FLJ10260 | Subgroup_III |  |
| PLA2G4B | Subgroup_III |  |
| DNAJC19 | Subgroup_III |  |
| RBM33 | Subgroup_III |  |
| ZNF101 | Subgroup_III |  |
| SIVA | Subgroup_III |  |
| CBR4 | Subgroup_III |  |
| COQ5 | Subgroup_III |  |
| LPIN1 | Subgroup_III |  |
| TMCO1 | Subgroup_III |  |
| MRPS33 | Subgroup_III |  |
| CD247 | Subgroup_III |  |
| TIMM10 | Subgroup_III |  |
| C9orf46 | Subgroup_III |  |
| C14orf112 | Subgroup_III |  |
| ZNF235 | Subgroup_III |  |
| EBI2 | Subgroup_III |  |
| NDUFB11 | Subgroup_III | Subgroup_II |
| RAB7L1 | Subgroup_III |  |
| C19orf2 | Subgroup_III | Subgroup_I |
| C6orf70 | Subgroup_III |  |
| CRTAP | Subgroup_III |  |
| C1orf131 | Subgroup_III |  |
| C12orf45 | Subgroup_III |  |
| RASSF7 | Subgroup_III |  |
| CBLB | Subgroup_III |  |
| RPL36 | Subgroup_III |  |
| TMEM14A | Subgroup_III |  |
| PPP3CC | Subgroup_III |  |
| BFAR | Subgroup_III |  |
| ZMYM1 | Subgroup_III |  |
| EIF3S3 | Subgroup_III |  |
| C20orf30 | Subgroup_III |  |
| C1orf52 | Subgroup_III |  |
| ATP5G2 | Subgroup_III |  |
| C9orf95 | Subgroup_III |  |
| MIS12 | Subgroup_III | Subgroup_I |
| CCL5 | Subgroup_III |  |
| STRA13 | Subgroup_III |  |
| MGC2463 | Subgroup_III |  |
| HCP5 | Subgroup_III |  |
| KRT10 | Subgroup_III |  |
| MRPS24 | Subgroup_III |  |
| C1orf21 | Subgroup_III |  |
| KLRC3 | Subgroup_III |  |
| C1orf82 | Subgroup_III |  |
| TRIM22 | Subgroup_III |  |
| STARD7 | Subgroup_III |  |
| TOMM22 | Subgroup_III |  |
| TMEM126A | Subgroup_III |  |
| RPL18 | Subgroup_III |  |
| CTGLF3 | Subgroup_III |  |
| GLO1 | Subgroup_III |  |
| POLR1E | Subgroup_III |  |
| ARL1 | Subgroup_III |  |
| DNASE1L2 | Subgroup_III | Subgroup_II |
| SNRPD2 | Subgroup_III |  |
| ADSL | Subgroup_III |  |
| C1orf181 | Subgroup_III |  |
| SOCS2 | Subgroup_III |  |
| RPS11 | Subgroup_III |  |
| GTPBP6 | Subgroup_III |  |
| EIF3S4 | Subgroup_III |  |
| NDUFB8 | Subgroup_III |  |
| SNRPF | Subgroup_III |  |
| FAM43A | Subgroup_III |  |
| CD3D | Subgroup_III |  |
| RPS15 | Subgroup_III | Subgroup_II |
| TMEM42 | Subgroup_III |  |
| TMEM14C | Subgroup_III | Subgroup_I |
| SLAMF1 | Subgroup_III |  |
| RPSA | Subgroup_III |  |
| FBL | Subgroup_III |  |
| MAGEE1 | Subgroup_III |  |
| RPL19 | Subgroup_III |  |
| C1orf149 | Subgroup_III |  |
| SSR4 | Subgroup_III |  |
| CUTA | Subgroup_III |  |
| DFFB | Subgroup_III |  |
| IAH1 | Subgroup_III |  |
| LOC442578 | Subgroup_III |  |
| ANKRD46 | Subgroup_III | Subgroup_I |
| HINT1 | Subgroup_III | Subgroup_I |
| E2F5 | Subgroup_III | Subgroup_I |
| RPLP0 | Subgroup_III |  |
| XBP1 | Subgroup_III |  |
| PPP1R2 | Subgroup_III |  |
| GPD1L | Subgroup_III |  |
| CLN5 | Subgroup_III |  |
| FAM107B | Subgroup_III |  |
| EIF4A2 | Subgroup_III | Subgroup_I |
| DJ122O8.2 | Subgroup_III |  |
| HIST1H4C | Subgroup_III |  |
| LOC285193 | Subgroup_III |  |
| MRPL14 | Subgroup_III |  |
| ZNF286 | Subgroup_III |  |
| C3orf26 | Subgroup_III |  |
| HSZFP36 | Subgroup_III |  |
| TCFL5 | Subgroup_III |  |
| NCOA7 | Subgroup_III |  |
| PPA1 | Subgroup_III |  |
| CD52 | Subgroup_III |  |
| MRPL34 | Subgroup_III |  |
| FAM58A | Subgroup_III |  |
| OCIAD2 | Subgroup_III |  |
| TNFSF5IP1 | Subgroup_III |  |
| ISG20L1 | Subgroup_III |  |
| NXT1 | Subgroup_III |  |
| ZNF545 | Subgroup_III |  |
| TTC19 | Subgroup_III |  |
| C3orf9 | Subgroup_III |  |
| LOC728758 | Subgroup_III |  |
| C21orf127 | Subgroup_III |  |
| FAU | Subgroup_III |  |
| DNAJC9 | Subgroup_III |  |
| SF3B5 | Subgroup_III |  |
| MTMR15 | Subgroup_III |  |
| BZW2 | Subgroup_III |  |
| SLAMF6 | Subgroup_III |  |
| LTB | Subgroup_III |  |
| CD40LG | Subgroup_III | Subgroup_II |
| GPX7 | Subgroup_III |  |
| RALA | Subgroup_III |  |
| ITK | Subgroup_III |  |
| LOC144233 | Subgroup_III |  |
| PCMTD2 | Subgroup_III |  |
| MGC14327 | Subgroup_III |  |
| RPL18A | Subgroup_III |  |
| NECAP1 | Subgroup_III |  |
| POLR2D | Subgroup_III |  |
| ECHDC2 | Subgroup_III |  |
| SEC11L1 | Subgroup_III |  |
| LAIR2 | Subgroup_III |  |
| FNBP4 | Subgroup_III |  |
| CD6 | Subgroup_III |  |
| FAM84B | Subgroup_III |  |
| LAMP3 | Subgroup_III |  |
| PRKCH | Subgroup_III |  |
| GLTSCR2 | Subgroup_III |  |
| LOC91689 | Subgroup_III |  |
| IL7R | Subgroup_III | Subgroup_II |
| GZMH | Subgroup_III |  |
| SFRS7 | Subgroup_III | Subgroup_I |
| EBAG9 | Subgroup_III |  |
| FAM62B | Subgroup_III |  |
| PRIM1 | Subgroup_III |  |
| PTCD2 | Subgroup_III |  |
| AXIIR | Subgroup_III |  |
| ASF1A | Subgroup_III |  |
| CCDC32 | Subgroup_III |  |
| SLC25A5 | Subgroup_III |  |
| CD3G | Subgroup_III |  |
| RPL17 | Subgroup_III |  |
| PUS1 | Subgroup_III |  |
| ZC3HC1 | Subgroup_III |  |
| TTC13 | Subgroup_III |  |
| KIAA1147 | Subgroup_III |  |
| SUMO3 | Subgroup_III |  |
| DYRK2 | Subgroup_III |  |
| CD96 | Subgroup_III |  |
| QSCN6L1 | Subgroup_III |  |
| SDAD1 | Subgroup_III |  |
| CUL2 | Subgroup_III |  |
| LETMD1 | Subgroup_III |  |
| BBS4 | Subgroup_III |  |
| EXOSC2 | Subgroup_III |  |
| NUP160 | Subgroup_III |  |
| ATG16L1 | Subgroup_III |  |
| PDCD2 | Subgroup_III |  |
| HIBCH | Subgroup_III |  |
| PBX4 | Subgroup_III |  |
| SLC25A26 |  | Subgroup_II |
| ANP32B |  | Subgroup_II |
| MRPS9 |  | Subgroup_II |
| ZNF77 |  | Subgroup_I |
| BTF3 |  | Subgroup_II |
| LOC654123 |  | Subgroup_II |
| MRPS2 |  | Subgroup_II |
| RPL24 |  | Subgroup_I |
| STARD5 |  | Subgroup_II |
| RPS16 |  | Subgroup_I |
| C15orf41 |  | Subgroup_I |
| CLDND2 |  | Subgroup_II |
| CD320 |  | Subgroup_II |
| GPM6B |  | Subgroup_I |
| RPS13 |  | Subgroup_I |
| MRPL21 |  | Subgroup_II |
| DNASE1L3 |  | Subgroup_I |
| RPL6 |  | Subgroup_II |
| MRPS11 |  | Subgroup_II |
| MRPL23 |  | Subgroup_II |
| RPL29 |  | Subgroup_II |
| DOCK9 |  | Subgroup_III |
| MORN3 |  | Subgroup_II |
| FLJ10120 |  | Subgroup_II |
| CD3E |  | Subgroup_II |
| DKFZp686K16132 |  | Subgroup_II |
| AXIN1 |  | Subgroup_II |
| CMAS |  | Subgroup_II |
| IL21R |  | Subgroup_II |
| ACP1 |  | Subgroup_II |
| RPL34 |  | Subgroup_I |
| C6orf192 |  | Subgroup_II |
| AHSA1 | Subgroup_I | Subgroup_III |
| HSPC159 | Subgroup_I |  |
| DREV1 | Subgroup_I |  |
| CKAP4 | Subgroup_I |  |
| FCGR1A | Subgroup_I | Subgroup_II |
| CSPG2 | Subgroup_II | Subgroup_I |
| TNFSF13B | Subgroup_II |  |
| CYP1B1 | Subgroup_II | Subgroup_I |
| DSC2 | Subgroup_II |  |
| CASP4 | Subgroup_II | Subgroup_I |
| MTPN | Subgroup_II |  |
| IVNS1ABP | Subgroup_II |  |
| TLE4 | Subgroup_II |  |
| HMGCR | Subgroup_II |  |
| SPAG9 | Subgroup_II |  |
| STAG2 | Subgroup_II | Subgroup_I |
| CPB1 | Subgroup_II | Subgroup_III |
| HLA-DRB5 | Subgroup_II |  |
| PCMTD1 | Subgroup_II | Subgroup_I |
| BAZ2B | Subgroup_II |  |
| CASP8 | Subgroup_II | Subgroup_I |
| IL1RL2 | Subgroup_II | Subgroup_III |
| KIAA1600 | Subgroup_II |  |
| E2F3 | Subgroup_II |  |
| FOS | Subgroup_II |  |
| MARCKS | Subgroup_II |  |
| CXCL5 | Subgroup_II | Subgroup_I |
| CPD | Subgroup_II |  |
| NUMB | Subgroup_II |  |
| MASK | Subgroup_II | Subgroup_I |
| EVI2A | Subgroup_II | Subgroup_I |
| NOTCH2 | Subgroup_II | Subgroup_III |
| CLEC7A | Subgroup_II |  |
| LRRFIP1 | Subgroup_II |  |
| TNFRSF12A | Subgroup_II | Subgroup_III |
| PMAIP1 | Subgroup_II | Subgroup_I |
| DOCK11 | Subgroup_II |  |
| ARHGAP9 | Subgroup_II |  |
| CXCL1 | Subgroup_II |  |
| GALNT14 | Subgroup_II | Subgroup_I |
| F2RL1 | Subgroup_II | Subgroup_I |
| RFWD2 | Subgroup_II |  |
| CLEC4D | Subgroup_II |  |
| KIAA1434 | Subgroup_II |  |
| APAF1 | Subgroup_II | Subgroup_III |
| PPP4R1 | Subgroup_II |  |
| SF1 | Subgroup_II | Subgroup_I |
| PDCD10 | Subgroup_II | Subgroup_I |
| USP1 | Subgroup_II | Subgroup_I |
| HAL | Subgroup_II |  |
| GLRX | Subgroup_II | Subgroup_I |
| HSDL2 | Subgroup_II |  |
| PICALM | Subgroup_II |  |
| CD58 | Subgroup_II | Subgroup_I |
| LRP1 | Subgroup_II | Subgroup_I |
| TNFSF13 | Subgroup_II | Subgroup_III |
| LAMP2 | Subgroup_II |  |
| SART2 | Subgroup_III |  |
| CX3CR1 | Subgroup_III | Subgroup_I |
| TNFSF8 | Subgroup_III | Subgroup_I |
| CXCL10 | Subgroup_III |  |
| CASP3 | Subgroup_III | Subgroup_I |
| TNFRSF10D | Subgroup_III | Subgroup_I |
| IL17RC | Subgroup_III | Subgroup_I |
| ATG12 | Subgroup_III |  |
| MMP10 | Subgroup_III | Subgroup_I |
| STK3 | Subgroup_III |  |
| IL6ST | Subgroup_III | Subgroup_I |
| FCGR2B | Subgroup_III | Subgroup_II |
| CASP1 | Subgroup_III | Subgroup_I |
| UHMK1 | Subgroup_III | Subgroup_II |
| C3AR1 | Subgroup_III |  |
| SELPLG | Subgroup_III | Subgroup_II |
| SLC35F5 |  | Subgroup_I |
| TOR1AIP1 |  | Subgroup_I |
| SLTM |  | Subgroup_I |
| HOOK3 |  | Subgroup_I |
| SMC4 |  | Subgroup_I |
| OSRF |  | Subgroup_I |
| CD163 |  | Subgroup_III |
| KIAA0265 |  | Subgroup_I |
| KIF11 |  | Subgroup_I |
| CNIH4 |  | Subgroup_I |
| ELF1 |  | Subgroup_I |
| LOC388284 |  | Subgroup_I |
| MFN1 |  | Subgroup_I |
| BIRC2 |  | Subgroup_I |
| MLSTD2 |  | Subgroup_I |
| C14orf106 |  | Subgroup_I |
| NKX3-1 |  | Subgroup_I |
| FLJ25006 |  | Subgroup_I |
| MTRF1 |  | Subgroup_I |
| SGK |  | Subgroup_I |
| C17orf58 |  | Subgroup_I |
| ZNF644 |  | Subgroup_I |
| BTN2A1 |  | Subgroup_I |
| ARL5B |  | Subgroup_I |
| HSD17B12 |  | Subgroup_I |
| IFIH1 |  | Subgroup_I |
| PEX11A |  | Subgroup_I |
| RNGTT |  | Subgroup_I |
| MAP3K8 |  | Subgroup_I |
| POLE2 |  | Subgroup_I |
| ING1 |  | Subgroup_I |
| BBS7 |  | Subgroup_I |
| NCOA3 |  | Subgroup_III |
| JMJD1C |  | Subgroup_I |
| CREB1 |  | Subgroup_I |
| AKAP7 |  | Subgroup_I |
| PFDN4 |  | Subgroup_I |
| MGC39497 |  | Subgroup_I |
| LY96 |  | Subgroup_I |
| RGL3 |  | Subgroup_I |
| LNX2 |  | Subgroup_I |
| FBXO11 |  | Subgroup_I |
| CPSF6 |  | Subgroup_I |
| MST4 |  | Subgroup_I |
| CCDC72 |  | Subgroup_I |
| ABHD3 |  | Subgroup_I |
| BNIP2 |  | Subgroup_I |
| ECM2 |  | Subgroup_I |
| DEGS1 |  | Subgroup_I |
| LEMD3 |  | Subgroup_I |
| IFNG |  | Subgroup_I |
| PCF11 |  | Subgroup_I |
| ACVR1 |  | Subgroup_I |
| RSBN1L |  | Subgroup_I |
| NAB1 |  | Subgroup_I |
| PAK2 |  | Subgroup_III |
| ATG5 |  | Subgroup_I |
| FLJ10726 |  | Subgroup_I |
| USP38 |  | Subgroup_I |
| RCOR1 |  | Subgroup_I |
| VCPIP1 |  | Subgroup_I |
| PHGDHL1 |  | Subgroup_I |
| TMEM55A |  | Subgroup_I |
| C20orf7 |  | Subgroup_I |
| HNMT |  | Subgroup_I |
| ACN9 |  | Subgroup_I |
| PDK4 |  | Subgroup_I |
| RCBTB2 |  | Subgroup_I |
| KLF10 |  | Subgroup_I |
| TTC32 |  | Subgroup_I |
| ENPP5 |  | Subgroup_I |
| FLJ40172 |  | Subgroup_I |
| C1GALT1C1 |  | Subgroup_I |
| CLK4 |  | Subgroup_I |
| THUMPD3 |  | Subgroup_I |
| IFIT5 |  | Subgroup_I |
| PTGER4 |  | Subgroup_I |
| COX7B |  | Subgroup_I |
| C6orf162 |  | Subgroup_I |
| CRLF3 |  | Subgroup_I |
| RIF1 |  | Subgroup_I |
| SGK3 |  | Subgroup_I |
| ZMPSTE24 |  | Subgroup_I |
| TXNL5 |  | Subgroup_I |
| RAB18 |  | Subgroup_I |
| ATP6AP2 |  | Subgroup_I |
| ADAMDEC1 |  | Subgroup_I |
| USP15 |  | Subgroup_I |
| CXCL6 |  | Subgroup_I |
| HTLF |  | Subgroup_I |
| PRKAA1 |  | Subgroup_I |
| WDR79 |  | Subgroup_I |
| ROCK1 |  | Subgroup_I |
| ST8SIA4 |  | Subgroup_I |
| AZI2 |  | Subgroup_I |
| VPS4B |  | Subgroup_I |
| TPARL |  | Subgroup_I |
| NETO2 |  | Subgroup_I |
| ZNF136 |  | Subgroup_I |
| ZNF701 |  | Subgroup_I |
| CRSP3 |  | Subgroup_I |
| XRN1 |  | Subgroup_I |
| GCA |  | Subgroup_I |
| ZNF429 |  | Subgroup_I |
| KYNU |  | Subgroup_I |
| CP110 |  | Subgroup_I |
| SLC35A1 |  | Subgroup_I |
| ZCCHC6 |  | Subgroup_III |
| RMI1 |  | Subgroup_I |
| SLC38A2 |  | Subgroup_I |
| MFSD1 |  | Subgroup_I |
| SLC22A4 |  | Subgroup_II |
| ZNF445 |  | Subgroup_I |
| SHCBP1 | Subgroup_II |  |
| GABARAPL2 | Subgroup_II |  |
| LOC123688 | Subgroup_II |  |
| ESCO1 | Subgroup_II | Subgroup_I |
| LOC130355 | Subgroup_II |  |
| RCP9 | Subgroup_II |  |
| ZNRF2 | Subgroup_II |  |
| HECA | Subgroup_II |  |
| ACTN2 | Subgroup_II |  |
| BIN2 | Subgroup_II |  |
| GPKOW | Subgroup_II |  |
| FTHL11 | Subgroup_II |  |
| ANKRD27 | Subgroup_II |  |
| TMEM50A | Subgroup_II |  |
| PPP3CA | Subgroup_II |  |
| GDI2 | Subgroup_II | Subgroup_I |
| HS2ST1 | Subgroup_II | Subgroup_I |
| LOC440926 | Subgroup_II |  |
| GALNT3 | Subgroup_II |  |
| FAM72A | Subgroup_II |  |
| USP49 | Subgroup_II | Subgroup_I |
| LSDP5 | Subgroup_II |  |
| DMC1 | Subgroup_II | Subgroup_I |
| CLTC | Subgroup_II |  |
| B3GNT5 | Subgroup_II | Subgroup_I |
| ARFGEF1 | Subgroup_II |  |
| CXorf21 | Subgroup_II | Subgroup_I |
| APOBEC3A | Subgroup_II | Subgroup_I |
| FTHL12 | Subgroup_II |  |
| TLR1 | Subgroup_II |  |
| SRGN | Subgroup_II |  |
| YTHDF3 | Subgroup_II | Subgroup_I |
| GMFG | Subgroup_II |  |
| C1orf176 | Subgroup_II | Subgroup_I |
| ARHGAP18 | Subgroup_II | Subgroup_I |
| SSTR2 | Subgroup_II | Subgroup_I |
| C8orf45 | Subgroup_II | Subgroup_I |
| EIF2AK4 | Subgroup_II |  |
| HIF1A | Subgroup_II | Subgroup_I |
| PHF23 | Subgroup_II |  |
| NS4ATP2 | Subgroup_II | Subgroup_I |
| SMPDL3A | Subgroup_II |  |
| F11R | Subgroup_II |  |
| IRF4 | Subgroup_II |  |
| PIK3C3 | Subgroup_II |  |
| SLC39A6 | Subgroup_II |  |
| DTWD2 | Subgroup_II | Subgroup_I |
| C8orf37 | Subgroup_II |  |
| PPARBP | Subgroup_II |  |
| KIAA1751 | Subgroup_II | Subgroup_I |
| LOC90586 | Subgroup_II | Subgroup_I |
| LOC150763 | Subgroup_II |  |
| C11orf63 | Subgroup_II |  |
| SRPK2 | Subgroup_II |  |
| ZNF669 | Subgroup_II |  |
| CENTB2 | Subgroup_II |  |
| CYB5R4 | Subgroup_II |  |
| ANXA2P1 | Subgroup_II |  |
| PTPLAD2 | Subgroup_II | Subgroup_I |
| PKN2 | Subgroup_II |  |
| HCCS | Subgroup_II | Subgroup_I |
| C11orf38 | Subgroup_II |  |
| RPL7L1 | Subgroup_II |  |
| FCGR3A | Subgroup_II |  |
| CTR9 | Subgroup_II | Subgroup_I |
| DUXAP3 | Subgroup_II |  |
| EDD1 | Subgroup_II | Subgroup_I |
| CYCSL1 | Subgroup_II | Subgroup_I |
| C15orf29 | Subgroup_II | Subgroup_I |
| IL17RD | Subgroup_II | Subgroup_I |
| CSNK1G2 | Subgroup_II |  |
| FBXO30 | Subgroup_II | Subgroup_I |
| C10orf58 | Subgroup_II |  |
| STK17B | Subgroup_II |  |
| ATP11B | Subgroup_II | Subgroup_I |
| CDC42 | Subgroup_II |  |
| GMPR2 | Subgroup_II |  |
| FCGR3B | Subgroup_II |  |
| KIAA0738 | Subgroup_II |  |
| DPYD | Subgroup_II |  |
| FLJ46109 | Subgroup_II | Subgroup_I |
| HLA-G | Subgroup_II |  |
| SNAPC1 | Subgroup_II | Subgroup_I |
| FCGR2C | Subgroup_II |  |
| C6orf170 | Subgroup_II |  |
| YY1 | Subgroup_II | Subgroup_I |
| CYP51A1 | Subgroup_II | Subgroup_I |
| HDAC4 | Subgroup_II | Subgroup_I |
| DHRS8 | Subgroup_II |  |
| IPP | Subgroup_II |  |
| P4HA1 | Subgroup_II | Subgroup_I |
| GPR1 | Subgroup_II | Subgroup_I |
| BCL2A1 | Subgroup_II | Subgroup_I |
| CCBE1 | Subgroup_II | Subgroup_I |
| FAM40B | Subgroup_II |  |
| GOLGB1 | Subgroup_II |  |
| TMEM65 | Subgroup_II |  |
| ET | Subgroup_II |  |
| ZNF682 | Subgroup_II | Subgroup_I |
| SAR1A | Subgroup_II | Subgroup_I |
| TOP1P2 | Subgroup_II |  |
| ZFP36L1 | Subgroup_II | Subgroup_I |
| COPZ2 | Subgroup_II |  |
| LOC143543 | Subgroup_II | Subgroup_I |
| PPM1K | Subgroup_II | Subgroup_I |
| PCDHB9 | Subgroup_II |  |
| LOC339745 | Subgroup_II | Subgroup_I |
| KBTBD7 | Subgroup_II |  |
| CNBP | Subgroup_II | Subgroup_I |
| FAM76A | Subgroup_II | Subgroup_I |
| NCF2 | Subgroup_II |  |
| SERINC1 | Subgroup_II | Subgroup_I |
| SERP1 | Subgroup_II | Subgroup_I |
| TFAP2A | Subgroup_II |  |
| WRB | Subgroup_II | Subgroup_I |
| REL | Subgroup_II |  |
| FLJ46309 | Subgroup_II | Subgroup_I |
| SPAST | Subgroup_II | Subgroup_I |
| LAPTM4A | Subgroup_II |  |
| IGFL3 | Subgroup_II |  |
| LOC388955 | Subgroup_II |  |
| SLC35E1 | Subgroup_II | Subgroup_I |
| SULT1A1 | Subgroup_II |  |
| ZNF223 | Subgroup_II |  |
| EVI2B | Subgroup_II |  |
| PIK3AP1 | Subgroup_II | Subgroup_I |
| CNGB1 | Subgroup_II |  |
| SLC5A8 | Subgroup_II | Subgroup_I |
| UBLCP1 | Subgroup_II | Subgroup_I |
| ZADH1 | Subgroup_II | Subgroup_I |
| PNPT1 | Subgroup_II |  |
| TTRAP | Subgroup_II | Subgroup_I |
| COP1 | Subgroup_II | Subgroup_I |
| FLJ20273 | Subgroup_II |  |
| LEP | Subgroup_II | Subgroup_I |
| METTL7A | Subgroup_II | Subgroup_I |
| PCID1 | Subgroup_II | Subgroup_I |
| MINPP1 | Subgroup_II | Subgroup_I |
| IL1R1 | Subgroup_II |  |
| ZNF557 | Subgroup_II |  |
| HEBP2 | Subgroup_II | Subgroup_I |
| MOSPD2 | Subgroup_II |  |
| C1D | Subgroup_II |  |
| HIST1H2AD | Subgroup_II |  |
| OAS3 | Subgroup_II |  |
| SYAP1 | Subgroup_II | Subgroup_I |
| FAM126B | Subgroup_II |  |
| DKFZp762I137 | Subgroup_II |  |
| UBE2D1 | Subgroup_II |  |
| SEMA3C | Subgroup_II | Subgroup_I |
| HAT1 | Subgroup_II | Subgroup_I |
| CEP27 | Subgroup_II |  |
| U2AF1 | Subgroup_II |  |
| PHF3 | Subgroup_II |  |
| FAM103A1 | Subgroup_II | Subgroup_I |
| HK2 | Subgroup_II |  |
| BNIP3L | Subgroup_II | Subgroup_I |
| PABPC1 | Subgroup_II | Subgroup_I |
| FTHL2 | Subgroup_II |  |
| LOC649946 | Subgroup_II | Subgroup_I |
| DENND4A | Subgroup_II |  |
| DUSP11 | Subgroup_II | Subgroup_I |
| ACTR3 | Subgroup_II | Subgroup_I |
| DENR | Subgroup_II |  |
| SNAP23 | Subgroup_II |  |
| NFYB | Subgroup_II |  |
| TOPORS | Subgroup_II | Subgroup_I |
| TUBB4Q | Subgroup_II |  |
| GNA13 | Subgroup_II | Subgroup_I |
| DAPP1 | Subgroup_II |  |
| ZNF430 | Subgroup_II |  |
| FLJ37357 | Subgroup_II |  |
| APPL | Subgroup_II | Subgroup_I |
| MCTS1 | Subgroup_II | Subgroup_I |
| RRM2B | Subgroup_II | Subgroup_I |
| RRN3 | Subgroup_II | Subgroup_I |
| LOC440093 | Subgroup_II |  |
| TRA1P2 | Subgroup_II |  |
| MGC11102 | Subgroup_II | Subgroup_I |
| DYNC1LI1 | Subgroup_II |  |
| PIK3CA | Subgroup_II | Subgroup_I |
| C8orf52 | Subgroup_II | Subgroup_I |
| SFPQ | Subgroup_II |  |
| IKBKB | Subgroup_II | Subgroup_I |
| SAMSN1 | Subgroup_II | Subgroup_I |
| LOC440686 | Subgroup_II |  |
| DCTN4 | Subgroup_II |  |
| MAP3K7IP2 | Subgroup_II |  |
| LCOR | Subgroup_II | Subgroup_I |
| ERGIC2 | Subgroup_II | Subgroup_I |
| RBMXL1 | Subgroup_II |  |
| SLBP | Subgroup_II | Subgroup_I |
| PAPSS1 | Subgroup_II | Subgroup_I |
| C2orf12 | Subgroup_II |  |
| AMY2B | Subgroup_II | Subgroup_I |
| TOP1P1 | Subgroup_II |  |
| UEVLD | Subgroup_II |  |
| SPC24 | Subgroup_II |  |
| AGTPBP1 | Subgroup_II | Subgroup_I |
| TANK | Subgroup_II | Subgroup_I |
| RAD21 | Subgroup_II | Subgroup_I |
| HMGB2 | Subgroup_II |  |
| LOC497661 | Subgroup_II | Subgroup_I |
| C18orf26 | Subgroup_II |  |
| LGALS3 | Subgroup_II |  |
| FAM63B | Subgroup_II |  |
| ATXN1 | Subgroup_II |  |
| FRY | Subgroup_II |  |
| MARS | Subgroup_II |  |
| PLDN | Subgroup_II | Subgroup_I |
| EXOC8 | Subgroup_II |  |
| CAT | Subgroup_II | Subgroup_I |
| CDC5L | Subgroup_II |  |
| GIMAP7 | Subgroup_II | Subgroup_I |
| NMI | Subgroup_II | Subgroup_I |
| SACM1L | Subgroup_II | Subgroup_I |
| C4orf34 | Subgroup_II |  |
| LOC653490 | Subgroup_II |  |
| VPS26 | Subgroup_II | Subgroup_I |
| XRCC4 | Subgroup_II |  |
| MGC23909 | Subgroup_II | Subgroup_I |
| SDFR1 | Subgroup_II |  |
| GIMAP1 | Subgroup_II | Subgroup_I |
| ALPP | Subgroup_II | Subgroup_I |
| LSM6 | Subgroup_II | Subgroup_I |
| C1orf43 | Subgroup_II | Subgroup_I |
| AIRE | Subgroup_II |  |
| UBE4A | Subgroup_II | Subgroup_I |
| RAD51 | Subgroup_II |  |
| LOC401019 | Subgroup_II | Subgroup_I |
| PCAF | Subgroup_II |  |
| MTF2 | Subgroup_II | Subgroup_I |
| AMY1A | Subgroup_II |  |
| BMP8B | Subgroup_II |  |
| MEGF9 | Subgroup_II |  |
| LOC441168 | Subgroup_II | Subgroup_I |
| MAN2B2 | Subgroup_II |  |
| ECHDC1 | Subgroup_II | Subgroup_I |
| HNRPH1 | Subgroup_II | Subgroup_I |
| DOPEY2 | Subgroup_II |  |
| TMSB4X | Subgroup_II |  |
| WSB2 | Subgroup_II | Subgroup_I |
| TMSB4Y | Subgroup_II |  |
| TP53INP1 | Subgroup_II |  |
| C8orf53 | Subgroup_II | Subgroup_I |
| CIR | Subgroup_II |  |
| ITM2A | Subgroup_II | Subgroup_I |
| JMJD1A | Subgroup_II | Subgroup_I |
| IRF2 | Subgroup_II |  |
| LYSMD3 | Subgroup_II |  |
| NKAP | Subgroup_II | Subgroup_I |
| PTMA | Subgroup_II | Subgroup_I |
| HIAT1 | Subgroup_II | Subgroup_I |
| MTMR6 | Subgroup_II | Subgroup_I |
| ADI1 | Subgroup_II |  |
| MCFD2 | Subgroup_II |  |
| RAB6A | Subgroup_II |  |
| SHOC2 | Subgroup_II |  |
| KIAA1033 | Subgroup_II | Subgroup_I |
| CLK1 | Subgroup_II | Subgroup_I |
| FAM49B | Subgroup_II | Subgroup_I |
| RAP1B | Subgroup_II | Subgroup_I |
| C7orf11 | Subgroup_II | Subgroup_I |
| FBXO38 | Subgroup_II |  |
| IDI1 | Subgroup_II | Subgroup_I |
| PPIG | Subgroup_II | Subgroup_I |
| LOC440157 | Subgroup_II |  |
| RPS28 | Subgroup_II | Subgroup_I |
| C1orf63 | Subgroup_II | Subgroup_I |
| ANP32A | Subgroup_II |  |
| YIPF4 | Subgroup_II |  |
| YIPF5 | Subgroup_II | Subgroup_I |
| PPP2CB | Subgroup_II | Subgroup_I |
| RABGEF1 | Subgroup_II |  |
| PPP1R12A | Subgroup_II | Subgroup_I |
| VAPA | Subgroup_II |  |
| CKAP2 | Subgroup_II | Subgroup_I |
| KPNA2 | Subgroup_II | Subgroup_I |
| PRPF18 | Subgroup_II | Subgroup_I |
| UBE2W | Subgroup_II |  |
| CENPC1 | Subgroup_II |  |
| FLI1 | Subgroup_II | Subgroup_I |
| G3BP1 | Subgroup_II | Subgroup_I |
| SPTLC1 | Subgroup_II | Subgroup_I |
| GGH | Subgroup_II | Subgroup_I |
| TXNDC9 | Subgroup_II | Subgroup_I |
| PRKAR1A | Subgroup_II |  |
| EIF3S10 | Subgroup_II | Subgroup_I |
| USP6 | Subgroup_II | Subgroup_I |
| PRO1853 | Subgroup_II | Subgroup_I |
| PPIB | Subgroup_II |  |
| HERPUD2 | Subgroup_II | Subgroup_I |
| SUMO1 | Subgroup_II | Subgroup_I |
| OCIAD1 | Subgroup_II |  |
| TSPAN2 | Subgroup_II | Subgroup_I |
| RPL23 | Subgroup_II | Subgroup_I |
| SMBP | Subgroup_II |  |
| LARP4 | Subgroup_II |  |
| CAST | Subgroup_II |  |
| MRP63 | Subgroup_II | Subgroup_I |
| ZFAND6 | Subgroup_II | Subgroup_I |
| SP3 | Subgroup_II | Subgroup_I |
| SP4 | Subgroup_II | Subgroup_I |
| DOCK8 | Subgroup_II |  |
| IL18 | Subgroup_II |  |
| C6orf62 | Subgroup_II | Subgroup_I |
| AFTIPHILIN | Subgroup_II |  |
| C6orf211 | Subgroup_II | Subgroup_I |
| OMA1 | Subgroup_II | Subgroup_I |
| TSC22D2 | Subgroup_II | Subgroup_I |
| RANGNRF | Subgroup_II | Subgroup_I |
| KLF5 | Subgroup_II |  |
| COMMD8 | Subgroup_II | Subgroup_I |
| NEDD9 | Subgroup_II |  |
| SLK | Subgroup_II |  |
| ARL6IP1 | Subgroup_II | Subgroup_I |
| ADAM10 | Subgroup_II | Subgroup_I |
| C9orf77 | Subgroup_II | Subgroup_I |
| SNW1 | Subgroup_II |  |
| SNX10 | Subgroup_II | Subgroup_I |
| PPP1R15B | Subgroup_II | Subgroup_I |
| GNAI3 | Subgroup_II |  |
| WDR51B | Subgroup_II |  |
| C14orf19 | Subgroup_II | Subgroup_I |
| NACAP1 | Subgroup_II | Subgroup_I |
| C14orf11 | Subgroup_II | Subgroup_I |
| TSNAX | Subgroup_II | Subgroup_I |
| LOC196394 | Subgroup_II | Subgroup_I |
| ALG13 | Subgroup_II | Subgroup_I |
| TXNL1 | Subgroup_II | Subgroup_I |
| RBBP4 | Subgroup_II | Subgroup_I |
| FRG1 | Subgroup_II | Subgroup_I |
| RPL37A | Subgroup_II | Subgroup_I |
| ARPP-19 | Subgroup_II | Subgroup_I |
| MRPL3 | Subgroup_II | Subgroup_I |
| SMAP | Subgroup_II | Subgroup_I |
| CCNH | Subgroup_II | Subgroup_I |
| RANBP9 | Subgroup_II |  |
| C16orf69 | Subgroup_II |  |
| C20orf19 | Subgroup_II | Subgroup_I |
| ABAT | Subgroup_II |  |
| ZNF320 | Subgroup_II |  |
| MORF4L1 | Subgroup_II | Subgroup_I |
| PGAM4 | Subgroup_II |  |
| HNRPC | Subgroup_II |  |
| LSM5 | Subgroup_II | Subgroup_I |
| PNPLA8 | Subgroup_II | Subgroup_I |
| UQCRC2 | Subgroup_II | Subgroup_I |
| FKBP3 | Subgroup_II | Subgroup_I |
| SELT | Subgroup_II | Subgroup_I |
| CD68 | Subgroup_II |  |
| SEC24D | Subgroup_II |  |
| SEC24A | Subgroup_II | Subgroup_I |
| CD302 | Subgroup_II | Subgroup_I |
| KIAA1604 | Subgroup_II | Subgroup_I |
| CDK5R1 | Subgroup_II |  |
| OSBPL11 | Subgroup_II |  |
| SYNJ1 | Subgroup_II | Subgroup_I |
| ADH4 | Subgroup_II |  |
| CD164 | Subgroup_II | Subgroup_I |
| EDEM3 | Subgroup_II |  |
| C12orf35 | Subgroup_II |  |
| RPN2 | Subgroup_II | Subgroup_I |
| DAAM1 | Subgroup_II | Subgroup_I |
| MGC40405 | Subgroup_II | Subgroup_I |
| PSMC6 | Subgroup_II | Subgroup_I |
| CXorf39 | Subgroup_II | Subgroup_I |
| STX7 | Subgroup_II |  |
| SPAG1 | Subgroup_II |  |
| HMG1L1 | Subgroup_II | Subgroup_I |
| PDCD7 | Subgroup_II |  |
| WSB1 | Subgroup_II | Subgroup_I |
| CEP57 | Subgroup_II | Subgroup_I |
| DNTTIP2 | Subgroup_II | Subgroup_I |
| RBM7 | Subgroup_II | Subgroup_I |
| SNORD36A | Subgroup_II | Subgroup_I |
| SMNDC1 | Subgroup_II | Subgroup_I |
| SLC25A32 | Subgroup_II |  |
| LOC653709 | Subgroup_II | Subgroup_I |
| ANXA4 | Subgroup_II | Subgroup_I |
| FLJ12716 | Subgroup_II | Subgroup_I |
| PAK1IP1 | Subgroup_II | Subgroup_I |
| ZBTB2 | Subgroup_II | Subgroup_I |
| ZNF226 | Subgroup_II | Subgroup_I |
| ZNF23 | Subgroup_II |  |
| SMARCA5 | Subgroup_II | Subgroup_I |
| ARPC5 | Subgroup_II | Subgroup_I |
| ASCIZ | Subgroup_II | Subgroup_I |
| FLJ39599 | Subgroup_II |  |
| ROD1 | Subgroup_II |  |
| USP8 | Subgroup_II | Subgroup_I |
| TM9SF2 | Subgroup_II | Subgroup_I |
| SMC3 | Subgroup_II | Subgroup_I |
| RG9MTD1 | Subgroup_II | Subgroup_I |
| KLHL2 | Subgroup_II |  |
| TNFSF14 | Subgroup_II |  |
| PDIA3P | Subgroup_II | Subgroup_I |
| PRKCI | Subgroup_II |  |
| DCTN6 | Subgroup_II |  |
| TATDN1 | Subgroup_II | Subgroup_I |
| DMXL1 | Subgroup_II | Subgroup_I |
| GPBP1L1 | Subgroup_II |  |
| DC2 | Subgroup_II | Subgroup_I |
| FCMD | Subgroup_II |  |
| CLIP1 | Subgroup_II |  |
| PPP2R3C | Subgroup_II | Subgroup_I |
| LOC441244 | Subgroup_II |  |
| ME2 | Subgroup_II |  |
| RNPC3 | Subgroup_II | Subgroup_I |
| ZBTB11 | Subgroup_II | Subgroup_I |
| PARP9 | Subgroup_II | Subgroup_I |
| EAF1 | Subgroup_II | Subgroup_I |
| WDR23 | Subgroup_II |  |
| RECQL | Subgroup_II |  |
| TMEM30A | Subgroup_II | Subgroup_I |
| ATF1 | Subgroup_II | Subgroup_I |
| PPM2C | Subgroup_II | Subgroup_I |
| FLJ90013 | Subgroup_II | Subgroup_I |
| SENP6 | Subgroup_II |  |
| SKAP2 | Subgroup_II |  |
| SFRS11 | Subgroup_II | Subgroup_I |
| SFRS12 | Subgroup_II | Subgroup_I |
| RAB33B | Subgroup_II | Subgroup_I |
| ST6GALNAC3 | Subgroup_II | Subgroup_I |
| WIPF1 | Subgroup_II |  |
| FYTTD1 | Subgroup_II | Subgroup_I |
| FLJ11712 | Subgroup_II | Subgroup_I |
| CIDEC | Subgroup_II |  |
| FLJ12118 | Subgroup_II |  |
| TAF9 | Subgroup_II | Subgroup_I |
| GTF2H2 | Subgroup_II | Subgroup_I |
| SEC23A | Subgroup_II |  |
| LYPLA1 | Subgroup_II | Subgroup_I |
| GCLC | Subgroup_II |  |
| HIGD1A | Subgroup_II | Subgroup_I |
| NAP1L1 | Subgroup_II | Subgroup_I |
| LY75 | Subgroup_II | Subgroup_I |
| NLK | Subgroup_II | Subgroup_I |
| HMGB1 | Subgroup_II | Subgroup_I |
| PRKAR2B | Subgroup_II |  |
| C14orf108 | Subgroup_II | Subgroup_I |
| CNOT8 | Subgroup_II | Subgroup_I |
| SFRS3 | Subgroup_II | Subgroup_I |
| NDUFA5 | Subgroup_II |  |
| RSRC2 | Subgroup_II | Subgroup_I |
| GPBP1 | Subgroup_II | Subgroup_I |
| TIMM8B | Subgroup_II | Subgroup_I |
| NME2 | Subgroup_II |  |
| FAHD2A | Subgroup_II |  |
| C1orf25 | Subgroup_II | Subgroup_I |
| HSPA4 | Subgroup_II |  |
| BLVRB | Subgroup_II |  |
| S100A12 | Subgroup_II |  |
| LYST | Subgroup_II |  |
| CBX1 | Subgroup_II |  |
| DECR1 | Subgroup_II |  |
| C15orf5 | Subgroup_II |  |
| SF3B14 | Subgroup_II | Subgroup_I |
| WDSOF1 | Subgroup_II | Subgroup_I |
| NAT13 | Subgroup_II | Subgroup_I |
| ABHD13 | Subgroup_II |  |
| EIF4E | Subgroup_II | Subgroup_I |
| FLJ23518 | Subgroup_II |  |
| RNPC2 | Subgroup_II |  |
| NXT2 | Subgroup_II | Subgroup_I |
| GALNT7 | Subgroup_II |  |
| FAS | Subgroup_II | Subgroup_I |
| TSN | Subgroup_II |  |
| GTF2H1 | Subgroup_II | Subgroup_I |
| SURB7 | Subgroup_II | Subgroup_I |
| CCDC91 | Subgroup_II | Subgroup_I |
| TRAT1 | Subgroup_II |  |
| GLT8D1 | Subgroup_II |  |
| FNDC3A | Subgroup_II | Subgroup_I |
| DICER1 | Subgroup_II | Subgroup_I |
| C1orf9 | Subgroup_II | Subgroup_I |
| THEX1 | Subgroup_II |  |
| FAM8A1 | Subgroup_II | Subgroup_I |
| ATG3 | Subgroup_II |  |
| MTDH | Subgroup_II | Subgroup_I |
| GBP3 | Subgroup_II | Subgroup_I |
| RFK | Subgroup_II | Subgroup_I |
| TRAPPC6B | Subgroup_II | Subgroup_I |
| HNRPLL | Subgroup_II | Subgroup_I |
| R3HDM1 | Subgroup_II | Subgroup_I |
| VPS37A | Subgroup_II | Subgroup_I |
| C7orf28B | Subgroup_II |  |
| RBM34 | Subgroup_II | Subgroup_I |
| C1orf156 | Subgroup_II | Subgroup_I |
| C4orf32 | Subgroup_II | Subgroup_I |
| CDC37L1 | Subgroup_II |  |
| NT5DC1 | Subgroup_II |  |
| DUSP6 | Subgroup_II |  |
| ATP2B1 | Subgroup_II | Subgroup_I |
| LRRC40 | Subgroup_II | Subgroup_I |
| C6orf66 | Subgroup_II | Subgroup_I |
| MSL2L1 | Subgroup_II |  |
| SPHAR | Subgroup_II | Subgroup_I |
| CRSP9 | Subgroup_II | Subgroup_I |
| UAP1 | Subgroup_II | Subgroup_I |
| FLJ34969 | Subgroup_II | Subgroup_I |
| DLD | Subgroup_II |  |
| C6orf203 | Subgroup_II |  |
| LIN7C | Subgroup_II | Subgroup_I |
| C17orf32 | Subgroup_II |  |
| GOPC | Subgroup_II | Subgroup_I |
| DDHD1 | Subgroup_II |  |
| RANBP2 | Subgroup_II |  |
| FGFR1OP2 | Subgroup_II | Subgroup_I |
| ZNF12 | Subgroup_II | Subgroup_I |
| APLP2 | Subgroup_II |  |
| RAB8B | Subgroup_II | Subgroup_I |
| GPR65 | Subgroup_II | Subgroup_I |
| LIG4 | Subgroup_II |  |
| C14orf129 | Subgroup_II | Subgroup_I |
| LRRC59 | Subgroup_II |  |
| HHEX | Subgroup_II | Subgroup_I |
| EED | Subgroup_II | Subgroup_I |
| FCHO2 | Subgroup_II | Subgroup_I |
| GIYD2 | Subgroup_II |  |
| FAM122A | Subgroup_II |  |
| S100PBP | Subgroup_II |  |
| MRPL47 | Subgroup_II | Subgroup_I |
| ZNF75A | Subgroup_II |  |
| UFM1 | Subgroup_II | Subgroup_I |
| TERF1 | Subgroup_II |  |
| STCH | Subgroup_II | Subgroup_I |
| PPP4R2 | Subgroup_II |  |
| ZNF140 | Subgroup_II |  |
| HMGN2 | Subgroup_II | Subgroup_I |
| SNX14 | Subgroup_II | Subgroup_I |
| ASCC3 | Subgroup_II |  |
| CHD1 | Subgroup_II | Subgroup_I |
| RHOB | Subgroup_II |  |
| FBXO5 | Subgroup_II | Subgroup_I |
| RAP2C | Subgroup_II | Subgroup_I |
| WDR47 | Subgroup_II |  |
| TOPBP1 | Subgroup_II | Subgroup_I |
| CDKN1B | Subgroup_II | Subgroup_I |
| C17orf39 | Subgroup_II |  |
| LOC126295 | Subgroup_II |  |
| ASB3 | Subgroup_II |  |
| FLJ11151 | Subgroup_II |  |
| C4orf16 | Subgroup_II |  |
| LMO2 | Subgroup_II | Subgroup_I |
| NT5C2 | Subgroup_II |  |
| MGC88387 | Subgroup_II |  |
| XPO4 | Subgroup_II | Subgroup_I |
| LOC653328 | Subgroup_II | Subgroup_I |
| RNF149 | Subgroup_II |  |
| SORD | Subgroup_II |  |
| BLNK | Subgroup_II |  |
| ARHGAP12 | Subgroup_II | Subgroup_I |
| LOC653906 | Subgroup_II |  |
| FTHL3 | Subgroup_II |  |
| WDR44 | Subgroup_II |  |
| RPS6KC1 | Subgroup_II |  |
| C1GALT1 | Subgroup_II | Subgroup_I |
| C9orf80 | Subgroup_II |  |
| UHRF2 | Subgroup_II | Subgroup_I |
| C5orf28 | Subgroup_II | Subgroup_I |
| CTNNB1 | Subgroup_II |  |
| SMC5L1 | Subgroup_II |  |
| CREBL2 | Subgroup_II |  |
| ZNF43 | Subgroup_II |  |
| ZDHHC2 | Subgroup_II |  |
| PRKY | Subgroup_II |  |
| MGC4399 | Subgroup_II |  |
| ZNF567 | Subgroup_II |  |
| RPS4Y1 | Subgroup_II | Subgroup_III |
| GRAMD1C | Subgroup_II |  |
| POFUT1 | Subgroup_II |  |
| PCYOX1 | Subgroup_II |  |
| HIP2 | Subgroup_II |  |
| C9orf52 | Subgroup_II |  |
| BTNL8 | Subgroup_II |  |
| UBE2NL | Subgroup_II |  |
| AOC3 | Subgroup_II |  |
| TDP1 | Subgroup_II |  |
| ACOT2 | Subgroup_II |  |
| ITFG1 | Subgroup_II |  |
| CETN3 | Subgroup_II |  |
| BIRC3 | Subgroup_II | Subgroup_I |
| XPNPEP3 | Subgroup_II |  |
| GNB4 | Subgroup_II |  |
| HNRPCL1 | Subgroup_II |  |
| ADAMTS20 | Subgroup_II | Subgroup_I |
| LOC285359 | Subgroup_II |  |
| TBL1XR1 | Subgroup_II |  |
| PJA2 | Subgroup_II |  |
| OGFRL1 | Subgroup_II |  |
| C9orf64 | Subgroup_II | Subgroup_I |
| DCHS1 | Subgroup_II |  |
| GNPDA2 | Subgroup_II |  |
| EDEM1 | Subgroup_II |  |
| A2M | Subgroup_II | Subgroup_I |
| MORF4 | Subgroup_II |  |
| FBXL3 | Subgroup_II |  |
| BZW1 | Subgroup_II |  |
| XPA | Subgroup_II |  |
| EPB41L2 | Subgroup_II |  |
| PDCD4 | Subgroup_II | Subgroup_I |
| DDX3Y | Subgroup_II |  |
| ATXN10 | Subgroup_II |  |
| SNHG8 | Subgroup_II |  |
| LDHAL6A | Subgroup_II |  |
| LOC643366 | Subgroup_II |  |
| CYP4F2 | Subgroup_II |  |
| SUDS3 | Subgroup_II |  |
| SLC44A4 | Subgroup_II |  |
| CHST1 | Subgroup_II |  |
| CICK0721Q.1 | Subgroup_II |  |
| LOC202134 | Subgroup_II |  |
| KLF15 | Subgroup_II |  |
| DLEU7 | Subgroup_II |  |
| CLK2P | Subgroup_II |  |
| HSCB | Subgroup_II | Subgroup_I |
| SH3YL1 | Subgroup_II | Subgroup_I |
| KCTD7 | Subgroup_II |  |
| EIF1AY | Subgroup_II |  |
| LOC644685 | Subgroup_II |  |
| ZNF85 | Subgroup_II | Subgroup_I |
| TMEM19 | Subgroup_II |  |
| POLR2K | Subgroup_II |  |
| GRK5 | Subgroup_II |  |
| DLEU2L | Subgroup_II |  |
| RPS4Y2 | Subgroup_II | Subgroup_III |
| SCYE1 | Subgroup_II |  |
| RASSF6 | Subgroup_II |  |
| GPR171 | Subgroup_II |  |
| DCK | Subgroup_II |  |
| FAM120A | Subgroup_II |  |
| ZBTB1 | Subgroup_II |  |
| ASB8 | Subgroup_II |  |
| POLH | Subgroup_II |  |
| MYO3B | Subgroup_II |  |
| POLI | Subgroup_II |  |
| RB1CC1 | Subgroup_II |  |
| RNU2 | Subgroup_II |  |
| SLC14A1 | Subgroup_II |  |
| K6HF | Subgroup_II |  |
| ZNF131 | Subgroup_II |  |
| SYNJ2BP | Subgroup_II | Subgroup_I |
| MGC4728 | Subgroup_II | Subgroup_I |
| MUT | Subgroup_II |  |
| RFX4 | Subgroup_II |  |
| CBWD1 | Subgroup_II |  |
| HUS1B | Subgroup_II |  |
| HRSP12 | Subgroup_II |  |
| MSH2 | Subgroup_II |  |
| MYNN | Subgroup_II |  |
| IFRD1 | Subgroup_II |  |
| SMAD5 | Subgroup_II | Subgroup_I |
| KCNK6 | Subgroup_II |  |
| CCDC117 | Subgroup_II |  |
| PDK1 | Subgroup_II | Subgroup_III |
| ARFIP1 | Subgroup_II |  |
| TMEM70 | Subgroup_II |  |
| CTSS | Subgroup_II |  |
| GCLM | Subgroup_II |  |
| ZNF294 | Subgroup_II |  |
| FCF1 | Subgroup_II |  |
| MBOAT2 | Subgroup_II |  |
| ALCAM | Subgroup_II | Subgroup_I |
| DDX19A | Subgroup_II |  |
| RBM3 | Subgroup_II | Subgroup_I |
| DCLRE1C | Subgroup_II |  |
| FLJ11184 | Subgroup_II | Subgroup_I |
| XRN2 | Subgroup_II |  |
| BLZF1 | Subgroup_II |  |
| FLJ13611 | Subgroup_II |  |
| GPD2 | Subgroup_II |  |
| DYNLT3 | Subgroup_II |  |
| HMGN3 | Subgroup_II |  |
| KRTAP10-11 | Subgroup_II |  |
| MTMR4 | Subgroup_II |  |
| ANKRD44 | Subgroup_II | Subgroup_I |
| UBXD4 | Subgroup_II | Subgroup_I |
| MBNL1 | Subgroup_II | Subgroup_I |
| AIM2 | Subgroup_II |  |
| ZRF1 | Subgroup_II |  |
| CHORDC1 | Subgroup_II |  |
| KCTD12 | Subgroup_II |  |
| TXNL6 | Subgroup_II |  |
| SYCP3 | Subgroup_II |  |
| UBE3B | Subgroup_II |  |
| GTPBP2 | Subgroup_II |  |
| EID2B | Subgroup_II | Subgroup_I |
| MMP14 | Subgroup_II | Subgroup_I |
| DPCR1 | Subgroup_II |  |
| ADAM17 | Subgroup_II |  |
| CXorf44 | Subgroup_II |  |
| HTR2A | Subgroup_II | Subgroup_I |
| MGC16186 | Subgroup_II |  |
| FTS | Subgroup_II | Subgroup_I |
| TTC8 | Subgroup_II |  |
| UCRC | Subgroup_II |  |
| SUMO1P3 | Subgroup_II |  |
| ATPBD1C | Subgroup_II |  |
| UBE1C | Subgroup_II |  |
| GNG2 | Subgroup_II |  |
| TMEM38B | Subgroup_II |  |
| PDF | Subgroup_II |  |
| PEX13 | Subgroup_II |  |
| C12orf30 | Subgroup_II |  |
| CCDC104 | Subgroup_II |  |
| BTNL2 | Subgroup_II |  |
| DKFZp564K142 | Subgroup_II | Subgroup_I |
| LOC134145 | Subgroup_II |  |
| HNRPU | Subgroup_II | Subgroup_I |
| LOC653033 | Subgroup_II |  |
| NUDT21 | Subgroup_II |  |
| DTWD1 | Subgroup_II |  |
| RHBDL2 | Subgroup_II |  |
| VDAC2 | Subgroup_II |  |
| C14orf4 | Subgroup_II | Subgroup_I |
| SAS10 | Subgroup_II |  |
| OSBPL8 | Subgroup_II |  |
| ZNF345 | Subgroup_II |  |
| NACAL | Subgroup_II |  |
| RNASEN | Subgroup_II |  |
| C14orf85 | Subgroup_II | Subgroup_I |
| AHSA2 | Subgroup_II | Subgroup_I |
| POLK | Subgroup_II |  |
| PLAGL1 | Subgroup_II |  |
| XRCC2 | Subgroup_II | Subgroup_I |
| ZNF708 | Subgroup_II |  |
| RICTOR | Subgroup_II |  |
| OR1J1 | Subgroup_II |  |
| RPGR | Subgroup_II |  |
| C9orf30 | Subgroup_II |  |
| KIAA0408 | Subgroup_II |  |
| MCM8 | Subgroup_II |  |
| C15orf21 | Subgroup_II |  |
| RNASEL | Subgroup_II |  |
| PDP2 | Subgroup_II |  |
| PSEN1 | Subgroup_II |  |
| ITM2B | Subgroup_II |  |
| GALNS | Subgroup_II |  |
| VEZT | Subgroup_II |  |
| RPL21 | Subgroup_II | Subgroup_I |
| N4BP2 | Subgroup_II | Subgroup_I |
| RBM25 | Subgroup_II | Subgroup_I |
| YOD1 | Subgroup_II |  |
| SOAT1 | Subgroup_II |  |
| FKBP14 | Subgroup_II | Subgroup_I |
| ZNF69 | Subgroup_II |  |
| ZFY | Subgroup_II |  |
| SYNE1 | Subgroup_II |  |
| SMCY | Subgroup_II |  |
| ZNF681 | Subgroup_II | Subgroup_I |
| ZFR | Subgroup_II | Subgroup_I |
| TMCC1 | Subgroup_II |  |
| TMEM154 | Subgroup_II |  |
| TMEM156 | Subgroup_II | Subgroup_I |
| SPN | Subgroup_II |  |
| HEXDC | Subgroup_II |  |
| JOSD3 | Subgroup_II | Subgroup_I |
| SSB | Subgroup_II |  |
| HSPC268 | Subgroup_II | Subgroup_I |
| DDB2 | Subgroup_II |  |
| TMEM157 | Subgroup_II |  |
| NIPSNAP3A | Subgroup_II |  |
| CPEB3 | Subgroup_II |  |
| MAK | Subgroup_II |  |
| VPS41 | Subgroup_II |  |
| CAPN7 | Subgroup_II |  |
| TUBD1 | Subgroup_II | Subgroup_I |
| CCDC43 | Subgroup_II |  |
| LILRB1 | Subgroup_II |  |
| EFCAB2 | Subgroup_II |  |
| MGC29891 | Subgroup_II |  |
| CYorf15A | Subgroup_II |  |
| FAM45A | Subgroup_II | Subgroup_I |
| OR52K2 | Subgroup_II |  |
| KIAA1641 | Subgroup_II |  |
| KPNA3 | Subgroup_II | Subgroup_I |
| MARS2 | Subgroup_II |  |
| ING4 | Subgroup_II |  |
| KIAA0103 | Subgroup_II |  |
| TNFSF15 | Subgroup_II |  |
| ZNF493 | Subgroup_II |  |
| LOC90321 | Subgroup_II |  |
| SLC4A5 | Subgroup_II |  |
| SPCS2 | Subgroup_II |  |
| USP9Y | Subgroup_II |  |
| KRT9 | Subgroup_II |  |
| CHPT1 | Subgroup_II |  |
| BRWD2 | Subgroup_II | Subgroup_I |
| POTE2 | Subgroup_II |  |
| STAR | Subgroup_II |  |
| HIVEP2 | Subgroup_II |  |
| C2orf33 | Subgroup_II | Subgroup_I |
| LAT1-3TM | Subgroup_II | Subgroup_I |
| PPCS | Subgroup_II | Subgroup_I |
| ZCCHC10 | Subgroup_II |  |
| HNRPH3 | Subgroup_II | Subgroup_I |
| ZNF577 | Subgroup_II | Subgroup_I |
| ZNF600 | Subgroup_II |  |
| ABI1 | Subgroup_II |  |
| UTY | Subgroup_II |  |
| TPRKB | Subgroup_II |  |
| MPHOSPH10 | Subgroup_II | Subgroup_I |
| C2orf21 | Subgroup_II |  |
| ATG4C | Subgroup_II |  |
| KIAA1279 | Subgroup_II |  |
| RANBP6 | Subgroup_II |  |
| GDPD1 | Subgroup_II |  |
| ELOVL5 | Subgroup_II | Subgroup_I |
| POLR2J3 | Subgroup_II |  |
| LOC441743 | Subgroup_II | Subgroup_I |
| LRRK1 | Subgroup_II |  |
| RAXL1 | Subgroup_II |  |
| C1QL2 | Subgroup_II |  |
| TMEM106B | Subgroup_II | Subgroup_I |
| ZNF14 | Subgroup_II |  |
| CGI-09 | Subgroup_II |  |
| C9orf85 | Subgroup_II |  |
| PRPF39 | Subgroup_II |  |
| NUBPL | Subgroup_II | Subgroup_I |
| C21orf24 | Subgroup_II |  |
| PIGX | Subgroup_II |  |
| ARF6 | Subgroup_II |  |
| WDR74 | Subgroup_II |  |
| LOC402057 | Subgroup_II | Subgroup_I |
| RPL23AP13 | Subgroup_II | Subgroup_I |
| POLD3 | Subgroup_II |  |
| C12orf31 | Subgroup_II | Subgroup_I |
| ZBTB8OS | Subgroup_II | Subgroup_I |
| MRPL19 | Subgroup_II | Subgroup_I |
| MYO18A | Subgroup_II |  |
| SDHD | Subgroup_II | Subgroup_I |
| BCAS2 | Subgroup_II |  |
| RPL23AP7 | Subgroup_II |  |
| NUDCD2 | Subgroup_II | Subgroup_I |
| ARFGAP3 | Subgroup_II |  |
| SEC8L1 | Subgroup_II |  |
| MGC3265 | Subgroup_II |  |
| CDAN1 | Subgroup_II |  |
| NIT1 | Subgroup_II |  |
| PRRG4 | Subgroup_II |  |
| OR3A4 | Subgroup_II |  |
| FAM119A | Subgroup_II | Subgroup_I |
| ZNF483 | Subgroup_II | Subgroup_I |
| NDUFS2 | Subgroup_II |  |
| FLJ44124 | Subgroup_II | Subgroup_I |
| MTERFD2 | Subgroup_II |  |
| FLJ45256 | Subgroup_II |  |
| PLA2G2D | Subgroup_II |  |
| IL10 | Subgroup_II |  |
| NLRP8 | Subgroup_II | Subgroup_I |
| FLJ13273 | Subgroup_II |  |
| DBR1 | Subgroup_II | Subgroup_I |
| CSF2RA | Subgroup_II |  |
| SUMO2 | Subgroup_II | Subgroup_I |
| RPL7A | Subgroup_II | Subgroup_I |
| TXNDC4 | Subgroup_II | Subgroup_I |
| GKAP1 | Subgroup_II |  |
| IL1RAP | Subgroup_II |  |
| RPL14 | Subgroup_II | Subgroup_I |
| ZNF33A | Subgroup_II |  |
| LOC401252 | Subgroup_II |  |
| RPL7 | Subgroup_II | Subgroup_I |
| CASP6 | Subgroup_II | Subgroup_I |
| TCP1 | Subgroup_II |  |
| MAN1A1 | Subgroup_II |  |
| LOC112714 | Subgroup_II |  |
| ATAD1 | Subgroup_II | Subgroup_I |
| MAD2L1 | Subgroup_II |  |
| ZC3H7A | Subgroup_II |  |
| FLJ38973 | Subgroup_II | Subgroup_I |
| B3GAT1 | Subgroup_II |  |
| BRIP1 | Subgroup_II |  |
| COPS2 | Subgroup_II |  |
| KIAA1370 | Subgroup_II | Subgroup_I |
| PI4K2B | Subgroup_II | Subgroup_I |
| COG3 | Subgroup_II | Subgroup_I |
| KRCC1 | Subgroup_II | Subgroup_I |
| LOC440348 | Subgroup_II |  |
| MOBKL1A | Subgroup_II |  |
| CREM | Subgroup_II |  |
| SLAMF7 | Subgroup_II | Subgroup_I |
| SERAC1 | Subgroup_II |  |
| RDH11 | Subgroup_II |  |
| GCNT1 | Subgroup_II |  |
| NNT | Subgroup_II | Subgroup_I |
| IL31 | Subgroup_III | Subgroup_I |
| PSMA4 |  | Subgroup_I |
| ARRDC4 |  | Subgroup_I |
| PCNA |  | Subgroup_I |
| GMNN |  | Subgroup_I |
| IBTK |  | Subgroup_I |
| MPHOSPH6 |  | Subgroup_I |
| CHD6 |  | Subgroup_I |
| POLR3F |  | Subgroup_I |
| ID2 |  | Subgroup_I |
| FH |  | Subgroup_I |
| C4orf18 |  | Subgroup_I |
| LOC728643 |  | Subgroup_I |
| ACSL4 |  | Subgroup_I |
| HDCMA18P |  | Subgroup_I |
| LOC201725 |  | Subgroup_I |
| SKIV2L2 |  | Subgroup_I |
| AVEN |  | Subgroup_I |
| SHROOM4 |  | Subgroup_I |
| GLRX2 |  | Subgroup_I |
| EIF1AX |  | Subgroup_I |
| FAM102B |  | Subgroup_I |
| PTGES3 |  | Subgroup_I |
| C3orf28 |  | Subgroup_I |
| ATF4 |  | Subgroup_I |
| ZNF431 |  | Subgroup_I |
| SFRS1 |  | Subgroup_I |
| TIPRL |  | Subgroup_I |
| ALDH1A1 |  | Subgroup_I |
| UBE1L2 |  | Subgroup_I |
| RANBP1 |  | Subgroup_I |
| NDUFB10 |  | Subgroup_I |
| FAM14A |  | Subgroup_I |
| LOC113444 |  | Subgroup_I |
| USP25 |  | Subgroup_I |
| SERPINB2 |  | Subgroup_I |
| KTN1 |  | Subgroup_I |
| PSMD14 |  | Subgroup_I |
| CUL4A |  | Subgroup_I |
| DDOST |  | Subgroup_I |
| ZFP1 |  | Subgroup_I |
| MYC |  | Subgroup_I |
| NARS |  | Subgroup_I |
| ARL8B |  | Subgroup_I |
| ZC3H15 |  | Subgroup_I |
| ZNF124 |  | Subgroup_I |
| C6orf120 |  | Subgroup_I |
| MRPL10 |  | Subgroup_I |
| SNORD36C |  | Subgroup_I |
| TMED7 |  | Subgroup_I |
| ATP1B3 |  | Subgroup_I |
| C14orf166 |  | Subgroup_I |
| NBPF14 |  | Subgroup_I |
| GIMAP2 |  | Subgroup_I |
| VAMP4 |  | Subgroup_I |
| CTSO |  | Subgroup_I |
| MRPL15 |  | Subgroup_I |
| FAM35A |  | Subgroup_I |
| RTCD1 |  | Subgroup_I |
| HSPE1 |  | Subgroup_I |
| SLFN11 |  | Subgroup_I |
| QTRTD1 |  | Subgroup_I |
| ZNF318 |  | Subgroup_I |
| BLVRA |  | Subgroup_I |
| LOC653566 |  | Subgroup_I |
| SFRS10 |  | Subgroup_I |
| ZNF302 |  | Subgroup_I |
| MGC4268 |  | Subgroup_I |
| SLC25A13 |  | Subgroup_I |
| TBC1D9 |  | Subgroup_I |
| CCT6AP1 |  | Subgroup_I |
| SEC11L3 |  | Subgroup_I |
| BTG3 |  | Subgroup_I |
| IER3IP1 |  | Subgroup_I |
| PSMA3 |  | Subgroup_I |
| SLC25A16 |  | Subgroup_I |
| DNAJC1 |  | Subgroup_I |
| PTPN22 |  | Subgroup_I |
| RSAD1 |  | Subgroup_I |
| CROP |  | Subgroup_I |
| C12orf11 |  | Subgroup_I |
| NUS1 |  | Subgroup_I |
| RBMX2 |  | Subgroup_I |
| SBDS |  | Subgroup_I |
| C11orf61 |  | Subgroup_I |
| C2orf7 |  | Subgroup_I |
| HNRPA1L-2 |  | Subgroup_I |
| FAM98A |  | Subgroup_I |
| MRPL48 |  | Subgroup_I |
| KLRC2 |  | Subgroup_I |
| VRK2 |  | Subgroup_I |
| RABL2A |  | Subgroup_I |
| KIAA0423 |  | Subgroup_I |
| OTUD6B |  | Subgroup_I |
| PTBP2 |  | Subgroup_I |
| SLC25A20 |  | Subgroup_I |
| ZNF313 |  | Subgroup_I |
| ATP5C1 |  | Subgroup_I |
| CD2AP |  | Subgroup_I |
| CAND1 |  | Subgroup_I |
| TMEM17 |  | Subgroup_I |
| CCDC58 |  | Subgroup_I |
| DUSP8 |  | Subgroup_I |
| ZNF322A |  | Subgroup_I |
| C5orf14 |  | Subgroup_I |
| MS4A1 |  | Subgroup_I |
| PDGFC |  | Subgroup_I |
| GRIPAP1 |  | Subgroup_I |
| SET |  | Subgroup_I |
| ISOC1 |  | Subgroup_I |
| HCG2P7 |  | Subgroup_I |
| BTN3A2 |  | Subgroup_I |
| HYPK |  | Subgroup_I |
| C21orf6 |  | Subgroup_I |
| CIAO1 |  | Subgroup_I |
| KIAA0831 |  | Subgroup_I |
| DDX17 |  | Subgroup_I |
| KCNH6 |  | Subgroup_I |
| SNTA1 |  | Subgroup_I |
| EIF4G2 |  | Subgroup_I |
| DNAJB14 |  | Subgroup_I |
| NARG1L |  | Subgroup_I |
| CHRNA5 |  | Subgroup_I |
| ALG11 |  | Subgroup_I |
| IGJ |  | Subgroup_I |
| C1orf31 |  | Subgroup_I |
| ATPBD4 |  | Subgroup_I |
| ORC3L |  | Subgroup_I |
| C13orf10 |  | Subgroup_I |
| FLJ90396 |  | Subgroup_I |
| CTSW |  | Subgroup_I |
| C5orf5 |  | Subgroup_I |
| CNOT7 |  | Subgroup_I |
| TSPAN13 |  | Subgroup_I |
| CDC7 |  | Subgroup_I |
| ADNP |  | Subgroup_I |
| PIGM |  | Subgroup_I |
| CROT |  | Subgroup_I |
| MGC13017 |  | Subgroup_I |
| BRDG1 |  | Subgroup_I |
| C14orf145 |  | Subgroup_I |
| CCNG2 |  | Subgroup_I |
| BXDC2 |  | Subgroup_I |
| GBP1 |  | Subgroup_I |
| KIF21A |  | Subgroup_I |
| ORMDL2 |  | Subgroup_I |
| LMOD3 |  | Subgroup_I |
| NGLY1 |  | Subgroup_I |
| C14orf100 |  | Subgroup_I |
| CCNG1 |  | Subgroup_I |
| DDX51 |  | Subgroup_I |
| ZNF91 |  | Subgroup_I |
| RBM13 |  | Subgroup_I |
| PEBP1 |  | Subgroup_I |
| CD48 |  | Subgroup_I |
| TRIM2 |  | Subgroup_I |
| PRSS23 |  | Subgroup_I |
| BST2 |  | Subgroup_I |
| C12orf48 |  | Subgroup_I |
| NOP5/NOP58 |  | Subgroup_I |
| RASGRP1 |  | Subgroup_I |
| LDHB |  | Subgroup_I |
| CSTF2T |  | Subgroup_I |
| GARNL1 |  | Subgroup_I |
| SC4MOL |  | Subgroup_I |
| MALT1 |  | Subgroup_I |
| CXorf26 |  | Subgroup_I |
| ADD3 |  | Subgroup_I |
| ACY1L2 |  | Subgroup_I |
| OFD1 |  | Subgroup_I |
| PNN |  | Subgroup_I |
| HCFC2 |  | Subgroup_I |
| RKHD2 |  | Subgroup_I |
| TMEM128 |  | Subgroup_I |
| PDHA1 |  | Subgroup_I |
| FAM113A |  | Subgroup_I |
| LMO4 |  | Subgroup_I |
| CRY1 |  | Subgroup_I |
| LOC153364 |  | Subgroup_I |
| CMPK |  | Subgroup_I |
| UBQLN2 |  | Subgroup_I |
| RIC8B |  | Subgroup_I |
| KLRC1 |  | Subgroup_I |
| ALG5 |  | Subgroup_I |
| GOLGA |  | Subgroup_I |
| ZNF181 |  | Subgroup_I |
| DPP4 |  | Subgroup_I |
| LAP3 |  | Subgroup_I |
| SENP7 |  | Subgroup_I |
| CENPJ |  | Subgroup_I |
| ARL16 |  | Subgroup_I |
| TRIT1 |  | Subgroup_I |
| TDRD1 |  | Subgroup_I |
| ZNF330 |  | Subgroup_I |
| ZNF331 |  | Subgroup_I |
| SMC6L1 |  | Subgroup_I |
| GBAS |  | Subgroup_I |
| LOC116236 |  | Subgroup_I |
| HNRPA2B1 |  | Subgroup_I |
| PSIP1 |  | Subgroup_I |
| ZNF652 |  | Subgroup_I |
| ZNF394 |  | Subgroup_I |
| NUCB2 |  | Subgroup_I |
| C17orf40 |  | Subgroup_I |
| BTBD1 |  | Subgroup_I |
| GOLT1B |  | Subgroup_I |
| RNUXA |  | Subgroup_I |
| IFT74 |  | Subgroup_I |
| C10orf88 |  | Subgroup_I |
| TMEM14B |  | Subgroup_I |
| RIN2 |  | Subgroup_I |
| LOC653314 |  | Subgroup_I |
| LRRC47 |  | Subgroup_I |
| SSR3 |  | Subgroup_I |
| RBX1 |  | Subgroup_I |
| PECI |  | Subgroup_I |
| ZNF611 |  | Subgroup_I |
| LGALS2 |  | Subgroup_I |
| DSTN |  | Subgroup_I |
| CCL4L1 |  | Subgroup_I |
| CDC42SE2 |  | Subgroup_I |
| CD1D |  | Subgroup_I |
| TAF7 |  | Subgroup_I |
| TAF1B |  | Subgroup_I |
| ARMC1 |  | Subgroup_I |
| NDUFAB1 |  | Subgroup_I |
| C10orf137 |  | Subgroup_I |
| THAP1 |  | Subgroup_I |
| LOC132321 |  | Subgroup_I |
| ZNF539 |  | Subgroup_I |
| NACA |  | Subgroup_I |
| PUS3 |  | Subgroup_I |
| MAPKAPK5 |  | Subgroup_I |
| C17orf25 |  | Subgroup_I |
| HDHD2 |  | Subgroup_I |
| ZNF420 |  | Subgroup_I |
| PPA2 |  | Subgroup_I |
| TARS |  | Subgroup_I |
| SLC33A1 |  | Subgroup_I |
| PRDM1 |  | Subgroup_I |
| ORMDL1 |  | Subgroup_I |
| LOC133619 |  | Subgroup_I |
| ZNF146 |  | Subgroup_I |
| TM2D3 |  | Subgroup_I |
| UTP15 |  | Subgroup_I |
| ACAA2 |  | Subgroup_I |
| SNORA32 |  | Subgroup_I |
| AASDHPPT |  | Subgroup_I |
| UGDH |  | Subgroup_I |
| MGA |  | Subgroup_I |
| TRIM56 |  | Subgroup_I |
| WASPIP |  | Subgroup_I |
| ASB1 |  | Subgroup_I |
| KLHDC2 |  | Subgroup_I |
| MGC12965 |  | Subgroup_I |
| C12orf23 |  | Subgroup_I |
| C12orf62 |  | Subgroup_I |
| PTPLB |  | Subgroup_I |
| SACS |  | Subgroup_I |
| AMY1B |  | Subgroup_I |
| ATP11C |  | Subgroup_I |
| ATP5J2 |  | Subgroup_I |
| SS18L1 |  | Subgroup_I |
| BXDC5 |  | Subgroup_I |
| FLJ30596 |  | Subgroup_I |
| FLJ20097 |  | Subgroup_I |
| ETS1 |  | Subgroup_I |
| ARL13B |  | Subgroup_I |
| SMAD7 |  | Subgroup_I |
| SLC39A8 |  | Subgroup_I |
| CPNE8 |  | Subgroup_I |
| SAV1 |  | Subgroup_I |
| SLC12A2 |  | Subgroup_I |
| CXorf40B |  | Subgroup_I |
| FVT1 |  | Subgroup_I |
| MBD4 |  | Subgroup_I |
| C16orf53 |  | Subgroup_I |
| KLRF1 |  | Subgroup_I |
| LOC283345 |  | Subgroup_I |
| SLC38A6 |  | Subgroup_I |
| METTL6 |  | Subgroup_I |
| NOL8 |  | Subgroup_I |
| MGC2803 |  | Subgroup_I |
| KLF4 |  | Subgroup_I |
| PMS1 |  | Subgroup_I |
| TUBE1 |  | Subgroup_I |
| DEK |  | Subgroup_I |
| LOC134997 |  | Subgroup_I |
| CITED2 |  | Subgroup_I |
| RBBP7 |  | Subgroup_I |
| SCYL1BP1 |  | Subgroup_I |
