# Supplementary figures and images for: Consensus clustering of gene expression profiles in peripheral blood of acute ischemic stroke patients
Source: Front Neurol. 2022 Aug 5;13:937501. doi: 10.3389/fneur.2022.937501 (PMC9388856; doi:10.3389/fneur.2022.937501)

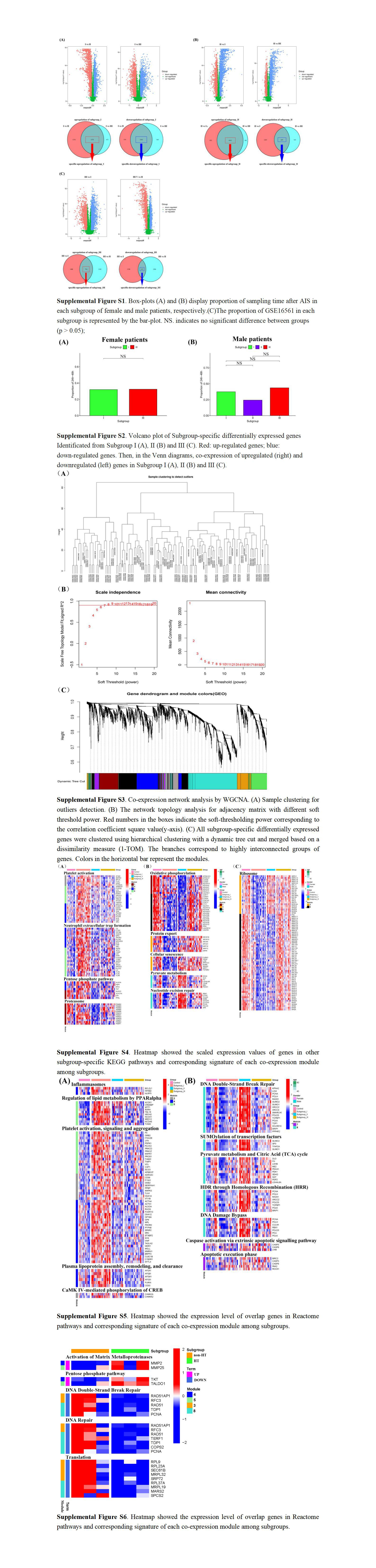

Supplement: Supplementary file 2 [file Image_1.JPEG]
